# Hepatocyte-specific CCAAT/enhancer binding protein α restricts liver fibrosis progression

**DOI:** 10.1172/JCI166731

**Published:** 2024-04-01

**Authors:** Tingting Yan, Nana Yan, Yangliu Xia, Vorthon Sawaswong, Xinxin Zhu, Henrique Bregolin Dias, Daisuke Aibara, Shogo Takahashi, Keisuke Hamada, Yoshifumi Saito, Guangming Li, Hui Liu, Hualong Yan, Thomas J. Velenosi, Kristopher W. Krausz, Jing Huang, Shioko Kimura, Yaron Rotman, Aijuan Qu, Haiping Hao, Frank J. Gonzalez

**Affiliations:** 1 Cancer Innovation Laboratory, Center for Cancer Research, National Cancer Institute, National Institutes of Health, Bethesda, Maryland, USA.; 2 State Key Laboratory of Natural Medicines, Laboratory of Metabolic Regulation and Drug Target Discovery, China Pharmaceutical University, Nanjing, China.; 3 Department of Physiology and Pathophysiology, School of Basic Medical Sciences, Capital Medical University, Key Laboratory of Remodeling-Related Cardiovascular Diseases, Ministry of Education, and Beijing Key Laboratory of Metabolic Disorder-Related Cardiovascular Diseases, Beijing, China.; 4 General Surgery Center and; 5 Department of Pathology, Beijing YouAn Hospital, Capital Medical University, Beijing, China.; 6 Cancer and Stem Cell Epigenetics, Laboratory of Cancer Biology and Genetics, Center for Cancer Research, National Cancer Institute and; 7 Liver and Energy Metabolism Section, Liver Diseases Branch, National Institute of Diabetes and Digestive and Kidney Diseases, NIH, Bethesda, Maryland, USA.

**Keywords:** Hepatology, Fibrosis, Macrophages, Transcription

## Abstract

Metabolic dysfunction–associated steatohepatitis (MASH) — previously described as nonalcoholic steatohepatitis (NASH) — is a major driver of liver fibrosis in humans, while liver fibrosis is a key determinant of all-cause mortality in liver disease independent of MASH occurrence. CCAAT/enhancer binding protein α (CEBPA), as a versatile ligand-independent transcriptional factor, has an important function in myeloid cells, and is under clinical evaluation for cancer therapy. CEBPA is also expressed in hepatocytes and regulates glucolipid homeostasis; however, the role of hepatocyte-specific CEBPA in modulating liver fibrosis progression is largely unknown. Here, hepatic CEBPA expression was found to be decreased during MASH progression both in humans and mice, and hepatic *CEBPA* mRNA was negatively correlated with MASH fibrosis in the human liver. *Cebpa*^ΔHep^ mice had markedly enhanced liver fibrosis induced by a high-fat, high-cholesterol, high-fructose diet or carbon tetrachloride. Temporal and spatial hepatocyte-specific CEBPA loss at the progressive stage of MASH in *Cebpa*^ΔHep,ERT2^ mice functionally promoted liver fibrosis. Mechanistically, hepatocyte CEBPA directly repressed *Spp1* transactivation to reduce the secretion of osteopontin, a fibrogenesis inducer of hepatic stellate cells. Forced hepatocyte-specific CEBPA expression reduced MASH-associated liver fibrosis. These results demonstrate an important role for hepatocyte-specific CEBPA in liver fibrosis progression, and may help guide the therapeutic discoveries targeting hepatocyte CEBPA for the treatment of liver fibrosis.

## Abstract 2

Hepatocyte CEBPA is gradually lost during nonalcoholic steatohepatitis in humans, which leads to hepatocyte-derived osteopontin release that in turn enhances liver fibrosis progression.

## Graphical abstract

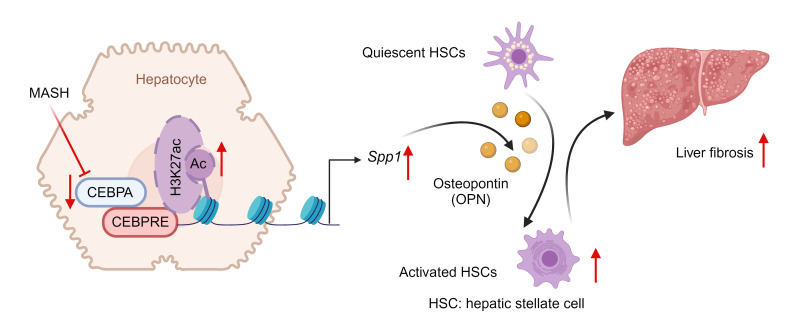

## Introduction

Growing clinical evidence revealed that liver fibrosis is a main determinant of outcomes or all-cause mortality in liver disease (1–6). Metabolic dysfunction–associated steatotic liver disease (MASLD), previously known as nonalcoholic fatty liver disease (NAFLD), could progress to metabolic dysfunction–associated steatohepatitis (MASH), previously known as nonalcoholic steatohepatitis (NASH), accompanied with liver fibrosis occurrence (7). Although multiple parallel insults via crosstalk among organs or cells were proposed to explain the pathogenesis of fibrosis progression, the molecular mechanisms underlying fibrosis progression remain incompletely understood and no US FDA-approved pharmacotherapies are presently available (8–10).

CCAAT/enhancer binding protein α (CEBPA) was initially found to modulate myeloid cell differentiation and oncogenesis (11). MTL-CEBPA is a first-in-human myeloid modifier saRNA therapeutic that improves hepatocellular carcinoma outcome by suppressing myeloid cells (12–14). CEBPA was also demonstrated to modulate the progression of hepatocellular carcinogenesis by using global CEBPA knock-in mice or MTL-CEBPA in preclinical models (12, 15). Physiologically, CEBPA controls hepatocyte maturation and liver function as revealed by CEBPA-KO embryonic livers from global CEBPA-KO mice; global CEBPA-KO mice die as neonates (16, 17). Until now, the pathological function of cell-specific CEBPA in influencing the progression of various liver diseases is still largely unexplored. While CEBPA is expressed in hepatocytes and is a modulator of glucolipid homeostasis (18, 19), whether and how hepatocyte-specific CEBPA alters the progression of liver fibrosis remains to be explored. Herein, by using a hepatocyte-specific constitutive or inducible CEBPA-deficient mouse strain in combination with adeno-associated virus serotype 8–forced (AAV8-forced) CEBPA overexpression, the pathological function of hepatocyte-specific CEBPA in regulating liver fibrosis was examined and the mechanism was identified.

## Results

### Hepatic CEBPA expression is decreased by MASH and tracks with MASH fibrosis.

**Figure 1:**
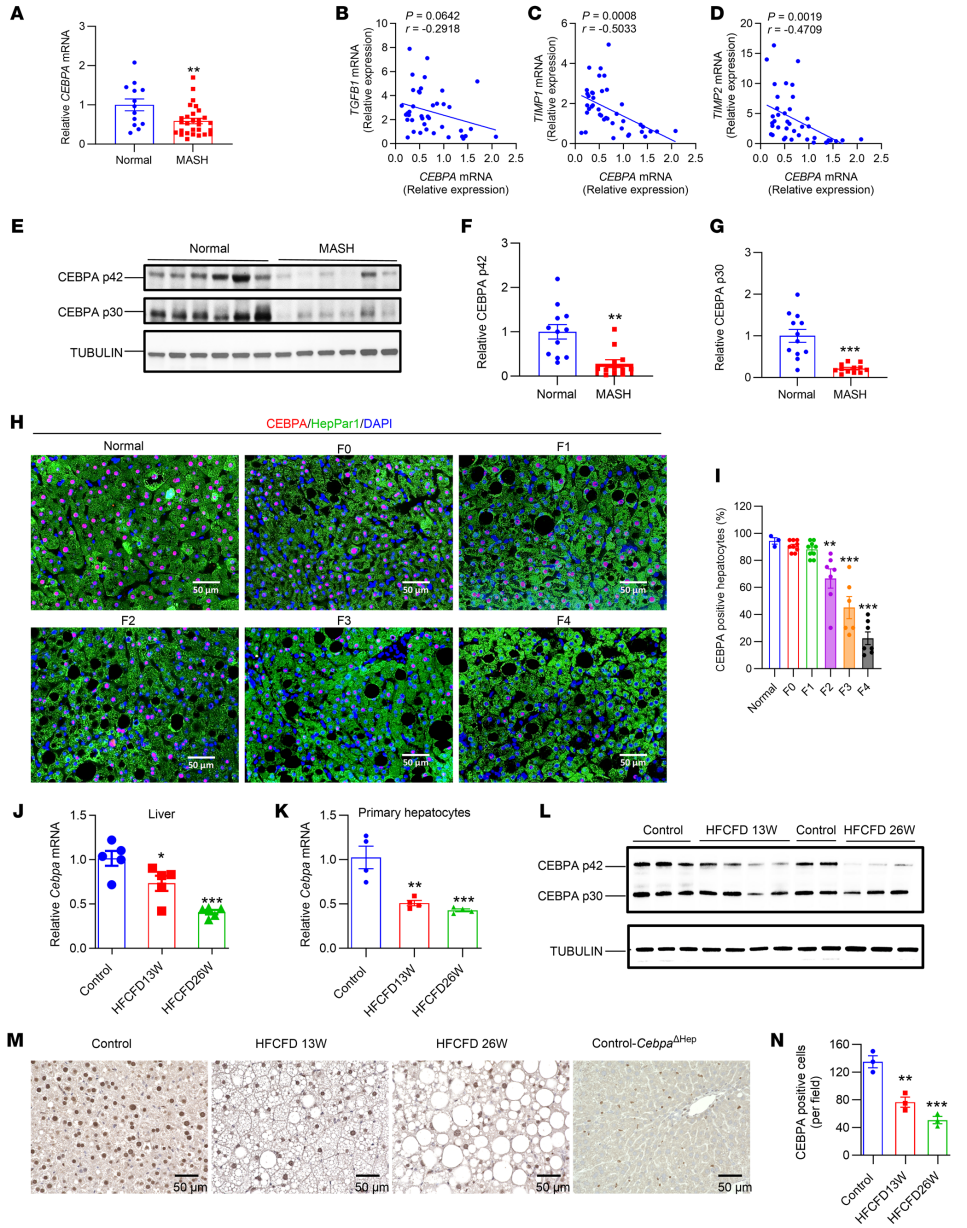
Hepatic CEBPA expression is decreased by MASH and tracks with human liver fibrosis. (**A**) *CEBPA* mRNA in human livers. *n* = 13 for normal group, and *n* = 28 for MASH group. (**B**–**D**) Correlation of *CEBPA* mRNA with fibrosis gene mRNAs in human livers by nonparametric Pearson’s test. (**E**–**G**) Representative Western blot of CEBPA p42 and p30 protein in human livers (**E**) and quantitation (**F**–**G**, *n* = 12). (**H** and **I**) Representative images of CEBPA (red), hepatocyte marker HepPar1 (green), and DAPI (blue) immunofluorescence in liver biopsies from patients with histologically normal livers, F0-4 MASLD livers and quantitation of the percentage of CEBPA positive cells among HepPar1 positive cells (pink indicates red nuclear CEBPA merged with blue DAPI; *n* = 3 for control normal livers, *n* = 9 for F0, *n* = 9 for F1, *n* = 7 for F2, *n* = 6 for F3 and *n* = 7 for F4 MASLD livers). (**J**–**N**) *Cebpa* mRNA in liver (**J**, *n* = 5) and primary hepatocytes (**K**, *n* = 4), representative liver CEBPA p42 and p30 protein (**L**), representative CEBPA IHC staining (**M**) and quantitation (**N**, *n* = 3) of C57BL/6N mice fed HFCFD for 13 or 26 weeks. Data represent mean ± SEM. *P < 0.05, **P < 0.01, ***P < 0.001 by 2-tailed unpaired student’s *t* test for **A**, **F**, and **G**, while 1-way ANOVA followed by Dunnett’s multiple-comparisons test for **I**–**K** and **N**, compared with control group. Scale bar: 50 μm.

The role of hepatic CEBPA in MASH fibrosis progression was investigated by first examining published microarray data (E-MEXP-3291 [see Supplemental Methods; supplemental material available online with this article; https://doi.org/10.1172/JCI166731DS1]). Human liver *CEBPA* mRNA was decreased while mRNAs involved in inflammation and fibrosis were increased in MASH livers (previously described as NASH livers) but not in simple steatosis livers, and several fibrosis gene mRNAs were negatively correlated with *CEBPA* mRNA (Supplemental Figure 1, A–C). In humans, *CEBPA* mRNA was decreased in MASH livers and negatively correlated with fibrosis markers, accompanied by decreased hepatic CEBPA protein levels (Figure 1, A–G), while hepatic inflammation- and fibrosis-related mRNAs were increased in MASH, indicating, as expected, that liver fibrosis is associated with MASH in humans (Supplemental Figure 1D). Furthermore, immunofluorescence staining showed that CEBPA in hepatocytes was gradually lost during MASH progression in patients (Figure 1, H–I). Consistently, *Cebpa* mRNA was time-dependently decreased in the livers and hepatocytes of mice fed a high-fat, high-cholesterol, high-fructose diet (HFCFD) for 13 or 26 weeks during the development of liver fibrosis, accompanied by decreased liver CEBPA protein, especially the 42 kD isoform (Figure 1, J–L and Supplemental Figure 1E). IHC analyses revealed a reduction of hepatic CEBPA during MASH progression, with the livers of hepatocyte-specific *Cebpa*-KO (*Cebpa*^ΔHep^) mice used as a negative control for the CEBPA antibody (Figure 1, M and N). Similarly, *Cebpa* mRNA was markedly lower in livers from 12-week HFCFD-fed *ob*/*ob* mice (Supplemental Figure 1F). Thus, hepatic CEBPA expression was decreased by MASH both in humans and mice.

To explore the mechanism underlying the lower hepatic CEBPA expression in MASH, 167 candidate transcription factors were predicted to bind to the *Cebpa* gene promoter (Supplemental Figure 1G and Supplemental Table 1). Differential gene expression (DGE) analyses to distinguish genes changed by MASH in mouse (GSE162276) and human livers (microarray E-MEXP-3291) were analyzed in both data sets. An increase of 795 mRNAs and a decrease of 382 mRNAs were associated with MASH (Supplemental Figure 1H), and among these genes, 14 MASH-increased and 3 MASH-decreased genes were overlapped with the 167 candidate transcriptional factors that were predicted to bind to the Cebpa gene promoter, as listed in Supplemental Table 1 (Supplemental Figure 1I). In particular, activating transcription factor 3 (ATF3), a transcriptional repressor, was previously shown to inhibit *Cebpa* transactivation in adipocytes (20). Thus, ATF3 was suspected to repress liver *Cebpa* transcription during MASH. Indeed, mouse ATF3 overexpression decreased *Cebpa* mRNA in primary hepatocytes (Supplemental Figure 1J). Another transcription factor, hes family bHLH transcription factor 1 (HES1), a transcriptional repressor known to be induced in hepatocytes and to promote fibrosis progression during MASH (21), was identified as a potential candidate that could repress liver *Cebpa* transcription during MASH. Hepatic *HES1* mRNA was positively correlated with liver fibrosis in humans (Supplemental Figure 1, K and L), and recombinant human HES1 overexpression repressed transactivation of the *CEBPA* gene (Supplemental Figure 1M). Further, human HES1 overexpression decreased *CEBPA* mRNA in primary human hepatocytes (Supplemental Figure 1N). Thus, ATF3 and HES1 upregulation by MASH may at least partially explain the MASH-induced decrease of CEBPA expression.

### Hepatocyte-specific CEBPA deficiency enhances MASH-associated fibrosis in mice.

**Figure 2:**
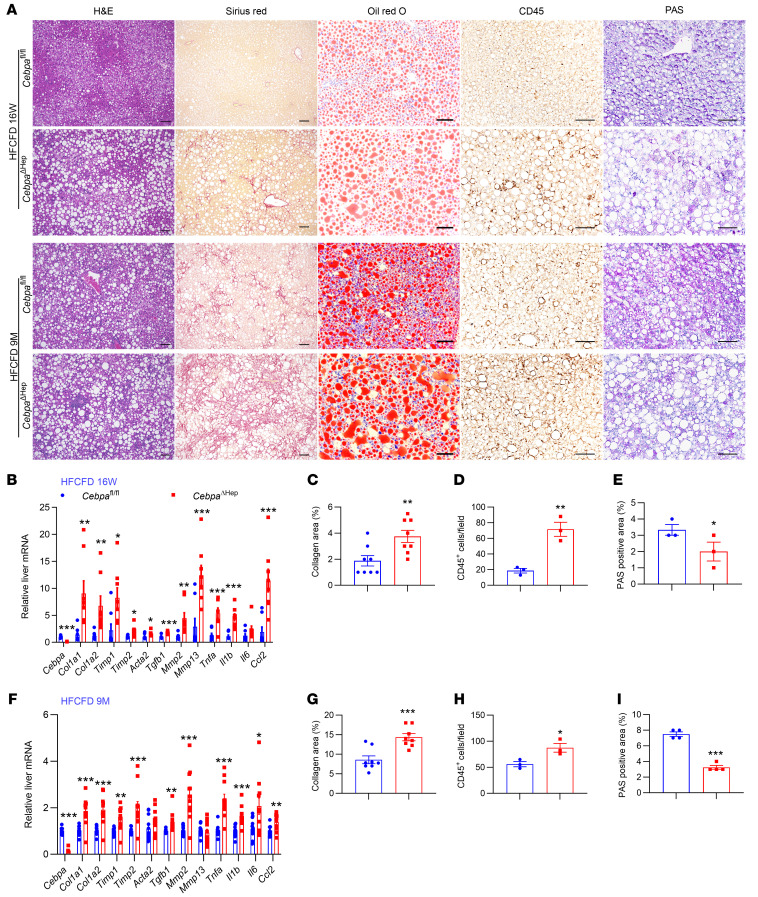
Constitutive hepatocyte CEBPA loss enhances MASH-associated liver fibrosis. (**A**) Representative histological staining. (**B**–**E**) Liver mRNAs in fibrosis and inflammation for mice fed a HFCFD for 16 weeks (**B**, *n* = 8), and the quantitation of liver Sirius red staining (**C**, *n* = 8), CD45 staining (**D**, *n* = 3) and PAS staining (**E**, *n* = 3) for mice. (**F**–**I**) Liver mRNAs in fibrosis and inflammation for mice fed a HFCFD for 9 months (**F**, *n* = 12 for *Cebpa*^fl/fl^ mice and *n* = 11 for *Cebpa*^ΔHep^ mice) and the quantitation of liver Sirius red staining (**G**, *n* = 8), CD45 staining (**H**, *n* = 3) and PAS staining (**I**, *n* = 4). Data represent mean ± SEM. *P < 0.05, **P < 0.01, ***P < 0.001 by 2-tailed unpaired student’s *t* test. Scale bars: 50 μm for Oil red O staining; 100 μm for others.

Next, a *Cebpa*^ΔHep^ mouse strain was generated to study the role of hepatocyte CEBPA in the progression of fibrosis associated with MASH (Supplemental Figure 2, A and B). Lower (approximately 50%) survival was noted in *Cebpa*^ΔHep^ mice compared with *Cebpa*^fl/fl^ littermates at 3 weeks of age in both females and males, which was likely due to decreased hepatic glycogen storage during the early developmental stage, similar to that found in global *Cebpa*-null mice (16). The survival rate of *Cebpa*^ΔHep^ mice was comparable to *Cebpa*^fl/fl^ mice after the mice were weaned at 3 weeks of age, and 10-week-old chow-fed *Cebpa*^ΔHep^ mice showed comparable body weights, liver weights, liver weight/body weight ratios, serum alanine transaminase (ALT) levels, triglycerides (TG), nonesterified fatty acids (NEFA), hepatic total cholesterol (TC), TG and NEFA, and liver histology, as well as mRNAs involved in hepatic fibrosis and inflammation accompanied by lower serum TC (Supplemental Figure 2, C–E). After HFCFD feeding for 16 weeks, *Cebpa*^ΔHep^ mice showed similar body weights and insulin sensitivity with the *Cebpa*^fl/fl^ mice, but developed increased liver weights, liver/body weight ratios, liver TG, serum ALT and terminal deoxynucleotidyl transferase dUTP nick end labeling (TUNEL) staining without changing liver TC, serum TG and NEFA, accompanied by lower serum TC (Supplemental Figure 2F). Notably, histological analyses by H&E staining and Oil red O staining revealed larger lipid droplets, while Sirius red staining revealed higher positive staining in livers of *Cebpa*^ΔHep^ mice (Figure 2A). mRNAs involved in hepatic fibrogenesis and inflammation were markedly increased in *Cebpa*^ΔHep^ mice (Figure 2B), accompanied by a quantitative increase in collagen deposition (Figure 2C), increased positive IHC staining of proinflammatory marker CD45 (Figure 2D), and reduced hepatic glycogen storage (Figure 2E). The mRNAs involved in hepatic lipogenesis, lipid transport, fatty acid β-oxidation, and bile acid signaling remained unchanged or decreased, except that hepatic *Smpdl3b* mRNA, encoding an enzyme involved in lipid drop formation, was significantly increased (Supplemental Figure 2G). The enhanced hepatic fibrosis and inflammation phenotype was consistently observed after an extended HFCFD feeding for as long as 9 months accompanied by enhanced IHC staining of CD45 and reduced hepatic glycogen storage (Figure 2, A and F–I). The body weights and liver weights were slightly but significantly increased, and liver weight ratios, hepatic lipids and liver TG slightly increased, while serum ALT and TC decreased, but other biochemical parameters remained similar between the 2 genotypes (Supplemental Figure 2H). Further, hepatic fibrogenesis signaling was significantly enhanced even in 9-month chow diet-fed *Cebpa*^ΔHep^ mice without significantly changing body weights, liver weights, and biochemical parameters, albeit no obvious increase of collagen deposition was detectable by Sirius red staining (Supplemental Figure 3, A–C and Supplemental Figure 2I). In chow-fed mice, hepatic glycogen storage was reduced in the 9-month chow-fed mice or short-term 2-week HFCFD-fed mice, albeit not in 10-week-old chow-fed mice (Supplemental Figure 3, D and E). Given that *CEBPA* mRNA levels were decreased by about 50% both in human patients and in mice, hepatocyte heterozygous CEBPA-KO (*Cebpa*^ΔHep,f/+^) mice were used to test whether a reduction of CEBPA affected the extent of liver fibrosis. After both 16-week and 30-week HFCFD feeding, *Cebpa*^ΔHep,f/+^ mice consistently developed increased liver fibrosis and inflammation (Supplemental Figure 3, F and G). Thus, even a 50% decrease in hepatocyte CEBPA enhanced MASH-associated liver fibrosis.

### Hepatocyte CEBPA deficiency at the progressive stage of MASH promotes liver fibrosis.

**Figure 3:**
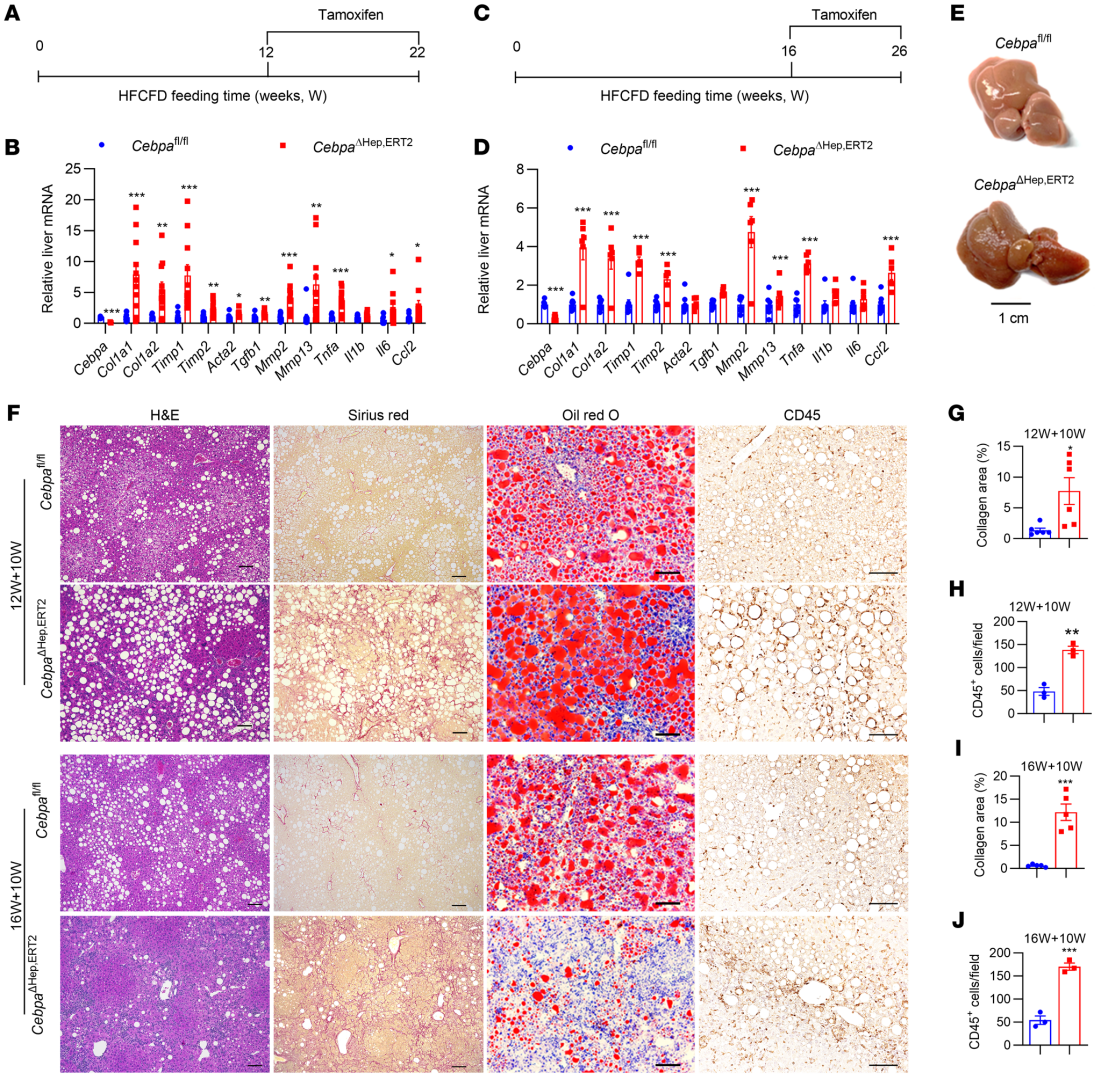
Hepatocyte CEBPA loss at the progressive stage of MASH exacerbates liver fibrosis. (**A**–**D**) Time scheme (**A** and **C**), liver mRNAs in fibrosis and inflammation of livers from 22-week HFCFD-fed mice dosed with tamoxifen for the last 10 weeks (12W+10W; **B**, *n* = 12 for *Cebpa*^fl/fl^ mice and *n* = 11 for *Cebpa*^ΔHep^ mice) or 26-week HFCFD-fed mice dosed with tamoxifen for the last 10 weeks (16W+10W; **D**, *n* = 8 for *Cebpa*^fl/fl^ mice and *n* = 6 for *Cebpa*^ΔHep,ERT2^ mice). (**E**) Liver pictures for mice treated as schemed in **C**. (**F**) Representative histological staining, quantitation of liver Sirius red staining (**G**, *n* = 6 for 12W+10W and **I**, *n* = 5 for 16W+10W) and CD45 staining (**H** and **J**, *n* = 3). Data represent mean ± SEM. *P < 0.05, **P < 0.01, ***P < 0.001, by 2-tailed unpaired student’s *t* test. Scale bar: 50 μm for Oil red O staining; 100 μm for others.

To avoid the potentially compensatory effect during early embryo development or growth and to check the function of hepatocyte CEBPA loss at the later stages of MASH, a tamoxifen-inducible *Cebpa*^ΔHep,ERT2^ mouse strain was generated to achieve temporal and spatial loss of hepatocyte CEBPA at the progressive stage of MASH. Post-adult tamoxifen dosing in chow diet-fed *Cebpa*^ΔHep,ERT2^ mice revealed a marked decrease in hepatic *Cebpa* mRNA and CEBPA protein with no significant change in liver histology (Supplemental Figure 4, A–C). Two sets of *Cebpa*^ΔHep,ERT2^ mice were subjected to experiments (Figure 3, A and C). The mice showed comparable body weights, while the liver weights, liver weight ratios, serum ALT, and TUNEL staining were increased in the first experiment and remained unchanged in the second experiment, between the 2 genotypes (Supplemental Figure 4, D and E). Hepatic and serum TC, TG, and NEFA were decreased or unchanged between 2 genotypes in all mice, with the exception of liver TG levels in the first experiment, which were increased in *Cebpa*^ΔHep,ERT2^ mice (Supplemental Figure 4, D and E). Similarly, changes of hepatic lipids among these 2 experiments, as assessed by histological analyses, were varied, showing increased or decreased lipids in *Cebpa*^ΔHep,ERT2^ mice (Figure 3F). However, both sets of *Cebpa*^ΔHep,ERT2^ mice consistently developed markedly enhanced liver fibrosis as revealed by increased hepatic inflammation- and fibrosis-related mRNAs (Figure 3, B and D), while the stiffness of livers was much higher in HFCFD-fed *Cebpa*^ΔHep,ERT2^ mice, indicating increased liver cirrhosis (Figure 3E). Both sets of *Cebpa*^ΔHep,ERT2^ mice developed enhanced Sirius red staining and CD45 IHC staining (Figure 3, F–J). Thus, loss of hepatocyte CEBPA after MASH development could further promote liver fibrosis and potentiate cirrhosis, which is not always accompanied by enhanced fatty liver.

### Hepatocyte CEBPA negatively regulates Spp1 transcription and osteopontin release during MASH.

**Figure 4:**
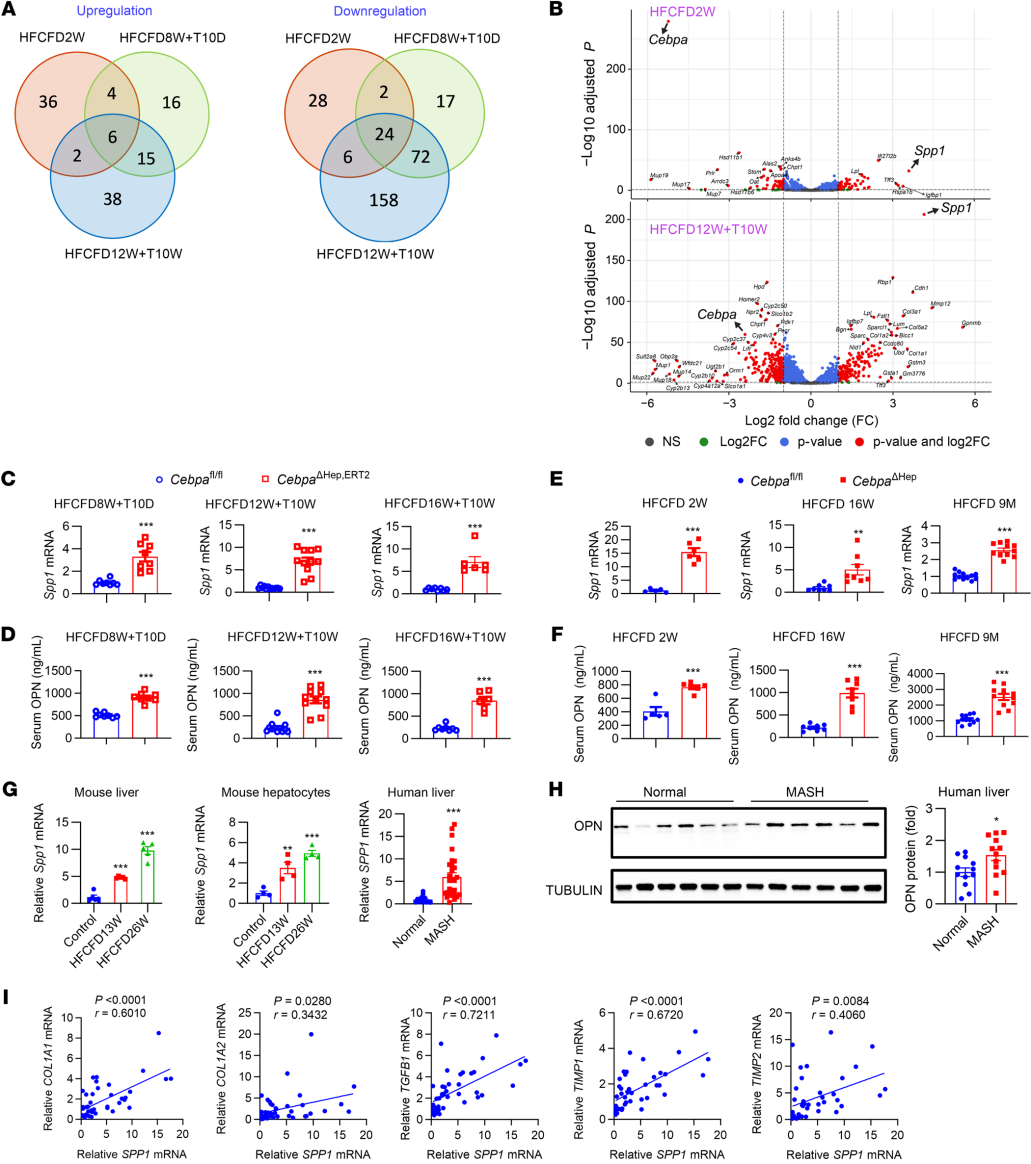
Hepatocyte CEBPA represses *Spp1* expression and OPN release in mice. (**A**) Venn diagram showing genes upregulated (left) or downregulated (right) by hepatocyte CEBPA knockout. (**B**) Volcano plots for RNA-Seq analyses of livers (HFCFD2W and HFCFD12W+T10W). Log_2_FC, FC > 2. *P*, *P*_adj_ < 0.05. *P* and log_2_FC, *P*_adj_ < 0.05 and FC > 2. (**C**–**F**) liver *Spp1* mRNA and Serum OPN in *Cebpa*^ΔHep,ERT2^ mice fed a HFCFD and dosed with tamoxifen as indicated (**C** and **D**, *n* = 6–12) and *Cebpa*^ΔHep^ mice fed a HFCFD for 2 weeks (*n* = 5 or 6), 16 weeks (*n* = 8), and 9 months (*n* = 11 or 12). T10D or T10W, tamoxifen for the last 10 days or 10 weeks. (**G**) *Spp1* mRNA in the livers (*n* = 5), primary hepatocytes (*n* = 4) from C57BL/6N mice fed a 13-week HFCFD or 26-week HFCFD with statistics calculated by 1-way ANOVA with Dunnett’s multiple-comparisons test and human livers (*n* = 13 or 28). (**H**) Representative OPN protein in human livers and quantitation (*n* = 12). (**I**) Correlation analyses of fibrosis gene mRNAs with *SPP1* mRNA in human livers by nonparametric Pearson’s test. Data represent mean ± SEM. *P < 0.05, **P < 0.01, ***P < 0.001 by 2-tailed unpaired student’s *t* test unless otherwise stated.

To clarify the mechanisms underlying the above phenotypes, RNA-Seq of liver mRNAs were used in HFCFD-fed mice either already showing a phenotype or prior to the occurrence of the phenotype. The DGE profiles between the 2 genotypes among 3 experiments were analyzed by Venn diagram, Volcano plots, and heatmaps, identifying 6 mRNAs that were consistently increased and 24 mRNAs that were decreased after hepatocyte-specific CEBPA deficiency (Figure 4, A and B and Supplemental Figure 5A). *Spp1* encoding the profibrogenic protein osteopontin (OPN), ranked among the top-upregulated mRNAs (Figure 4B and Supplemental Figure 5A). RNA-Seq was also performed in CEBPA-knockout or CEBPA-overexpressing primary hepatocytes. Of the top-changed genes in hepatocytes, *Spp1* was consistently found to rank among the top-changed mRNAs that were negatively regulated by hepatocyte CEBPA as a potential mechanistic hit (Supplemental Figure 5B). Serum OPN and hepatic *Spp1* mRNA were increased in all examined experimental sets of HFCFD-fed *Cebpa*^ΔHep,ERT2^ mice (Figure 4, C and D) and *Cebpa*^ΔHep^ mice (Figure 4, E and F) during both the early and late stages of MASH, as was the protein level in the livers from 2-week HFCFD-fed *Cebpa*^ΔHep^ mice (Supplemental Figure 5C). Serum OPN (Supplemental Figure 5, D and E) and hepatic *Spp1* mRNA (Supplemental Figure 5, F and G) were increased even in chow-fed *Cebpa*^ΔHep^ mice. Further, human liver *CEBPA* mRNA was negatively correlated with *SPP1* mRNA (Supplemental Figure 5H, TCGA database). Thus, hepatocyte CEBPA repressed hepatic *Spp1* expression and OPN secretion.

The relevance of *SPP1* expression with liver fibrosis was further examined in humans and mice. Human *SPP1* mRNA was increased in MASH livers, but not in simple steatotic livers, and strongly correlated with fibrosis markers (Supplemental Figure 5, I–N, microarray E-MEXP-3291). Hepatic *Spp1* mRNA was increased in both the livers and primary hepatocytes of HFCFD-fed mice as well as in human livers (Figure 4G). Liver OPN protein was increased by MASH in human livers (Figure 4H), and liver *SPP1* mRNA positively correlated with liver fibrosis markers (Figure 4I). In line with the fibrosis-promoting function of OPN, hepatic fibrosis signaling was a top-changed pathway in HFCFD-fed hepatocyte CEBPA-deficient mice, as revealed by Ingenuity Pathways Analysis (IPA) analysis (Supplemental Figure 5O). Thus, hepatic *SPP1* expression strongly tracks with liver fibrosis both in human and in hepatocyte CEBPA-deficient mice during MASH progression.

### CEBPA negatively modulates Spp1 and OPN release in hepatocytes.

**Figure 5:**
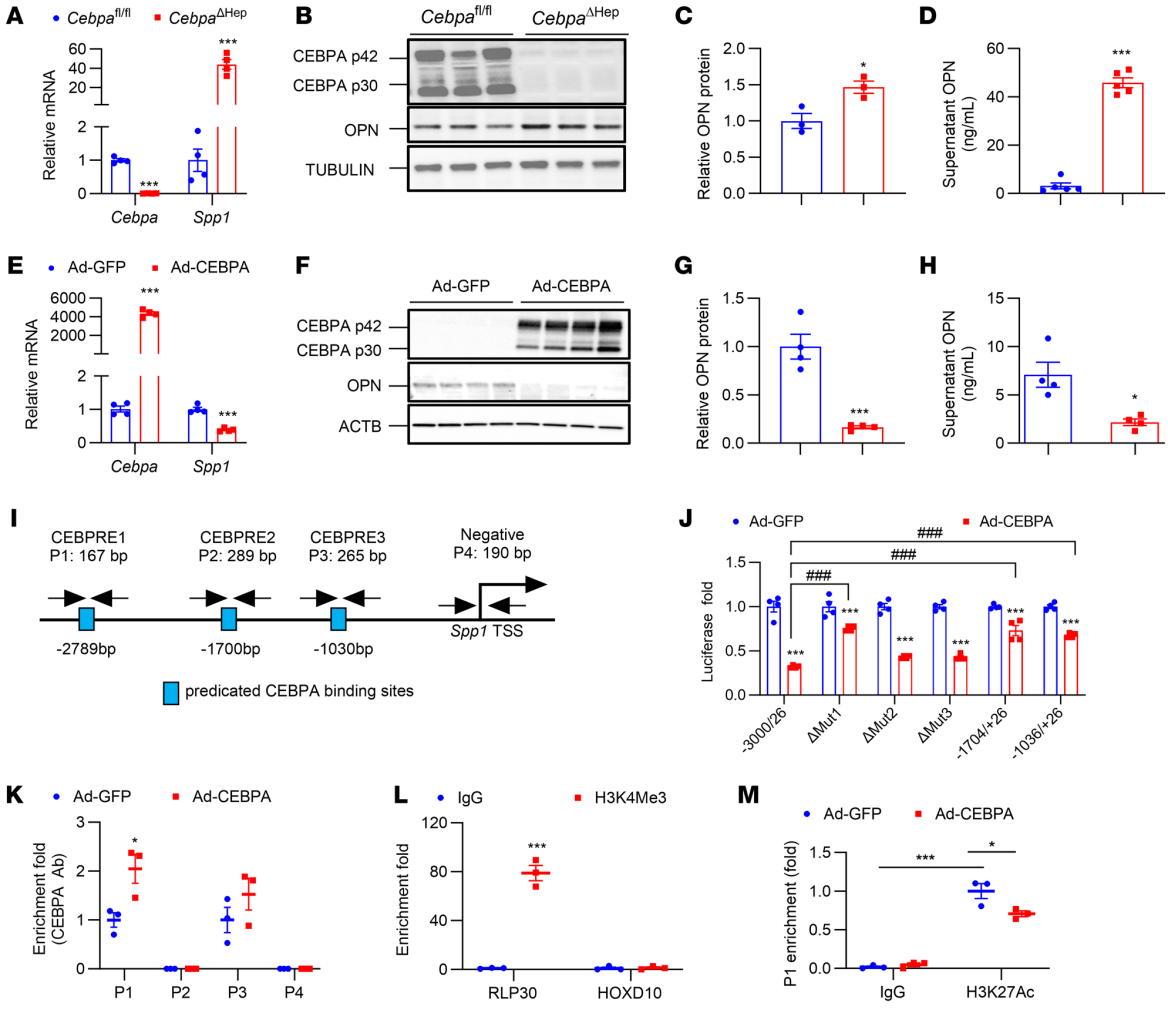
CEBPA represses *Spp1* expression and OPN release in primary hepatocytes in vitro. (**A**–**D**) *Cebpa* and *Spp1* mRNAs (**A**, *n* = 4), OPN protein (**B** and **C**, *n* = 3), and supernatant OPN (**D**, *n* = 5) of primary hepatocytes from chow-fed mice. (**E**–**H**) *Cebpa* and *Spp1* mRNAs (**E**, *n* = 4), OPN protein (**F**–**G**, *n* = 4), and supernatant OPN (**H**, *n* = 4) in 48 hour Ad-GFP or Ad-CEBPA-treated primary WT hepatocytes. (**I** and **J**) Schematic diagram of the mouse *Spp1* promoter illustrating the CEBPREs (**I**) and luciferase reporter assays (**J**, *n* = 4). (**K** and **L**) ChIP assay, relative CEBPA enrichment on CEBPRE1-4 (P1–P4) (**K**, *n* = 3) or H3K4Me3 enrichment on RLP30/HOXD10 (**L**, *n* = 3). (**M**) ChIP assay, relative H3K27Ac enrichment on CEBPRE1, *n* = 3. Data represent mean ± SEM. *P <0.05, **P < 0.01, ***P < 0.001, and ^###^*P* < 0.001 compared with each control group or as indicated by 2-way ANOVA with Šidák’s multiple-comparisons test for **J** and **M** or by 2-tailed unpaired student’s *t* test for others.

OPN is highly expressed in cholangiocytes, followed by Kupffer cells, hepatocytes, sinusoidal endothelial cells, and hepatic stellate cells (HSCs) in a healthy liver (22). Since *Spp1* was previously found to be induced by hepatic Notch signaling (21), the question arises whether hepatocyte CEBPA deficiency induced *Spp1* expression was via the Notch signaling pathway. Analyses of Notch signaling in primary mouse hepatocytes isolated from 10-week-old chow-fed mice and in the livers from 2-week HFCFD-fed mice and 10-week-old chow-fed mice showed that *Spp1* mRNA was induced after hepatocyte CEBPA knockout in the absence of any changes in Notch signaling, indicating that regulation of *Spp1* by CEBPA was not due to the Notch signaling pathway (Supplemental Figure 6, A–C). Next, experiments were performed to examine the mechanism by which hepatocyte CEBPA directly modulates *Spp1* expression in a cell-autonomous manner. *Spp1* mRNA was about 40 times higher in primary hepatocytes from *Cebpa*^ΔHep^ mice (Figure 5A), while OPN protein was significantly increased, but to a lesser extent (Figure 5, B and C), possibly due to the secretory property of OPN. Indeed, the primary hepatocytes from *Cebpa*^ΔHep^ mice secreted over 40 ng/mL of OPN to the supernatant after 24 hour culturing in vitro, which was much more than that found in *Cebpa*^fl/fl^ mice (Figure 5D). In contrast, CEBPA overexpression markedly decreased *Spp1* mRNA levels (Figure 5E) and OPN protein (Figure 5, F and G) in primary hepatocytes and reduced the supernatant OPN levels (Figure 5H).

Three putative sites, predicted to be CEBP regulatory elements (CEBPRE), were located within 3 kb upstream of the *Spp1* transcription start site, with CEBPRE1 (TGTCGCAATGGG), CEBPRE2 (TTTTACAACGTT) and CEBPRE3 (TTTTGCAATGCT) showing 91%, 92%, and 95% homology with a typical CEBPRE consensus sequence, as described previously (23) (Figure 5I). Luciferase activity of the *Spp1* luciferase reporter (*Spp1*-luc) (–3000/+26) construct was substantially inhibited by Ad-CEBPA, while the inhibitory effect of Ad-CEBPA on the *Spp1*-luc (–3000/+26) activity was significantly rescued by site mutation on CEBPRE1, but not by site mutation on either CEBPRE2 or CEBPRE3 (Figure 5J). Reporter assays of the *Spp1*-luc (–1704/+26) revealed that deleting the CEBPRE1-containing promoter region significantly rescued the inhibitory effect of Ad-CEBPA on *Spp1*-luc activity, while luciferase activity of the –1036/+26 mutant with deleted CEBPRE1 and CEBPRE2 sites failed to be further enhanced compared with the –1704/+26 mutant (Figure 5J). Analyses of the CEBPA ChIP-Seq database (GSE65167) and H3K27ac ChIP-Seq database (GSE60430) showed that both CEBPA and H3K27ac had binding peaks at the position of CEBPRE1 located upstream of the *Spp1* promoter (Supplemental Figure 6D). ChIP assays revealed an enhanced enrichment of CEBPA on CEBPRE1 by Ad-CEBPA (Figure 5K), with markedly more enrichment of H3K4Me3 than IgG on RLP30 but not on HOXD10, which served as a positive and negative controls for the ChIP assay, respectively (Figure 5L). Further, the enrichment of H3K27ac, an active enhancer of epigenetic modification, on the CEBPRE1 element of the *Spp1* promoter, was markedly reduced by Ad-CEBPA (Figure 5M). Trichostatin A, an histone deacetylase inhibitor that increases H3K27ac (24), significantly induced *Spp1* mRNA levels in hepatocytes isolated from WT *Cebpa*^fl/fl^ mice and restored the Ad-CEBPA-downregulated *Spp1* mRNA (Supplemental Figure 6E), while trichostatin A failed to further enhance *Spp1* mRNA levels in hepatocytes isolated from *Cebpa*^ΔHep^ mice (Supplemental Figure 6F). Thus, CEBPA overexpression repressed *Spp1* transactivation by binding to the CEBPRE1, which was at least partially through blocking the transcriptional enhancer H3K27ac binding to the *Spp1* promoter.

### Hepatocyte CEBPA-SPP1 axis contributes to HSC activation and liver fibrosis in MASH.

**Figure 6:**
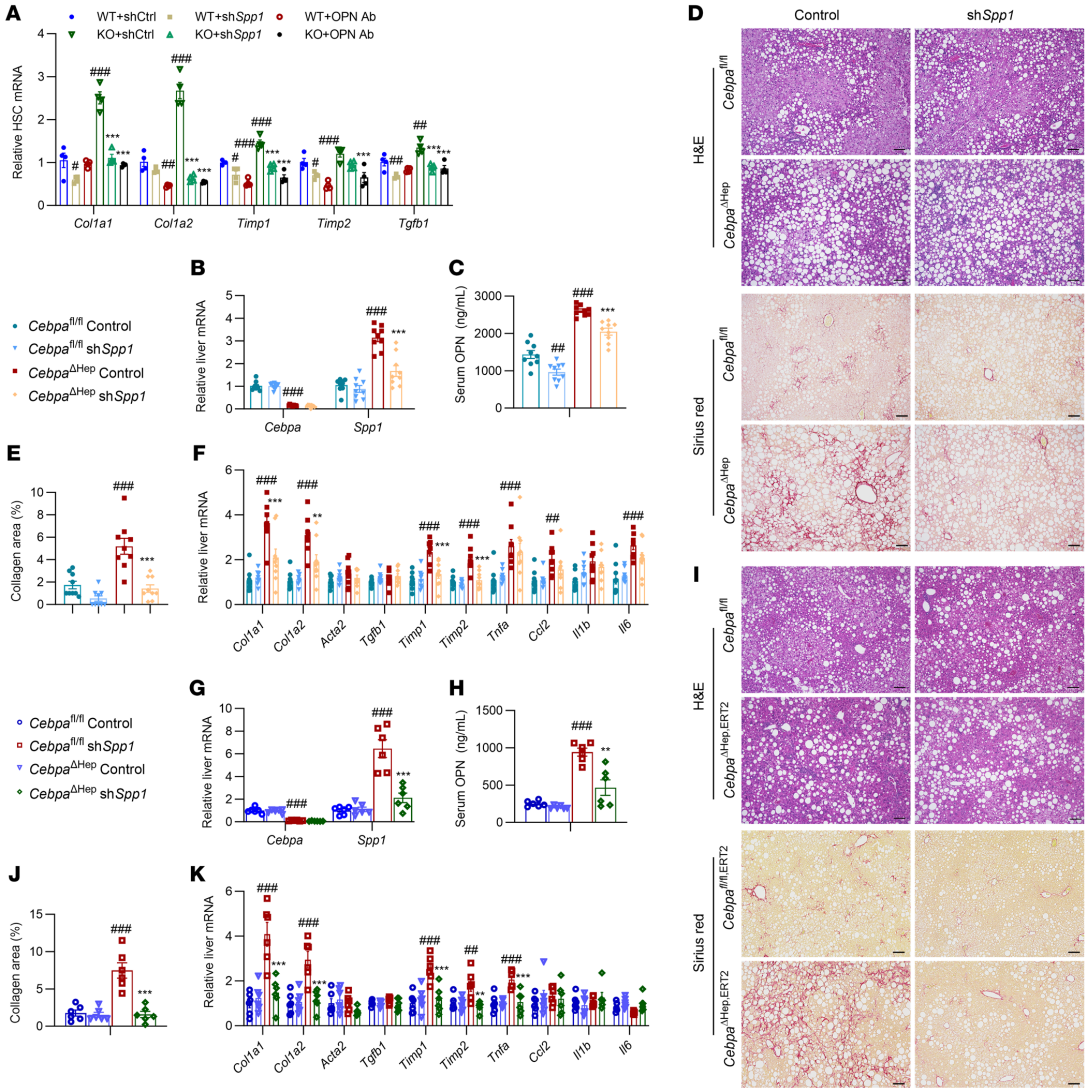
Hepatocyte CEBPA deficiency–induced *Spp1* expression and OPN release activates HSCs to promote MASH fibrosis. (**A**) Fibrosis gene mRNAs of primary HSCs treated with supernatants from hepatocytes or with mouse OPN antibody (OPN Ab), *n* = 4. shCtrl, control scrambled shRNA; sh*Spp1*, *Spp1* shRNA. ^#^*P* < 0.05, ^##^*P* < 0.01, ^###^*P* < 0.001 compared with WT+shCtrl, while *P < 0.05, **P < 0.01, ***P < 0.001 compared with KO+shCtrl. (**B**–**F**) Liver *Cebpa* and *Spp1* mRNAs (**B**, *n* = 9), serum OPN (**C**, *n* = 9), representative histological staining (**D**), quantitation of Sirius red staining (**E**, *n* = 9), and liver mRNAs in fibrosis and inflammation (**F**, *n* = 9) in 16-week HFCFD-fed *Cebpa*^ΔHep^ mice treated with AAV8-control scrambled shRNA (control) or AAV8-Spp1 shRNA (sh*Spp1*). (**G**–**K**) Liver *Cebpa* and *Spp1* mRNAs (**G**, *n* = 6), serum OPN (**H**, *n* = 6), representative histological staining (**I**), quantitation of Sirius red staining (**J**, *n* = 6) and liver mRNAs in fibrosis and inflammation (**K**, *n* = 6) in *Cebpa*^ΔHep,ERT2^ mice fed a HFCFD for 24 weeks and treated with tamoxifen for the last 12 weeks with AAV8 dosing at 1 week prior to tamoxifen dosing. Data represent mean ± SEM. *Cebpa*^fl/fl^ Control or sh*Spp1*, *Cebpa*^fl/fl^ mice treated with AAV8-control scrambled shRNA or AAV8-sh*Spp1*. *Cebpa*^ΔHep^ or *Cebpa*^ΔHep,ERT2^ Control or sh*Spp1*, *Cebpa*^ΔHep^ or *Cebpa*^ΔHep,ERT2^ mice treated with AAV8-control scrambled shRNA or AAV8-sh*Spp1*. ^#^*P* < 0.05, ^##^*P* < 0.01, ^###^*P* < 0.001 compared with *Cebpa*^fl/fl^ Control, while *P < 0.05, **P < 0.01, ***P < 0.001 compared with *Cebpa*^ΔHep^ Control or *Cebpa*^ΔHep,ERT2^ Control by 2-way ANOVA with Šidák’s multiple-comparisons test. Scale bars: 100 μm.

To determine whether increased hepatocyte *Spp1* mRNA and OPN secretion induced by hepatocyte-specific CEBPA knockout contributes to enhanced HSC activation, hepatocytes from *Cebpa*^ΔHep^ or *Cebpa*^fl/fl^ mice were transfected with control or *Spp1* shRNA in the presence of palmitic acid. The *Spp1* shRNA markedly decreased OPN in the hepatocyte culture medium (Supplemental Figure 7A). The expression of fibrogenic gene mRNAs in primary mouse HSCs was increased by the culture medium from *Cebpa*^ΔHep^ hepatocytes compared to that from *Cebpa*^fl/fl^ hepatocytes, while this phenotype was rescued by *Spp1* shRNA or OPN-neutralizing antibody (Figure 6A).

Next, scAAV8-U6-sh*Spp1* was generated to knockdown hepatocyte *Spp1* in vivo. In C57BL/6N mice, scAAV8-U6-sh*Spp1* markedly decreased *Spp1* mRNA in primary hepatocytes, but not in nonparenchymal cells (Supplemental Figure 7B). In 16-week HFCFD-fed *Cebpa*^ΔHep^ mice, scAAV8-U6-sh*Spp1* did not affect the body weights, liver weights, and biochemical parameters (Supplemental Figure 7C), but significantly reduced hepatic *Spp1* mRNA, serum OPN, hepatocyte CEBPA knockout-potentiated liver fibrogenic gene expression and collagen deposition (Figure 6, B–F). Similarly, 24-week HFCFD-fed *Cebpa*^ΔHep,ERT2^ mice treated with tamoxifen for the last 12 weeks, developed enhanced liver fibrosis while scAAV8-U6-sh*Spp1* rescued the hepatocyte CEBPA deficiency-enhanced liver fibrosis (Figure 6, G–K) without changing other biochemical parameters (Supplemental Figure 7D). Further, in the presence of scAAV8-U6-sh*Spp1*, hepatocyte CEBPA deficiency failed to significantly enhance the HFCFD-induced liver fibrosis (Figure 6, E and J). All these data indicate that induction of *Spp1* gene expression by hepatocyte CEBPA deficiency predominantly contributes to the enhanced MASH-associated liver fibrosis.

To explore whether the induction of *Spp1* gene expression and OPN release were caused by signaling in macrophages, macrophages were depleted using clodronate for 2 weeks, revealing that hepatocyte CEBPA deficiency still sharply induced liver *Spp1* mRNA expression and OPN release; *Adgre1* mRNA, encoding a macrophage marker, was almost completely lost in clodronate-treated mice, indicating the efficient depletion of macrophages in the livers (Supplemental Figure 7E). Further, when macrophages were depleted by 4-week clodronate dosing, the effect of scAAV8-U6-sh*Spp1* in rescuing hepatocyte CEBPA deficiency–enhanced liver fibrosis was found comparable to that in control vehicle-dosed mice (Supplemental Figure 7F). In line with this result, single-cell RNA-Seq (scRNA-Seq) analyses of 2-week HFCFD-fed mice showed that *Spp1* was mostly induced in hepatocytes (Supplemental Figure 8, A–F), which was further confirmed by quantitative PCR (qPCR) analyses of mRNAs in primary hepatocytes and enriched nonparenchymal cells isolated from 2-week HFCFD-fed mice (Supplemental Figure 8, G and H), while immunofluorescence staining consistently demonstrated that OPN was mainly induced in hepatocytes, but not in macrophages, in the livers of 2-week HFCFD-fed *Cebpa*^ΔHep^ mice (Supplemental Figure 8, I and J). These data support the view that macrophages do not significantly contribute to the CEBPA-OPN modulation on MASH fibrosis.

### Hepatocyte CEBPA/SPP1 axis modulates CCl_4_-induced liver fibrosis.

**Figure 7:**
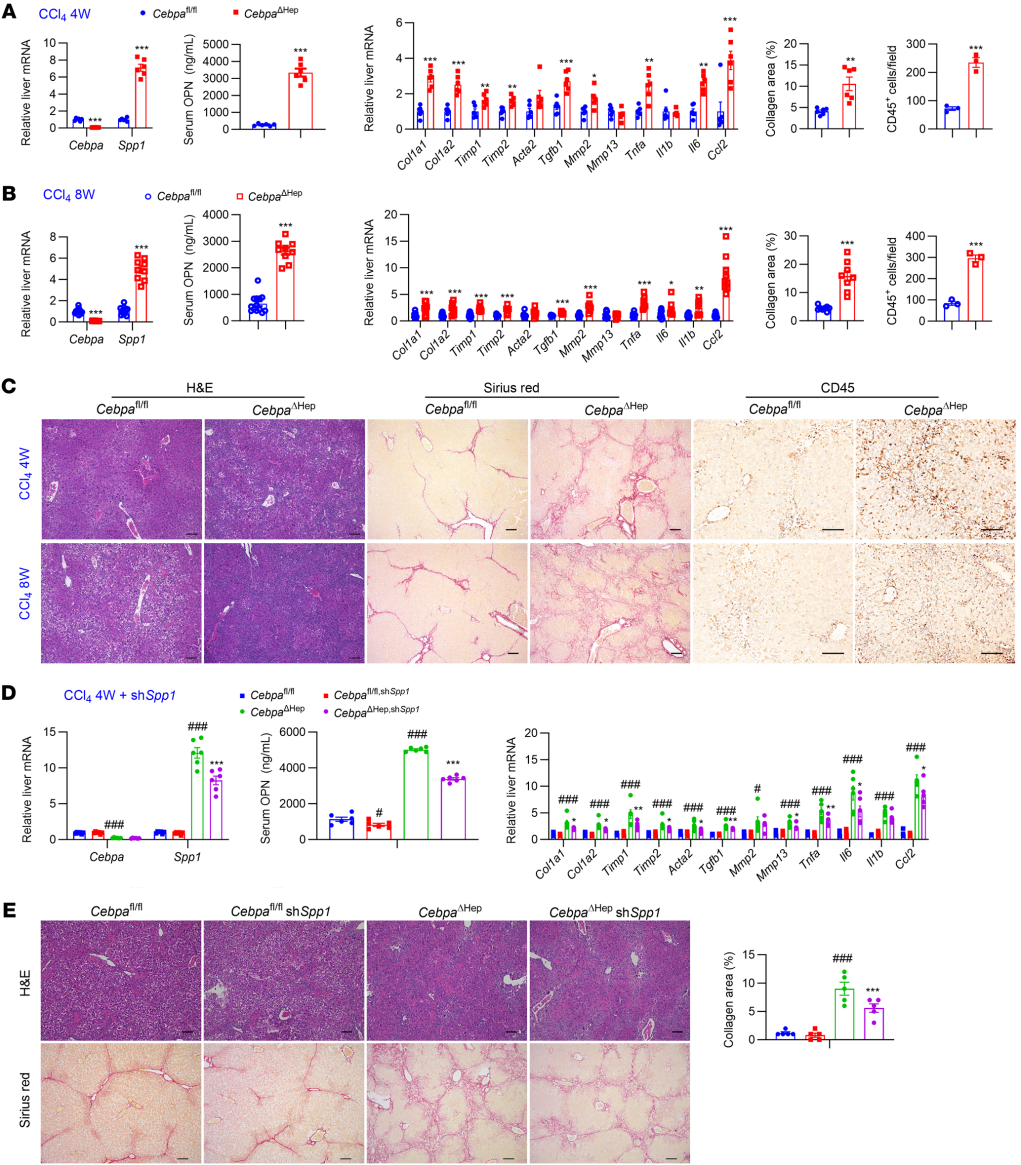
Hepatocyte-specific CEBPA knockout enhances CCl_4_-induced liver fibrosis via *Spp1* induction. (**A**) Liver *Cebpa* and *Spp1* mRNAs (*n* = 6), serum OPN (*n* = 6), liver mRNAs in fibrosis and inflammation (*n* = 6), and quantitation of Sirius red staining (*n* = 6) and CD45 staining (*n* = 3) for 4-week CCl_4_-treated mice. (**B**) Liver *Cebpa* and *Spp1* mRNAs (*n* = 9-13), serum OPN (*n* = 9-13), liver mRNAs in fibrosis and inflammation (*n* = 9-13), and quantitation of Sirius red staining (*n* = 8) and CD45 staining (*n* = 3) for 8-week CCl_4_-treated mice. (**C**) Representative histological staining for the livers from 4-week CCl_4_-treated mice and 8-week CCl_4_-treated mice. (**D**–**E**) Liver *Cebpa* and *Spp1* mRNAs, serum OPN, liver mRNAs in fibrosis and inflammation (**D**, *n* = 6), representative histological staining and quantitation of Sirius red staining (**E**, *n* = 5) in 4-week CCl_4_-treated mice dosed with AAV8-control scrambled shRNA (control) or AAV8-Spp1 shRNA (sh*Spp1*). Data represent mean ± SEM. ^#^*P* < 0.05, ^##^*P* < 0.01, ^###^*P* < 0.001 compared with *Cebpa*^fl/fl^ group, while *P < 0.05, **P < 0.01, ***P < 0.001 compared with the *Cebpa*^ΔHep^ group by 2-tailed unpaired student’s *t* test for **A**–**C** or by 2-way ANOVA with Šidák’s multiple-comparisons test for **D**–**E**. Scale bars: 100 μm.

The role of CEBPA and SPP1 in carbon tetrachloride-induced (CCl_4_-induced) fibrosis in the absence of MASH was next tested. After CCl_4_ dosing for 4 weeks and 8 weeks, *Cebpa*^ΔHep^ mice had higher hepatic *Spp1* mRNA, serum OPN, liver fibrosis, and hepatic CD45 staining (Figure 7, A–C). The 4-week CCl_4_-treated *Cebpa*^ΔHep^ mice showed lower serum TC and liver TC and TG, without changes in other biochemical parameters (Supplemental Figure 9A). Similarly, after CCl_4_ dosing for 8 weeks, *Cebpa*^ΔHep^ mice showed lower liver TG, NEFA, serum ALT, and TC without significant changes in other biochemical parameters (Supplemental Figure 9B). Further, scAAV8-U6-sh*Spp1* reduced liver *Spp1* mRNA, decreased serum OPN, and rescued the enhanced liver fibrosis and inflammation in *Cebpa*^ΔHep^ mice (Figure 7, D and E), while not significantly changing other biochemical parameters (Supplemental Figure 9C). Thus, hepatocyte CEBPA restricted CCl_4_-induced hepatic fibrosis through reducing *Spp1* expression.

### AAV-based gene therapy targeting hepatocyte CEBPA ameliorates MASH and fibrosis.

**Figure 8:**
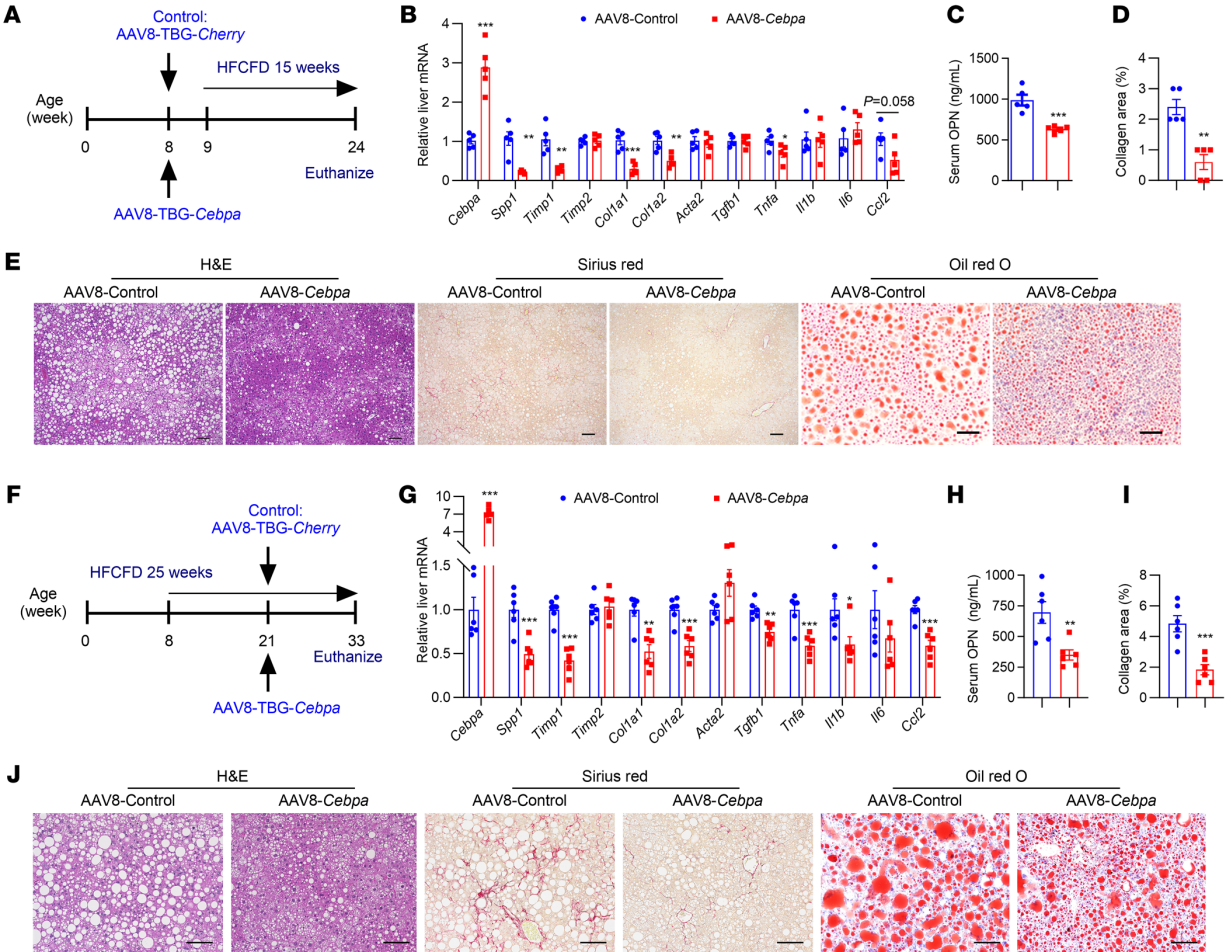
AAV8-TBG-*Cebpa* reduces liver fibrosis in HFCFD-fed mice. (**A**–**E**) Preventive dosing scheme (**A**), liver mRNAs in fibrosis and inflammation (**B**), serum OPN (**C**), quantitation of Sirius red staining (**D**) and representative histological staining (**E**), scale bar: 100 μm for H&E and Sirius red staining; 50 μm for Oil red O staining; *n* = 5. (**F**–**J**) Therapeutic dosing scheme (**F**), liver mRNAs in fibrosis and inflammation (**G**), serum OPN (**H**), quantitation of Sirius red staining (**I**), and representative histological staining (**J**). Scale bar: 100 μm; *n* = 6. Data represent mean ± SEM. *P < 0.05, **P < 0.01, ***P < 0.001, by 2-tailed unpaired student’s *t* test.

The effect of hepatocyte-specific CEBPA overexpression in the treatment of MASH-associated fibrosis was next examined. By taking advantage of liver tropism of AAV8 and the hepatocyte-specific thyroxine-binding protein globulin (*Tbg*) promoter, AAV8-TBG-*Cebpa* was generated to express CEBPA specifically in hepatocytes. In the preventive treatment scheme, AAV8-TBG-*Cebpa* increased hepatic CEBPA expression and reduced mRNAs involved in fibrosis and inflammation, hepatic *Spp1* mRNA, serum OPN, collagen deposition, hepatic TC, TG, NEFA, and serum ALT, with no changes in serum TC, TG and NEFA (Figure 8, A–E and Supplemental Figure 10, A–D). In the therapeutic treatment scheme, 25-week HFCFD-fed C57BL/6N mice were treated with AAV8-TBG-*Cebpa* for the last 12 weeks. AAV8-TBG-*Cebpa* increased hepatic *Cebpa* mRNA and CEBPA protein, while decreased hepatic *Spp1* mRNA, serum OPN, hepatic lipid accumulation, and liver fibrosis, accompanied by a reduction in liver TG, serum ALT, and NEFA without changing other parameters (Figure 8, F–J and Supplemental Figure 10, E–H). As early as 3 weeks after AAV8-TBG-*Cebpa* dosing in HFCFD-fed C57BL/6N mice, hepatic *Cebpa* mRNA was increased, while hepatic *Spp1* mRNA and serum OPN were decreased, in the absence of obvious phenotypic changes of biochemical endpoints and liver fibrosis (Supplemental Figure 10, I–P), supporting the view that CEBPA overexpression–mediated *Spp1* downregulation could be an early causal factor for decreasing liver fibrosis.

## Discussion

In this study, CEBPA was found to restrict both MASH and chemically induced fibrosis by using 2 hepatocyte-specific CEBPA-deficient mouse lines and AAV8-TBG-*Cebpa*. Mechanistically, hepatocyte CEBPA bound to CEBPRE1 may competitively reduce the H3K27ac engagement at the *Spp1* promoter. This reduced engagement repressed *Spp1* to decrease hepatocyte-derived OPN synthesis and release into the hepatic microenvironment and HSC activation.

OPN hyperactivation could promote HSC activation and liver fibrogenesis (22). In earlier studies, serum OPN levels were associated with liver fibrosis in patients with MASH and correlated with liver stiffness in patients with cirrhosis, while OPN-neutralizing antibody reduced MASH in mice, suggesting that serum OPN levels serve as noninvasive biomarkers for liver fibrosis progression (25–27). Herein, by using biopsies from human livers, a strong positive correlation of hepatic *SPP1* mRNA with liver fibrosis markers was further found. Macrophages are a source of OPN production (22). However, the MASH-increased liver *Spp1* mRNA is mainly derived from the marked *Spp1* induction in hepatocytes and not Kupffer cells. Another earlier study demonstrated that hepatocyte-derived OPN induction promoted liver fibrosis (21), which is in line with the current study that consistently supports a fibrosis-promoting role for hepatocyte-derived OPN. However, a recent study demonstrated that macrophage-derived OPN protected against MASH progression in contrast to its well-known typical fibrosis-promoting function (28). Thus, while the effects of OPN produced in macrophages and hepatocytes on MASH have been shown to be different, the current work supports the view that increasing hepatocyte CEBPA to achieve the hepatocyte-specific restriction of MASH-induced Spp1 transactivation could be a promising strategy for fibrosis treatment. Here, hepatocyte CEBPA is demonstrated to impede hepatic fibrogenesis as a direct transcriptional repressor of hepatocyte *Spp1*.

Notably, supernatant OPN levels in the primary hepatocyte culture medium are high and could be markedly increased by hepatocyte CEBPA knockout, revealing that hepatocytes substantially contribute to OPN secretion and that CEBPA is a strong negative modulator of hepatocyte OPN release. The contribution of hepatocyte CEBPA to regulating the circulating OPN levels is further supported by increased serum OPN in hepatocyte-specific CEBPA-deficient mice under both basic and hepatic insult-challenging conditions. The causal contribution of hepatocyte CEBPA deficiency–induced *Spp1* transactivation and OPN release to the phenotype is supported by the rescue experiments using *Spp1* shRNA or OPN antibody in vitro as well as using AAV8-U6-sh*Spp1* in vivo, despite the fact that other factors that may contribute could not be totally excluded. However, decreasing *Spp1* mRNA levels by AAV8-U6-sh*Spp1* fails to lower hepatocyte CEBPA knockout-potentiated fatty liver, liver inflammation, and serum ALT during MASH, suggesting that it is less likely that hepatocyte-derived OPN promotes liver fibrosis dependent on its modulation of hepatic steatosis, inflammation, and hepatocyte death during MASH. However, it is still possible that hepatocyte CEBPA deficiency increases hepatocellular cell death during the early stages of MASH, which may contribute to the enhanced liver fibrosis phenotype. Hepatocyte CEBPA loss increases the infiltration of proinflammatory CD45^+^ cells in the presence of HFCFD challenge or CCl_4_ stimuli, but how the crosstalk between hepatocyte CEBPA and immune cells mediates the phenotype, either dependent or independent of OPN, still requires further investigation. AAV8-U6-sh*Spp1* reduces some proinflammatory markers when rescuing CCl_4_-induced liver fibrosis, which represents a more proinflammatory condition than MASH, indicating potential pleiotropic effects of OPN among different experimental contexts.

In the present study, HES1 was identified as a transcription repressor of CEBPA. In line with the CEBPA/OPN axis modulation on liver fibrosis, hepatocyte HES1 induction–enhanced liver fibrosis similarly induces hepatocyte-derived OPN release (21). However, hepatocyte HES1 induction increases *Spp1* expression at least partially via inducing *Sox9* expression (21). In contrast, hepatocyte CEBPA loss is found not to change *Sox9* expression in the present study, suggesting that HES1 and CEBPA work on different signaling pathways to regulate *Spp1* expression. Investigation is still warranted regarding whether and how HES1 modulation of *Spp1* expression in vivo depends on its modulation of CEBPA expression in hepatocytes. It should be noted that ATF3 upregulation is found to repress *Cebpa* mRNA expression in primary hepatocytes in the present study, which is consistent with the earlier work demonstrating the repression of CEBPA transactivation by ATF3 in adipocytes (20). However, as to whether ATF3 could be a therapeutic target upstream of the CEBPA-fibrosis axis, 2 earlier publications demonstrated that ATF3 induction actually protected against MASH progression (29, 30). This protection is not consistent with the fibrosis-promoting effect due to CEBPA repression by ATF3 induction, suggesting that ATF3 induction during MASH as an adaptive response could only partially serve to repress CEBPA expression, but that it is not a causal factor to MASH progression. The factors that cause hepatocyte CEBPA downregulation during MASH progression could be more complicated, and the present study could not rule out other factors that may contribute to CEBPA downregulation during MASH.

The pathophysiological role of CEBPA in the liver may be complicated and pleiotropic. Hepatic CEBPA was suggested to promote hepatic lipogenesis (18, 19), while hepatocyte CEBPA knockout potentiated fatty liver in some currently examined experimental MASH conditions, raising new concerns regarding the lipid-lowering role of hepatocyte CEBPA. Hepatic CEBPA knockout may increase hepatic lipids by upregulating lipogenesis genes, such as *Smpdl3b,* or inhibiting fatty acid β-oxidation, while, in contrast, hepatic lipids may be reduced due to the deteriorated liver function caused by hepatocyte CEBPA loss in the progressive stage of fibrosis (31). CEBPA also controls self-renewal of fetal liver and adult hematopoietic cells and restricts hepatic proliferation (32, 33). Thus, various factors modulated by hepatic CEBPA may work together to determine their net effects on liver weight and hepatic lipid accumulation. However, given that hepatocyte CEBPA knockout, which enhances liver fibrosis, is not always accompanied by exacerbated fatty liver during MASH and that it even enhances CCl_4_-induced liver fibrosis — accompanied by decreased hepatic lipids — it is less likely that hepatocyte CEBPA knockout promotes liver fibrosis depending on its effect on increasing hepatic lipids. Additionally, CEBPA positively modulates hepatic glycogen storage (15, 16), while lower hepatic glycogen storage was consistently found in HFCFD-fed *Cebpa*^ΔHep^ mice in the present study. While progressive abnormal glycogen accumulation is thought to promote liver fibrosis and cirrhosis in glycogen storage disease type III (34, 35), how hepatic normal glycogen levels modulate liver fibrosis remains unclear. The causal relationship among hepatic glycogen storage, liver fibrosis progression, and SPP1 modulation requires further investigation.

In summary, the current findings reveal a key role of hepatocyte CEBPA in restricting the liver fibrosis progression and support the application of AAV-based therapies for the treatment of MASH-associated liver fibrosis. Studies regarding the cell-specific roles of CEBPA in hepatocytes or other cells in liver diseases represent a promising research field to guide the discovery of CEBPA modulation–based gene therapy.

## Methods

### Sex as a biological variable.

In the present study, only male mice were used because MASLD/MASH is a sex-dimorphic disease with a general higher prevalence in men, and estrogen is a key factor in MASLD progression (36–38). Whether the present findings could be applied to female mice still requires further studies. In the immunofluorescence experiments using human liver samples, details of gender information on fibrosis stage are listed (Supplemental Table 2). Statistical analyses was performed by considering sex not as a variable in determining the effect of MASLD/MASH on the expression of CEBPA but there was insufficient statistical power to analyze sex-stratified effects. The human liver samples used for bioinformatic analyses, qPCR, and Western blot were from deidentified human patients and gender information was not available.

### Patient samples.

Deidentified normal (*n* = 13) and MASH human liver (*n* = 28) samples were obtained through the Liver Tissue Cell Distribution System (Minneapolis, Minnesota, USA) and subjected to qPCR and Western blot analyses. For immunofluorescence staining of CEBPA, normal (*n* = 3) livers were obtained from healthy liver donors during liver transplantation, while MASLD liver sections for F0 (*n* = 9), F1 (*n* = 9), F2 (*n* = 7), F3 (*n* = 6), and F4 (*n* = 7) stage from patients with MASLD who underwent liver biopsy to assess liver histopathology after being diagnosed with fatty liver via B-mode ultrasonography. Liver histopathology was assessed by 2 independent pathologists based on MASH Clinical Research Network scoring system as described previously (39). Detailed information of human patients was provided in Supplemental Table 2.

### Mouse studies.

*Cebpa*^fl/fl^, albumin-cre, and albumin-ERT2-cre mice were as described, respectively (40–43). Hepatocyte-specific CEBPA-deficient mice (*Cebpa*^ΔHep^ or *Cebpa*^ΔHep,ERT2^) were generated by breeding *Cebpa*^fl/fl^ mice with Albumin-cre or Albumin-ERT2-cre and backcrossed with C57BL/6N mice for 9 generations. High-fat, high-cholesterol, high-fructose, diet (HFCFD, D09100310, Research Diets) was used to induce MASH-associated fibrosis, while carbon tetrachloride (CCl_4_) was used to chemically induce non-MASH-associated fibrosis. All mice were maintained in the animal facility of National Cancer Institute under specific pathogen-free conditions. For further details regarding the materials and methods, please refer to the supplemental data.

### Statistics.

Sample sizes are presented in the figure legends. No statistical tool was used to predetermine sample sizes; rather, the availability of study materials and estimates of variances based on previous experience determined the number of biological replicates that were used. Statistical analysis was performed using GraphPad Prism (GraphPad Software). Experimental values represent mean ± SEM. Statistical significance between 2 groups was determined using 2-tailed student’s *t* test. 1-way analysis of variance (ANOVA) with Dunnett’s multiple-comparisons, 2-way ANOVA with Šidák’s or Tukey’s multiple-comparisons test, or 3-way ANOVA with Šidák’s multiple-comparisons test were applied for comparisons among multiple groups as indicated in each figure legend. Correlation analyses were assessed by nonparametric Pearson’s test. *P* values were calculated with confidence intervals of 95%. A *P* value less than 0.05 was considered statistically significant.

### Study approval.

All animal handling procedures were conducted in accordance with NIH guidelines and approved by the National Cancer Institute Animal Care and Use Committee with animal protocol number LM096. The protocol for obtaining human liver samples for immunofluorescence was approved by the Ethical Committee of Beijing YouAn Hospital with protocol number LL-2020-091-K. All patients and healthy liver donors provided written informed consent. Human liver samples that were obtained from the Liver and Tissue Cell Distribution System were funded by NIH Contract #HHSN276201200017C.

### Data availability.

The RNA-Seq data in this study were deposited in NCBI’s Gene expression Omnibus and are accessible through GEO Series accession number GSE212646 for the data from mouse livers, while through GSE214615 for the data from primary mouse hepatocytes and GSE248340 for scRNA-Seq data. Values for all data points found in graphs can be found in the Supporting Data Values file.

## Author contributions

TY conceived and designed the study and wrote the manuscript. TY, NY, YX, VS, KH, HY, and HBD performed experiments and data analyses. DA provided expert advice on luciferase reporter design and helped with ChIP-seq bioinformatic analyses. ST, YS, and KWK helped prepare study materials. VS and TJV performed bioinformatic analyses. SK and JH conducted study supervision. YR provided human liver samples for qPCR and Western blot. GL, HL and AQ obtained human liver biopsies for immunofluorescence staining. XZ performed immunofluorescence staining on human livers. FJG, HH, and AQ supervised the study and revised the manuscript.

## Supplementary Material

Supplemental data

Unedited blot and gel images

Supporting data values

## Version 1 04/01/2024

Electronic publication
