# Supplementary material for: Hepatocyte-specific CCAAT/enhancer binding protein α restricts liver fibrosis progression
Source: J Clin Invest. 2024 Apr 1;134(7):e166731. doi: 10.1172/JCI166731 (PMC10977981; doi:10.1172/JCI166731)
Supplement: Supplemental data [file jci-134-166731-s134.pdf]

Hepatocyte-specific CCAAT/enhancer binding protein α restricts liver fibrosis progression

Tingting Yan[1,2], Nana Yan[1,2], Yangliu Xia[1], Vorthon Sawaswong[1], Xinxin Zhu[3], Henrique Bregolin Dias[1], Daisuke Aibara[1], Shogo Takahashi[1], Keisuke Hamada[1], Yoshifumi Saito[1], Guangming Li[4], Hui Liu[5], Hualong Yan[6], Thomas J. Velenosi[1], Kristopher W. Krausz[1], Jing Huang[6], Shioko Kimura[1], Yaron Rotman[7], Aijuan Qu[3,*], Haiping Hao[2,*], Frank J. Gonzalez[1,*]

[1]Cancer Innovation Laboratory, Center for Cancer Research, National Cancer Institute, National Institutes of Health, Bethesda, MD 20892, USA

[2]State Key Laboratory of Natural Medicines, Laboratory of Metabolic Regulation and Drug Target Discovery, China Pharmaceutical University, Nanjing 210009, China.

[3]Department of Physiology and Pathophysiology, School of Basic Medical Sciences, Capital Medical University, PR China; Key Laboratory of Remodeling-Related Cardiovascular Diseases, Ministry of Education; Beijing Key Laboratory of Metabolic Disorder-Related Cardiovascular Diseases. Beijing 100069, PR China.

[4]General Surgery Center, Beijing YouAn Hospital, Capital Medical University, Beijing 100069, PR China.

[5]Department of Pathology, Beijing YouAn Hospital, Capital Medical University, Beijing 10069, PR China.

[6]Cancer and Stem Cell Epigenetics, Laboratory of Cancer Biology and Genetics, Center for Cancer Research, National Cancer Institute, National Institutes of Health, Bethesda, MD 20892, USA

[7]Liver and Energy Metabolism Section, Liver Diseases Branch, National Institute of Diabetes and Digestive and Kidney Diseases, National Institutes of Health, Bethesda, MD 20892, USA

This PDF file includes:
Supplemental Methods
Supplemental References
Supplemental Figures 1-10
Supplemental Tables 1-5

Supplemental Methods

Bioinformatic analyses of published ChIP-seq data, GEO dataset or microarray data. The mouse liver CEBPA ChIP-seq dataset GSE65167(1) or H3K27ac ChIP-seq dataset GSE60430 (2) were downloaded from the GEO database in NCBI (http://www.ncbi.nlm.nih.gov/geo/). The CEBPA and H3K27ac binding peaks of the *Spp1* promoter were analyzed using the Integrated Genome Browser (https://www.bioviz.org/). For gene expression or correlation analyses during MASH that was previously described as NASH, the microarray data were downloaded from https://www.ebi.ac.uk/arrayexpress/experiments/E-MEXP-3291/ with accession number E-MEXP-3291 (3) to analyze hepatic *CEBPA* mRNA and liver fibrosis markers in normal (n = 19), steatosis (n = 10), and MASH (n = 16) human livers. For bioinformatical analyses of mouse MASH livers, GEO dataset was from https://www.ncbi.nlm.nih.gov/ with accession number GSE162276 (4). For Venn plots in **Figure S1**, gene numbers were overlapped between MASH-upregulated DEGs or MASH-downregulated DEGs in the livers in two published datasets (microarray E-MEXP-3291 for human livers and GEO dataset GSE162276 for mouse livers) and the predicted *Cebpa* promoter-binding transcriptional factors listed in **Table S1**. DEGs defined from the pairwise comparisons were required to satisfy two selection criteria: a fold change >1.2 and a corresponding adjusted P value <0.05.

Patient samples. For quantitative polymerase chain reaction (qPCR) and western blot using human liver samples from MASH patients that were previously described as NASH patients, deidentified normal human liver (n = 13) and MASH human liver (n = 28) samples were obtained through the Liver Tissue Cell Distribution System (Minneapolis, Minnesota), among which samples were partially overlapped with the prior microarray study (3). The Liver and Tissue Cell Distribution System was funded by NIH Contract #HHSN276201200017C. For human liver samples used for immunofluorescence staining, normal human liver biopsy specimens were taken from healthy liver donors during liver transplantation, while F0-4 livers from patients who underwent liver biopsies to further assess the histopathology after being diagnosed with fatty liver using B-mode ultrasonography. All patients and healthy liver donors provided written informed consent. The use of all human liver samples for immunofluorescence staining was approved by the Ethical Committee of Beijing YouAn Hospital with protocol number LL-2020-091-K. The F0-4 stage was evaluated by two independent pathologists based on the criteria similarly as described (5). Details about the age and gender of patients were listed in **Table S2**.

Mice. To investigate disease progression, 8- to 12-week-old age-matched male mice were fed a high-fat, high-cholesterol high-fructose, diet (HFCFD, D09100310, Research Diets, New Brunswick, NJ) for indicated time. To chemically induce liver fibrosis in mice, 10-12-week-old age-matched male mice were intraperitoneally dosed with carbon tetrachloride (CCl_4, 289116, Sigma, St. Louis, MO) at 1:40 dilution in corn oil (C8267, Sigma, St. Louis, MO) and then 0.20 mL/20 g mouse) once a week for 4 weeks or 8 weeks, while the mice were killed 3 days after the last CCl_4 dosing. To induce temporal and spatial loss of hepatocyte CEBPA at the later stage of MASH, the *Cebpa*$^{\Delta Hep,ERT2}$ mice were injected with tamoxifen (13258, Cayman, Ann Arbor, MI, USA) at 50 mg/kg in corn oil for the first three days via intraperitoneal injection once daily followed by once weekly dosing for a duration as indicated unless otherwise stated. *Cebpa*$^{\Delta Hep/+}$ and *Cebpa*$^{f/+}$ mice were generated by breeding *Cebpa*$^{\Delta Hep}$ mice with C57bl/6N mice. For AAV8-related experiments, the pAAV8-TBG-*Cebpa*, scAAV-U6-*Spp1* shRNA or each control AAV8

were intravenously injected once at 2.0×10^{11} copies/mouse in 0.2 mL phosphate-buffered saline, except that the mice were intravenously injected at 5×10^{11} copies/mouse for the AAV8-TBG-*Cebpa* therapeutic dosing scheme, in BSL-level 2 hood. In this study, only male mice were used for all the animal experiments or for use of primary hepatocytes or HSC isolation. The mice were housed with no more than 5 mice per cage. Mice were maintained in a temperature-controlled room at 20–24 °C and average humidity at 40% under a standard 12-h light/12-h dark cycle with water and food provided ad libitum. All mouse studies were approved by the National Cancer Institute Animal Care and Use Committee with protocol number LM096 and performed in accordance with the Institute of Laboratory Animal Resources guidelines.

Mouse metabolic studies. Mouse metabolic studies were performed similarly as described (6). For the glucose tolerance test, mice were fasted for 16 h and then intraperitoneally (i.p.) injected with 2 g/kg glucose. For the insulin tolerance test, mice were fasted for 6 h and then intraperitoneally (i.p.) injected with insulin (Eli Lilly, 1:1250 dilution in saline). Blood glucose levels were measured with a glucometer (Bayer, Washington, D.C.) at 0, 15, 30, 60, 90 and 120 min after injection of either glucose or insulin. The area under the curve (AUC) was calculated as the incremental area by the conventional trapezoid rule.

Histological analyses. Formalin-fixed paraffin-embedded liver sections were stained with hematoxylin-eosin (H&E) or Sirius red, and OCT compound-embedded frozen liver sections were used for oil red O staining. All H&E staining, Sirius red staining, periodic acid-Schiff (PAS) staining, terminal deoxynucleotidyl transferase dUTP nick end labeling (TUNEL) staining, and oil red O staining were performed in VitroVivo Biotech (Rockville, MD) according to standard protocols. The histological features of the livers were observed under a digital light microscope (BZ-X710, Keyence, Itasca, IL, USA) for H&E staining, Sirius red staining and CD45 staining, while oil red O staining under another light microscope (ECHO, San Diego, CA, USA) except the pictures in **Figure 8J** under the digital light microscope (BZ-X710, Keyence, Itasca, IL, USA). The quantitation of Sirius red staining was performed using software associated with All-in-One Fluorescence Microscope (BZ-X710, Keyence, Itasca, IL, USA).

Cell lines and luciferase reporter assays. HEK293T cells were obtained as described previously (7) and cultured in Dulbecco's modified Eagle's medium (DMEM, Corning, NY, USA) supplemented with 10% fetal bovine serum (FBS; Gibco, Gaithersburg, MD, USA) and 1% antibiotic (ThermoFisher, Waltham, MA, USA). Both cells were seeded into 24-well plates at 70%-90% confluency. The recombinant human HES1 cDNA was cloned into the backbone FLAG-HA-pcDNA 3.1 vector (8). TheFLAG-HA-pcDNA 3.1 was a gift from Adam Antebi (Addgene plasmid # 52535, RRID: Addgene_52535) and used as empty control vector. Custom luciferase reporter plasmids were constructed in BioInnovatise Inc (Rockville, MD, USA). Custom GeneBlocks containing the predicted CEBPREs were synthesized by IDT DNA Technologies (Coralville, IA) and cloned into the pGL4.11 luciferase vector (E6661, Promega, Madison, WI, USA). CEBPRE mutants were constructed by site-directed mutation through replacing the core sequence "CAA" in CEBPRE1-3 to "TTT" in BioInnovatise Inc (Rockville, MD, USA). In details, CEBPRE1 sequence "TGTCGCAATGGG" was mutated to be "TGTCGTTTTGGG", CEBPRE2 sequence "TTTTACAACGTT" was mutated to be "TTTTATTTCGTT", and CEBPRE3 sequence "TTTTGCAATGCT" was mutated to "TTTTGTTTTGCT". For *Spp1*-Luc promoter deletions, -3000/+26 bp were used to generate the

CEBPRE1-deleting *Spp1*-Luc (-1704/+26 bp) and CEBPRE1+2-deleting *Spp1*-Luc (-1036/+26 bp). The HEK293T cells were transfected with *Spp1* promoter reporter vectors or control empty pGL4.11 luciferase vector at 300 ng/well with 50 ng/well phRL-TK Renilla luciferase control vector (E2241, Promega, Madison, WI, USA) for transfection efficacy normalization by using Lipofectamine 3000 transfection reagent (L3000-015, ThermoFisher, Waltham, MA, USA). At 24 hours after transfection, the luciferase assays were performed using the dual-luciferase reporter assay system (E1960, Promega, Madison, WI, USA). Firefly and Renilla luciferase activities were detected by Veritas microplate luminometer (Turner Biosystems, Sunnyvale, CA, USA). For adenovirus transduction, either a mouse CEBPA-overexpressing adenovirus (Ad-CEBPA, #1477, Vector Biolabs, Malvern, PA, USA) or control GFP-overexpressing adenovirus (Ad-GFP, #1060, Vector Biolabs, Malvern, PA, USA) was added at a concentration of 5000-fold dilution in culture medium 30 min before adding the luciferase reporter vectors. In details, the HEK293T cells were transfected with empty luciferase reporter, full-length (-3000/+26) *Spp1*-Luc or site-directed mutation at CEBPRE1 (ΔMut1), CEBPRE2 (ΔMut2), CEBPRE3 (ΔMut3), CEBPRE1-deleted *Spp1*-Luc (-1704/+26) or CEBPRE1+2-deleted *Spp1*-Luc (-1036/+26) for 24 h in accompany with Ad-GFP or Ad-CEBPA treatment as indicated.

Biochemical analyses and ELISA assay. Liver TC/TG/NEFA and serum TC/TG/NEFA/ALT levels were measured with assay kits from Wako Diagnostics (Wako Chemicals USA, Richmond, VA) following the manufacturer's instruction. OPN levels in the serum or cell culture supernatant were measured using mouse osteopontin ELISA kit (RAB0437, Sigma, St. Louis, MO) by diluting the serum or supernatant for 500-3000 folds.

scRNA-seq library preparation and data analyses for primary liver cells. Liver cell dissociation was carried out based on the *in-situ* liver perfusion-digestion method as described in a previous study (9). The isolated cells were cleaned-up using Percoll (P1644-500ML, Sigma, Burbank, CA, USA) density gradient centrifugation (36% final conc.) at 1600 rpm, 4 °C for 10 min. After centrifugation, the cell suspension was washed with 1×PBS and the cell viability counted based on AO/PI straining using Nexcelom Cellometer Auto 2000 (Nexcelom Bioscience, Lawrence, MA, USA) to help examine and maintain the cell viability a >90% for use in the library preparation. To guarantee the presence of non-parenchymal cells, the hepatocytes were partially removed after natural sedimentation by standing in cold ice for 5 min. The single cells from mouse livers were loaded in the lanes according to the 10X Genomics 3' v3.1 Single Cell User Guide with a single capture lane per sample targeting recovery of up to 6,000 cells per lane. All subsequent steps, GEM recovery, cDNA generation, and quality control were performed as described in the 10X Genomics 3' v3.1 Single Cell kit (1000268, 10X Genomics, Pleasanton, CA, USA). Libraries were prepared using the 10X Genomics validated automation platform, Chromium Connect. Sequencing was performed on Illumina NextSeq platform (Illumina, USA) in the NCI Center for Cancer Research sequencing facility. Base calling was performed using RTA 3.10.30, demultiplexing was performed using Cellranger v7.1.0 (Bcl2fastq 2.20.0), and alignment performed using Cellranger v7.1.0 (STAR 2.7.2a). Sequence reads were aligned to the mouse reference sequence provided by 10X Genomics (refdata-gex-mm10-2020-A). The Cellranger software (10X Genomics, Pleasanton, CA, USA) was used for aggregating and normalizing the data among samples based on the default parameters. The data were further analyzed by Loupe browser 7.0.1 (10X Genomics, Pleasanton, CA, USA) to examine the gene expression in single cells and classified the cell types based on the markers described in **Table S3**.

Chromatin immunoprecipitation assay. The primary hepatocytes were isolated from 10-week-old male C57BL/6N mice and seeded in 100 mm collagen-coated dish (5028-10EA, Advanced BioMatrix, Carlsbad, CA, USA) at a 90% confluency in Williams Medium E (Gibco, Gaithersburg, MD, USA) containing 1% antibiotics (equal mixture of 0.5% penicillin and 0.5% streptomycin; Gibco, Gaithersburg, MD, USA) and primary hepatocyte maintenance supplements (CM4000, Gibco, Gaithersburg, MD, USA) overnight before treatment. The cells were treated with either a mouse CEBPA-overexpressing adenovirus (1:3500 dilution, 10^10 PFU/mL; Ad-CEBPA, #1477, Vector Biolabs, Malvern, PA, USA) or control GFP-overexpressing adenovirus (1:3500 dilution, 10^10 PFU/mL; Ad-GFP, #1060, Vector Biolabs, Malvern, PA, USA) for 48 additional h. Then, the cells were fixed with 1% formaldehyde at room temperature for 10 min and quenched with 0.125 M glycine at room temperature for 5 min. Cells were collected and chromatin was prepared using the procedure as described (10). Isolated chromatin was further fragmented into 200 bp-400 bp using Bioruptor. Two µg of CEBPA (Cell Signaling, 8178S) and two µL of other antibodies including HDAC3 (Abcam, ab4729), H3K27ac (Abcam, ab4729), H3K4mer3 (Cell Signaling, 9751S) and Rabbit IgG (Cell Signaling, 2729S) were used for each immunoprecipitation sample, among which H3K4me3 served as positive control and rabbit IgG as a negative control. The purified DNA was dissolved in 1 mL of TE buffer and used as DNA template for qPCR analyses. Data were normalized to input DNA and fold change was then calculated as indicated. Primer sequences were provided in **Table S4**. Antibodies are listed in **Table S5**.

Western blotting. Tissues or cultured cells were lysed with RIPA lysis buffer in the presence of protease inhibitors, with protein concentrations determined by the BCA protein assay kit (23227, Pierce Chemical, Rockford, IL). The samples were subjected to SDS-polyacrylamide gel electrophoresis, transferred to polyvinylidene fluoride membranes, and incubated overnight at 4 °C with antibodies against CEBPA (8178, Cell Signaling Technology, Danvers, MA, USA), beta-actin (ACTB, 4970, Cell Signaling Technology, Danvers, MA, USA), HES1 (A11718, Abclonal, Woburn, MA, USA), anti-FLAG antibody (AE005, Abclonal, Woburn, MA, USA), and alpha/beta-tubulin (TUBULIN, 2148, Cell Signaling Technology, Danvers, MA, USA). Mouse osteopontin (OPN) antibody was purchased from R&D (AF808, R&D, Minneapolis, MN, USA). Proteins were visualized using the SuperSignalTM West Dura Extended Duration Substrate (34076, ThermoFisher, Waltham, MA, USA) with an image analyzer (Alpha Innotech Corp., San Leandro, CA, USA). Image J (National Institutes of health, Bethesda, MD, USA) was used for quantitation analyses of the blotting bands. Antibodies are listed in **Table S5**.

Isolation of primary hepatocytes, non-parenchymal cells, and hepatic stellate cells. Primary hepatocytes were isolated from 8-10-week-old male mice similarly as described (11). Briefly, perfusion medium were made by adding 100 µL of EDTA (RGC-3130, 0.5 M) into each 50 mL of HBSS (14175-079, Gibco, Gaithersburg, MD, USA), and the digestion medium were made by adding 37 mg of $CaCl_2$, 15 mg of Collagenase Type I (17100017, Gibco, Gaithersburg, MD) and 15 mg of Collagenase Type II (17101015, Gibco, Gaithersburg, MD) into each 50 mL of HBSS. The livers were exposed immediately after the mice were euthanized with CO_2 inhalation and perfused with perfusion medium followed by pre-warmed digestion medium through the portal vein at 4 mL/min for 6 min, respectively. Next, the liver was excised and minced in DMEM culture medium that contains 10% FBS, and then filtered through a 75 µm steel mesh and hepatocytes were collected by centrifugation at 50 × g for 2 min. The obtained hepatocytes were resuspended

in 10 mL complete culture medium and then added 90% Percoll solution (diluted in 10 × PBS at a ratio 9:1 so that the final PBS concentration is 1×). The suspended cells were mixed immediately and gently after adding Percoll solution and then centrifugated at 600 rpm for 10 min at 4 ºC to collect the alive hepatocytes in the pellet. The hepatocytes were washed with 1 × PBS once before seeding in complete culture medium, Williams Medium E (A12117601, ThermoFisher, Waltham, MA) containing 1% antibiotics (equal mixture of 0.5% penicillin and 0.5% streptomycin; Gibco, Gaithersburg, MD, USA) and primary hepatocyte maintenance supplements (CM4000, Gibco, Gaithersburg, MD, USA) overnight before treatment.

Primary non-parenchymal cells from mouse liver tissues were isolated and enriched during isolation of primary hepatocytes similarly as described (9). The remaining supernatant after centrifugation at 50 × g for 2 min was collected and then centrifuged at 50 × g for 3 min for 4 times, during which the cells were suspended in 10% DMEM. Subsequently, the supernatant was centrifuged at 580 × g for 5 min, and then pellet collected for further analyses.

Primary hepatic stellate cells (HSC) were isolated from 10-month-old male C57BL/6N mice similarly as described previously by using the OptiPrep-based Density Gradient Medium (D1556, Sigma, St. Louis, MO) and following a density gradient-based separation (12, 13). All isolated HSC cells were pooled together and seeded in poly-lysine-coated 6-well plates (356515, Corning, NY, USA) in complete DMEM containing 10% FBS and 1% antibiotics (equal mixture of 0.5% penicillin and 0.5% streptomycin; Gibco, Gaithersburg, MD, USA).

Treatment of primary hepatocytes. For trichostatin A (TSA) treatment in primary hepatocytes were isolated from either *Cebpa*$^{fl/fl}$ or *Cebpa*$^{\Delta Hep}$ mice as indicated and seeded in 12-well collagen-coated plates at an equal density of 3×10^5 cells overnight prior treatment. TSA (TargetMol, Cat#T6270) were dissolved in DMSO first and then diluted 1000 times yielding final concentrations of 2 μM, 6 μM and 30 μM. The primary hepatocytes were treated with TSA at 0, 2 μM, 6 μM and 30 μM for 48 h prior to collection for further mRNA analyses. The Ad-GFP or Ad-CEBPA (1:3500 dilution for each) were added at the same time together with TSA or control vehicle. For Ad-ATF3 or Ad-GFP treatment, the primary mouse hepatocytes were freshly isolated from chow-fed 10-week-old C57BL/6N mice and seed overnight prior treatment, followed by treatment of Ad-GFP or Ad-ATF3 (1:3500 dilution) for additional 48 hours. Ad-GFP (Cat#1060), Ad-CEBPA (Cat#1477) and Ad-ATF3 (Cat#ADV-253206) were obtained from Vector Biolabs (Malvern, PA, USA). For HES1 overexpression experiments in primary human hepatocytes, primary human hepatocytes in 12-well collagen-coated plates were obtained from Xenotech (Kansas, KS, USA). The primary human hepatocytes were placed in a 37 ºC incubator for 24 h and then treated with 2 μg of recombinant HES1 plasmid or matched control empty vector plasmid for additional 48 hours using Lipofectamine 3000 (L3000-015, ThermoFisher, Waltham, MA, USA). The custom recombinant human HES1 overexpression plasmid and control plasmid were the same as described in the "Cell lines and luciferase reporter assays" section above.

shRNA and neutralizing antibody in vitro experiments. Primary hepatocytes were isolated from 12-week-old male *Cebpa*$^{fl/fl}$ and *Cebpa*$^{\Delta Hep}$ mice and seeded at collagen-coated 12-well plates (354500, Corning, NY, USA) at an equal density of 3×10^5 cells per well. The overnight-attached cells were transfected with 2 μg of control scrambled shRNA (CCTAAGGTTAAGTCGCCCTCGCTCGAGCGAGGGCGACTTAACCTTAGG) or sh*Spp1* (CTCTTAGCTTAGTCTGTTGTTTCAAGAGAACAACAGACTAAGCTAAGA) (14). Both shRNA

plasmids were transfected into primary hepatocytes using Lipofectamine 3000 (L3000-015, ThermoFisher, Waltham, MA, USA) in the presence of 0.4 mM palmitic acid (PA, Sigma) according to the manufacturer's instructions. The conditional culture medium was collected from supernatant of primary hepatocytes at 48 h after shRNA plasmid transfection and stocked in -80 ºC until use, while GFP fluorescence was observed to monitor the transfection efficacy. The conditioned culture medium was collected and equally diluted 2 times in fresh complete DMEM medium for each group and then used to treat the primary mouse HSCs for 48 h. For OPN neutralizing experiment, the OPN neutralizing antibody (AF808, R&D systems, Minneapolis, MN, USA) at the recommended ND50 of 1 μg/mL was directly added to the conditional culture medium for 30 min-1 h prior to HSC incubation, which was similarly as described previously (14).

AAV8 virus vectors. AAV8 viruses were produced, purified, and titrated in accordance with the previous paper (15). Briefly, AAV8-*Spp1*-shRNA and AAV8-Scrambled shRNA viruses were generated by transfection of 293FT cells (Thermo Fisher Scientific, #R70007) with the AAV-sh*Spp1* (CTCTTAGCTTAGTCTGTTGTTTCAAGAGAACAACAGACTAAGCTAAGAG) as described (14) or shRNA-Scramble (VectorBuilder, Guangzhou, China; RRID: Addgene plasmid #1864) as described (16) under the control of the mouse U6 promoter and flanked by serotype-2 inverted terminal repeats, cap genes of AAV serotype-8 (Addgene, pAAV2/8, #112864), and pHelper vector (Addgene, pAdDeltaF6, #112867). AAV plasmids containing either ORF of mouse *Cebpa* (42 kD isoform, NM_007678.3) or mCherry under the control of the Human thyroxine-binding globulin promoter (TBG) (17), were used to produce AAV8-m*Cebpa* and AAV8-mCherry as control virus and made in VectorBuilder (VectorBuilder, Guangzhou, China).

RNA-seq library preparation and data analyses for liver tissues. For liver samples, livers from 22-week HFCFD-fed *Cebpa*$^{\Delta Hep,ERT2}$ or *Cebpa*$^{fl/fl}$ mice with tamoxifen dosing for the final 10 weeks that showed a marked phenotype of enhanced liver fibrosis, 8-week HFCFD-fed *Cebpa*$^{\Delta Hep,ERT2}$ mice and *Cebpa*$^{fl/fl}$ mice with tamoxifen dosing for final 10 days as well as 2-week HFCFD-fed *Cebpa*$^{\Delta Hep}$ or *Cebpa*$^{fl/fl}$ mice that showed an earlier or no phenotype of enhanced fibrosis were employed. In details, RNA-seq were performed for 3 sets of livers from 2-week HFCFD-fed mice (HFCFD2W), 8-week HFCFD-fed mice that were dosed with tamoxifen for the last 10 days (HFCFD8W+T10D), 22-week HFCFD-fed mice that were dosed with tamoxifen for the last 10 weeks (HFCFD12W+T10W).

For hepatocyte samples, primary hepatocytes were freshly isolated from 10-week-old wild-type *Cebpa*$^{fl/fl}$ (WT) or hepatocyte-specific *Cebpa* knockout (*Cebpa*$^{\Delta Hep}$) (KO) mice, and then subject to RNA-seq analyses for WT group and KO group. Meanwhile, primary hepatocytes isolated from 10-week-old C57BL/N mice were seeded overnight, followed by Ad-GFP or Ad-CEBPA transduction for additional 48 hours and then collected for RNA-seq analyses for control GFP group and CEBPA-overexpressed (OE) group, respectively. The differential gene expression (DGE) was analyzed between WT and KO group as well as between GFP and OE group, followed by a heatmap analysis to show the overlapped genes in DGE between the consistently-changed genes in these two comparisons (WT versus KO, GFP versus OE) and those in the former comparisons among 3 RNA-seq datasets for livers.

RNA was isolated from these mouse livers or primary hepatocytes using RNeasy Plus Mini Kit (#74136, Qiagen, Germantown, MD) according to the manufacturer's instructions. All samples had a RIN value of at least 8.0 and passed the quality control test by TapeStation test. RNA-seq libraries were prepared using Illumina TrueSeq Stranded Total RNA Library Prep

(Illumina, San Diego, CA). The samples were sequenced on NextSeq platform (Illumina) using a paired-end protocol with 25 million reads per sample. For RNA-seq data analysis, the data were aligned to mouse reference genome (mm10). To exclude those low-expressed genes, the RNA-seq data were filtered with the CPM value set at a threshold of >200. DGE was assessed with DEseq2 using the parameters: adjusted *P* value of 0.05 and log2 fold change (log2FC) of 1 (for 2-fold differentially expressed genes). Volcano plots were prepared using EnhancedVolcano package based on R program. Ingenuity Pathways Analysis (IPA, version 76765844, QIAGEN, Germantown, MD) was used to perform IPA analyses based on the differentially expressed genes (adjusted *P* value <0.05 and fold-change>2). The top 10 enriched canonical pathways with lowest *P* values were plotted in the balloon plot using GraphPad Prism Version 9.4.1 (GraphPad Software, San Diego, CA, USA).

Prediction of candidate transcriptional factors on the Cebpa promoters and prediction of candidate CEBPREs on the Spp1 promoter. The potential transcriptional factors on *Cebpa* promoters were predicted by JASPAR database (http://jaspar.genereg.net/). The region covering from upstream 10,000 bp to downstream 3,000 bp region of the *Cebpa* gene transcription initiation site (TSS) was designated as the examined promoter region, while other parameters were set at default. The CEBPREs located at *Spp1* promoter region were predicted by JASPAR database (http://jaspar.genereg.net/) with the upstream 3,000 bp to downstream 3,000 bp region of *Spp1* gene transcription initiation site (TSS) set as the promoter region and other parameters set at default.

Macrophage depletion study. For the short-term macrophage depletion study, age-matched 8-week-old *Cebpa*$^{fl/fl}$ mice and *Cebpa*$^{\Delta Hep}$ mice were dosed with clodronate liposome or control liposomes (Encapsula Nano Sciences, Brentwood, TN, USA) via intravenous injection at 0.2 mL/mouse, followed by 0.1 mL/mouse twice a week. All mice were fed a HFCFD for a total of 2 weeks starting 2 days after the first dose of clodronate and killed 2 days after the final dose of clodronate. For long-term macrophage depletion, 12-week-old *Cebpa*$^{fl/fl}$ mice and *Cebpa*$^{\Delta Hep,ERT2}$ mice were fed a HFCFD for 15 weeks and then treated with AAV8-*Spp1*-shRNA and AAV8-Scrambled shRNA at 2×10^{11} copies/mouse once via intravenous injection. One week later, the mice were treated with clodronate or control liposomes at 0.2 mL/mouse via intravenous injection. All mice were dosed with tamoxifen at 50 mg/kg via intraperitoneal injection once daily for 3 consecutive days starting 2 days after clodronate dosing followed by repeated tamoxifen dosing at 50 mg/kg via intraperitoneal injection once a week. All mice were dosed with clodronate or control liposomes at 0.1 mL/mouse twice a week via intravenous injection following the first dose of clodronate dosing at 3- to 4- day intervals between each dose for a total of 4 weeks. All mice were maintained on HFCFD feeding during the whole course of the experiments and killed 2 days after the final dose of clodronate.

Immunofluorescence staining and immunohistochemistry analyses. Immunofluorescence staining for human liver samples was performed using the methods similarly to that described earlier (6), while immunohistochemistry analyses and immunofluorescence staining of mouse livers were as described earlier (18). CEBPA antibody (2295, Cell Signaling Technology, Danvers, MA, USA), F4/80 antibody (70076, Cell Signaling Technology, Danvers, MA, USA), HepPar1 antibody (NBP2-45272, Novus Biologicals, Centennial, CO, USA), Desmin antibody (ab15200, Abcam, Waltham, MA, USA), HNF4α antibody (ab41898, Abcam, Waltham, MA,

USA), OPN antibody (88742, Cell Signaling Technology, Danvers, MA, USA) and CD45 antibody (ab10558, Abcam, Waltham, MA, USA) were used.

Quantitative polymerase chain reaction analyses (qPCR). Tissues were flash frozen in liquid nitrogen and stored at -80 °C. Total RNA from frozen tissues was extracted with TRIzol reagent (Invitrogen, Carlsbad, CA). cDNA was synthesized from 1 µg of total RNA using qScript cDNA SuperMix (Gaithersburg, MD). Analysis was performed by using the ABI PRISM 7900 Sequence Detection System (Applied Biosystems, Bedford, MA). Values were normalized to beta-Actin (*Actb*) or glyceraldehyde-3-phosphate dehydrogenase (*Gapdh*) mRNAs and the results expressed as fold change relative to the control group. Primer sequences are provided in **Table S4**.

Supplemental References

1. Sun X, et al. Suppression of the SWI/SNF component arid1a promotes mammalian regeneration. *Cell Stem Cell.* 2016;18(4):456-466.

2. Sobel JA, et al. Transcriptional regulatory logic of the diurnal cycle in the mouse liver. *PLoS Biol.* 2017;15(4):e2001069.

3. Lake AD, et al. Analysis of global and absorption, distribution, metabolism, and elimination gene expression in the progressive stages of human nonalcoholic fatty liver disease. *Drug Metab Dispos.* 2011;39(10):1954-1960.

4. Lee SM, et al. Hepatocyte-specific loss of PPAR gamma protects mice from NASH and increases the therapeutic effects of rosiglitazone in the liver. *Cell Mol Gastroenterol Hepatol.* 2021;11(5):1291-1311.

5. Kleiner D, et al. Design and validation of a histological scoring system for nonalcoholic fatty liver disease. *Hepatology.* 2005;41(6):1313-1321.

6. Yan T, et al. Intestinal peroxisome proliferator-activated receptor alpha-fatty acid-binding protein 1 axis modulates nonalcoholic steatohepatitis. *Hepatology.* 2023;77(1):239-255.

7. Yagai T, et al. Feedback repression of PPAR alpha signaling by Let-7 microRNA. *Cell Rep.* 2021;36(6):109506.

8. Horn M, et al. DRE-1/FBXO11-dependent degradation of BLMP-1/BLIMP-1 governs C. elegans developmental timing and maturation. *Dev Cell.* 2014;28(6):697-710.

9. Liu D, et al. TNFAIP3 interacting protein 3 overexpression suppresses nonalcoholic steatohepatitis by blocking TAK1 activation. *Cell Metab.* 2020;31(4):726-740.

10. Li M, et al. Genome-wide studies of the transcriptional regulation by p53. *Biochim Biophys Acta.* 2012;1819(7):684-687.

11. Xia Y, et al. Withaferin A alleviates fulminant hepatitis by targeting macrophage and NLRP3. *Cell Death Dis.* 2021;12(2):174.

12. Mederacke I, et al. High-yield and high-purity isolation of hepatic stellate cells from normal and fibrotic mouse livers. *Nat Protoc.* 2015;10(2):305-315.

13. Modak RV, and Zaiss DM. Isolation and culture of murine hepatic stellate cells. *Bio Protoc.* 2019;9(21).

14. Zhu C, et al. Hepatocyte Notch activation induces liver fibrosis in nonalcoholic steatohepatitis. *Sci Transl Med.* 2018;10(468):eaat0344.

15. Aibara D, et al. Gene repression through epigenetic modulation by PPARA enhances hepatocellular proliferation. *iScience.* 2022;25(5):104196.

16. Sarbassov DD, et al. Phosphorylation and regulation of Akt/PKB by the rictor-mTOR complex. *Science.* 2005;307(5712):1098-1101.

17. Yan Z, et al. Human thyroxine binding globulin (TBG) promoter directs efficient and sustaining transgene expression in liver-specific pattern. *Gene.* 2012;506(2):289-294.

18. Feng D, et al. Monocyte-derived macrophages orchestrate multiple cell-type interactions to repair necrotic liver lesions in disease models. *J Clin Invest.* 2023;133(15):e166954.

Supplemental Figures

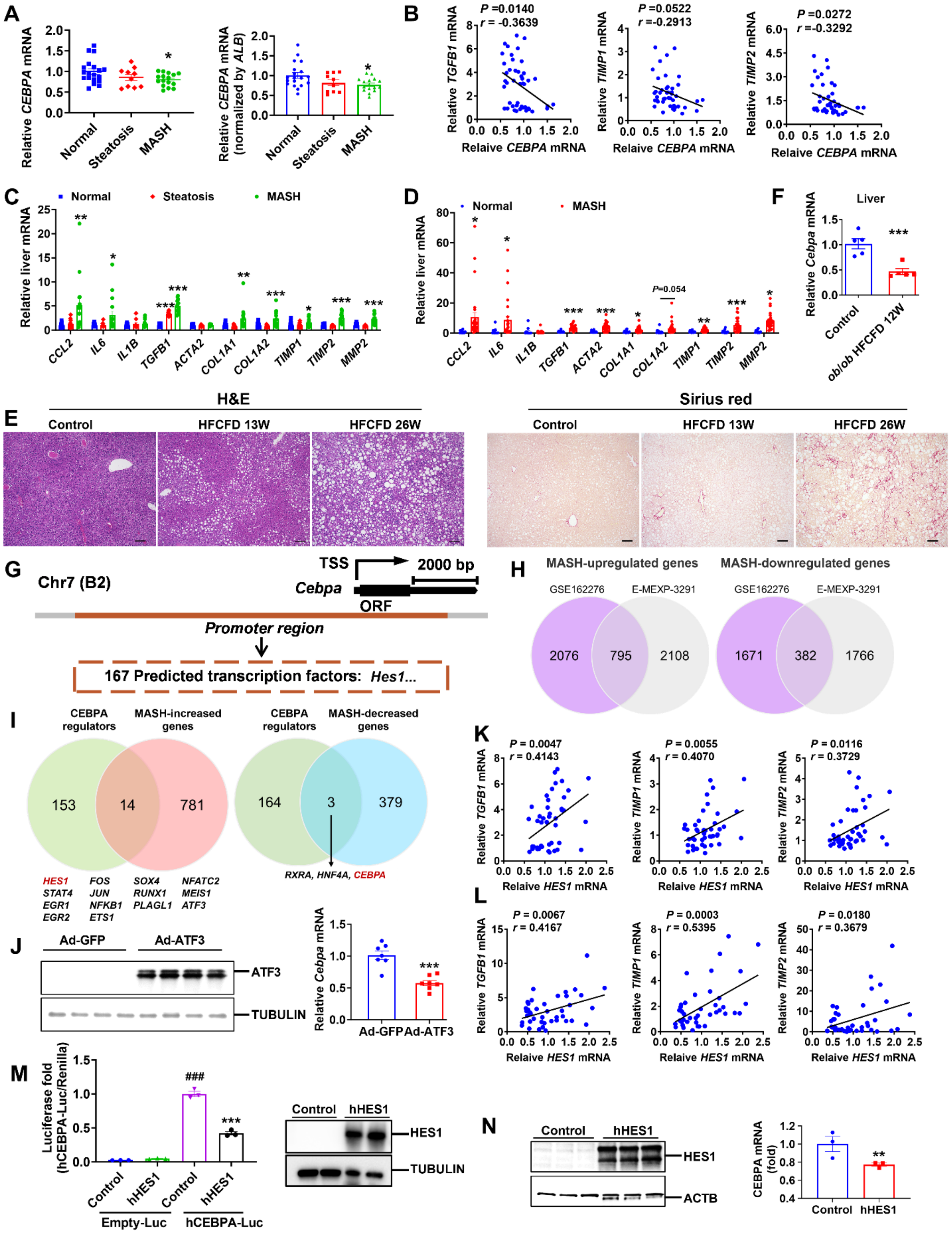

Figure S1. Effect of liver fibrosis on hepatic *CEBPA* mRNA expression and potential modulators of *CEBPA* mRNA expression during MASH. (A-C) Liver *CEBPA* mRNA (A), correlation analyses of *CEBPA* mRNA with *TGFB1*, *TIMP1* and *TIMP2* (B) mRNA in normal, steatosis and MASH group and gene mRNAs in liver fibrosis and inflammation (C) for microarray dataset E-MEXP-3291; *n* = 19 for control normal, *n* = 10 for steatosis, and *n* = 16 for MASH human livers. (D) Gene mRNAs in liver fibrosis and inflammation by qPCR; *n* =13 for normal control and *n* = 28 for MASH human livers. (E) H&E staining and Sirius red staining for the livers of control, 13-week HFCFD-fed, 26-week HFCFD-fed mice. (F) Liver *Cebpa* mRNA in 12-week HFCFD-fed *ob*/*ob* mice; *n* = 5. (G) Prediction of *Cebpa* promoter-binding transcription factors by JASPAR database. (H) Venn plot of the overlapped MASH-upregulated and MASH-downregulated gene numbers between GEO dataset GSE162276 and microarray dataset E-MEXP-3291. (I) Venn plot of MASH-regulated genes overlapping with CEBPA regulators. (J) ATF3 protein expression and *Cebpa* mRNA in primary mouse hepatocytes treated with Ad-GFP or Ad-ATF3 for 48h; *n* = 7. (K-L) Correlation analyses of *HES1* mRNA with fibrosis markers *TGFB1*, *TIMP1* and *TIMP2* in microarray dataset E-MEXP-3291 (K) or in our human samples (L). (M) Luciferase reporter assay in HEK293T cells transfected with human CEBPA promoter (-3000 bp/+26 bp)-driven luciferase reporter (hCEBPA-Luc) or control empty luciferase reporter (Empty-Luc) in the presence of control or human HES1 overexpression plasmid (M left) and HES1 expression detected by using anti-flag antibody in HEK293T cells (M right, *n* = 4). (N) HES1 protein of recombinant human HES1 plasmid-transfected primary human hepatocytes (N left) and *CEBPA* mRNA expression (N right); *n* = 3. Data represent mean ± SEM. **P* < 0.05, ***P* < 0.01, ****P* < 0.001 compared to control normal human livers by one-way ANOVA analyses for **Figure S1A**, **C,** two-way ANOVA analyses with Šídák's multiple comparisons test for **Figure S1M** or two-tailed unpaired student's *t* test for others. Correlation analyses were assessed by non-parametric Pearson's test. Scale bar 100 μm.

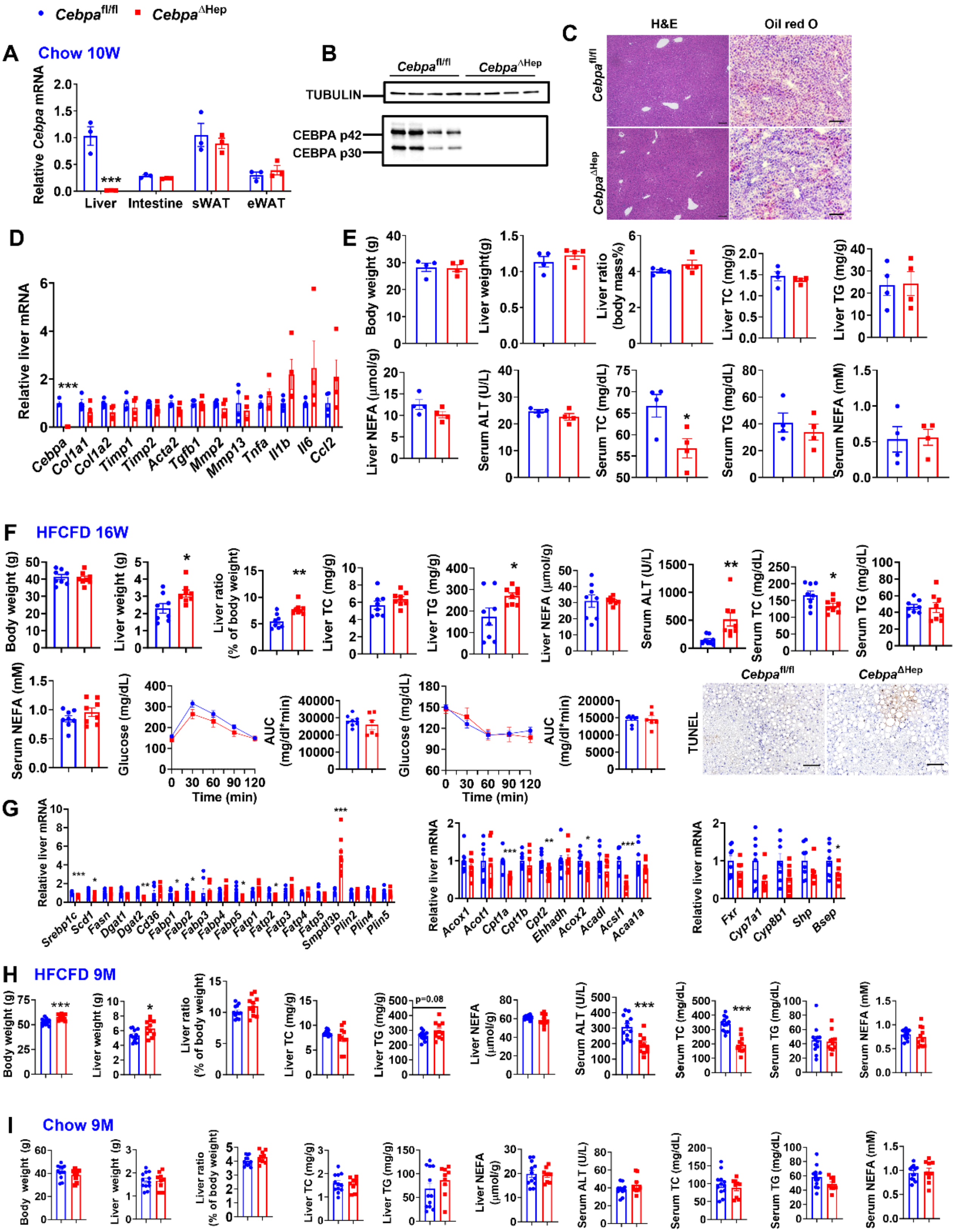
Cebpa fl/fl
Cebpa ΔHep
A Chow 10W
Relative Cebpa mRNA
Liver
Intestine
sWAT
eWAT
B
TUBULIN
CEBPA p42
CEBPA p30
C
H&E
Oil red O
D
Relative liver mRNA
Cebpa
Col1a1
Col1a2
Timp1
Timp2
Acta2
Tgfb1
Mmp2
Mmp13
Tnfa
Il1b
Il6
Ccl2
E
Body weight (g)
Liver weight(g)
Liver ratio (body mass%)
Liver TC (mg/g)
Liver TG (mg/g)
Liver NEFA (μmol/g)
Serum ALT (U/L)
Serum TC (mg/dL)
Serum TG (mg/dL)
Serum NEFA (mM)
F HFCFD 16W
Liver ratio (% of body weight)
Liver NEFA (μmol/g)
Glucose (mg/dL)
Time (min)
AUC (mg/dl*min)
TUNEL
G
Srebp1c
Scd1
Fasn
Dgat1
Dgat2
Cd36
Fabp1
Fabp2
Fabp3
Fabp4
Fabp5
Fatp1
Fatp2
Fatp3
Fatp4
Fatp5
Smpdl3b
Plin2
Plin4
Plin5
Acox1
Acot1
Cpt1a
Cpt1b
Cpt2
Ehhadh
Acox2
Acadl
Acsl1
Acaa1a
Fxr
Cyp7a1
Cyp8b1
Shp
Bsep
H HFCFD 9M
p=0.08
I Chow 9M

Figure S2. Biochemical parameters of *Cebpa*$^{\Delta Hep}$ mice fed a chow diet or HFCFD. (A-B) *Cebpa* mRNA in the liver, intestine, subcutaneous white adipose tissue (sWAT) and epididymal white adipose tissue (eWAT) (A, *n* = 3) and liver CEBPA p42 and CEBPA p30 protein in 8-week-old chow-fed *Cebpa*$^{fl/fl}$ or *Cebpa*$^{\Delta Hep}$ mice (B, *n* = 4). (C-E) H&E (scale bar 100 μm) and Oil red O staining (scale bar 50 μm) (C), gene mRNAs in liver fibrosis and inflammation (D, *n* = 4) and body weights, liver weights, liver weight ratios, liver TG, liver TC, liver NEFA, serum ALT, serum TC, serum TG, serum NEFA (E, *n* = 4) in 10-week chow diet-fed *Cebpa*$^{fl/fl}$ or *Cebpa*$^{\Delta Hep}$ mice. (F) Body weights, liver weights, liver weight ratios, liver TC, liver TG, liver NEFA, serum ALT, serum TC, serum TG, serum NEFA, glucose tolerance test and quantitation, insulin tolerance test and quantitation (*n* = 8), and representative TUNEL staining (scale bar 100 μm) of livers in 16-week HFCFD-fed *Cebpa*$^{fl/fl}$ or *Cebpa*$^{\Delta Hep}$ mice, except that glucose tolerance test were performed with 13-week HFCFD-fed *Cebpa*$^{fl/fl}$ (*n* = 8) or *Cebpa*$^{\Delta Hep}$ mice (*n* = 6), while insulin tolerance test with 14-week HFCFD-fed *Cebpa*$^{fl/fl}$ (*n* = 8) or *Cebpa*$^{\Delta Hep}$ mice (*n* = 6). (G) Liver gene mRNAs in lipogenesis, beta-oxidation, and bile acids signaling in 16-week HFCFD-fed *Cebpa*$^{fl/fl}$ or *Cebpa*$^{\Delta Hep}$ mice; *n* = 8. (H) Body weights, liver weights, liver weight ratios, liver TC, liver TG, liver NEFA levels, serum ALT, serum TC, serum TG, serum NEFA in 9-month HFCFD-fed *Cebpa*$^{fl/fl}$ or *Cebpa*$^{\Delta Hep}$ mice, *n* = 12 for *Cebpa*$^{fl/fl}$ mice and *n* = 11 for *Cebpa*$^{\Delta Hep}$ mice. (I) Body weights, liver weights, liver weight ratios, liver TG, liver NEFA, liver TC , serum ALT, serum TC, serum TG and serum NEFA in 9-month chow diet-fed *Cebpa*$^{fl/fl}$ or *Cebpa*$^{\Delta Hep}$ mice; *n* = 12 for *Cebpa*$^{fl/fl}$ or *n* = 9 for *Cebpa*$^{\Delta Hep}$ mice. Data represent mean ± SEM. **P* < 0.05, ***P* < 0.01, ****P* < 0.001 by two-tailed unpaired student's *t* test.

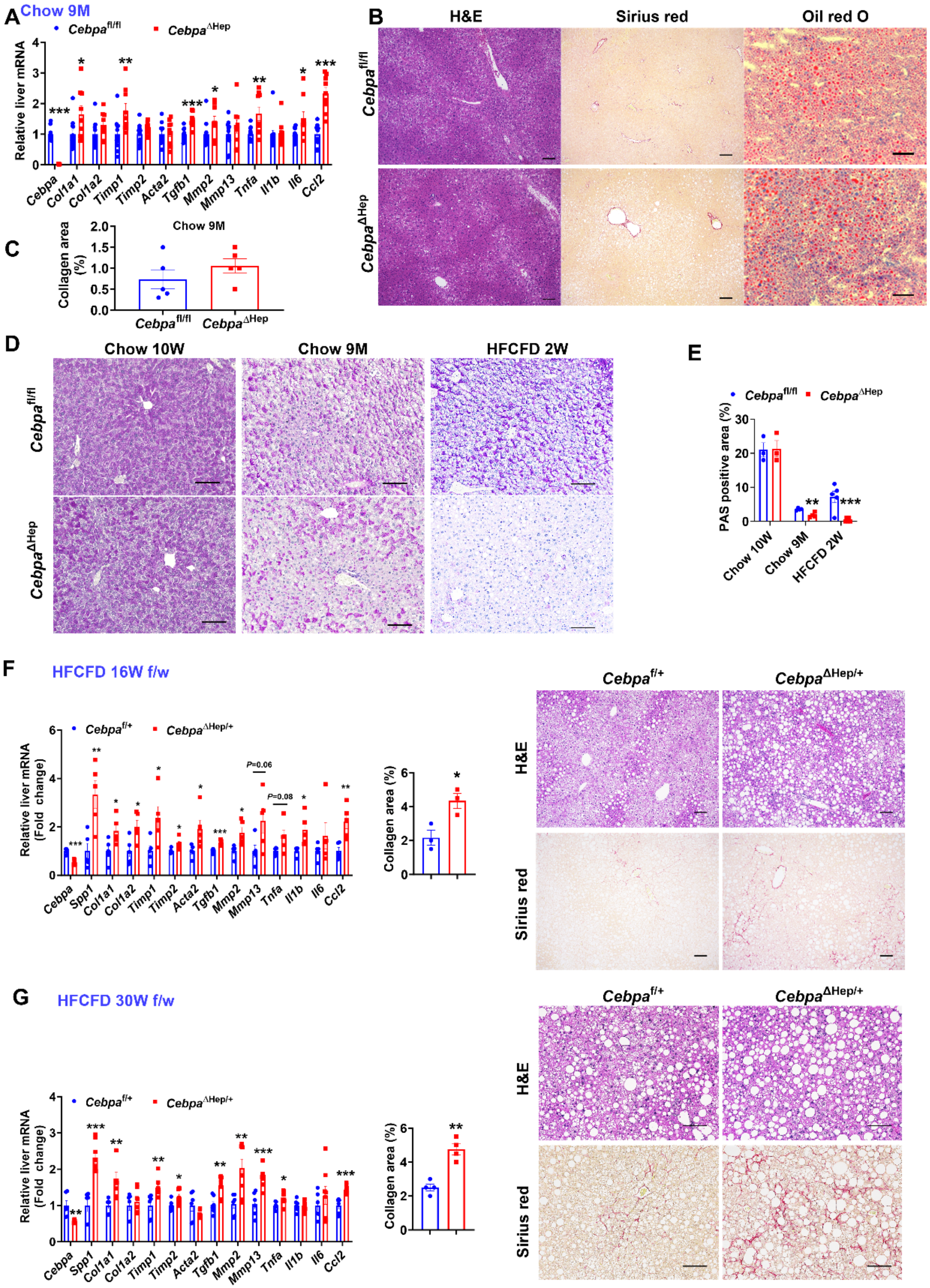

A Chow 9M
Cebpa^fl/fl
Cebpa^ΔHep
Relative liver mRNA
Cebpa
Col1a1
Col1a2
Timp1
Timp2
Acta2
Tgfb1
Mmp2
Mmp13
Tnfa
Il1b
Il6
Ccl2
B
H&E
Sirius red
Oil red O
C
Chow 9M
Collagen area (%)
D
Chow 10W
Chow 9M
HFCFD 2W
E
PAS positive area (%)
F
HFCFD 16W f/w
Cebpa^f/+
Cebpa^ΔHep/+
Relative liver mRNA (Fold change)
Spp1
P=0.06
P=0.08
G
HFCFD 30W f/w

Figure S3. Levels of liver fibrosis and glycogen in chow diet-fed *Cebpa*$^{\Delta Hep}$ mice and liver fibrosis in HFCFD-fed *Cebpa*$^{\Delta Hep/+}$ mice. (A-C) Liver mRNAs in fibrosis and inflammation and histological staining for mice fed a chow diet for 9 months (A-B, *n* = 12 for *Cebpa*$^{fl/fl}$ or *n* = 9 for *Cebpa*$^{\Delta Hep}$ mice) and quantitation of liver Sirius red staining (C, *n* = 5). (D-E) Representative liver PAS staining (D) and quantitation (E) for mice fed a chow diet for 10 weeks (*n* = 3), 9 months (*n* = 4) and HFCFD for 2 weeks (*n* = 5). (F) Liver mRNAs in fibrosis and inflammation and histological staining for *Cebpa*$^{\Delta Hep/+}$ mice fed a HFCFD for 16 weeks (F, *n* = 5) and quantitation of liver Sirius red staining (*n* = 3). (G) Liver mRNAs in fibrosis and inflammation and histological staining for *Cebpa*$^{\Delta Hep/+}$ mice fed a HFCFD for 30 weeks (*n* = 6 for *Cebpa*$^{f/+}$ mice and *n* = 8 for *Cebpa*$^{\Delta Hep/+}$ mice) and quantitation of liver Sirius red staining (*n* = 4). PAS, periodic acid-Schiff. Data represent mean ± SEM. *$P < 0.05$, **$P < 0.01$, ***$P < 0.001$ by two-tailed unpaired student's *t* test. Scale bar 100 μm for H&E staining, Sirius red staining and PAS staining, while 50 μm for Oil red O staining.

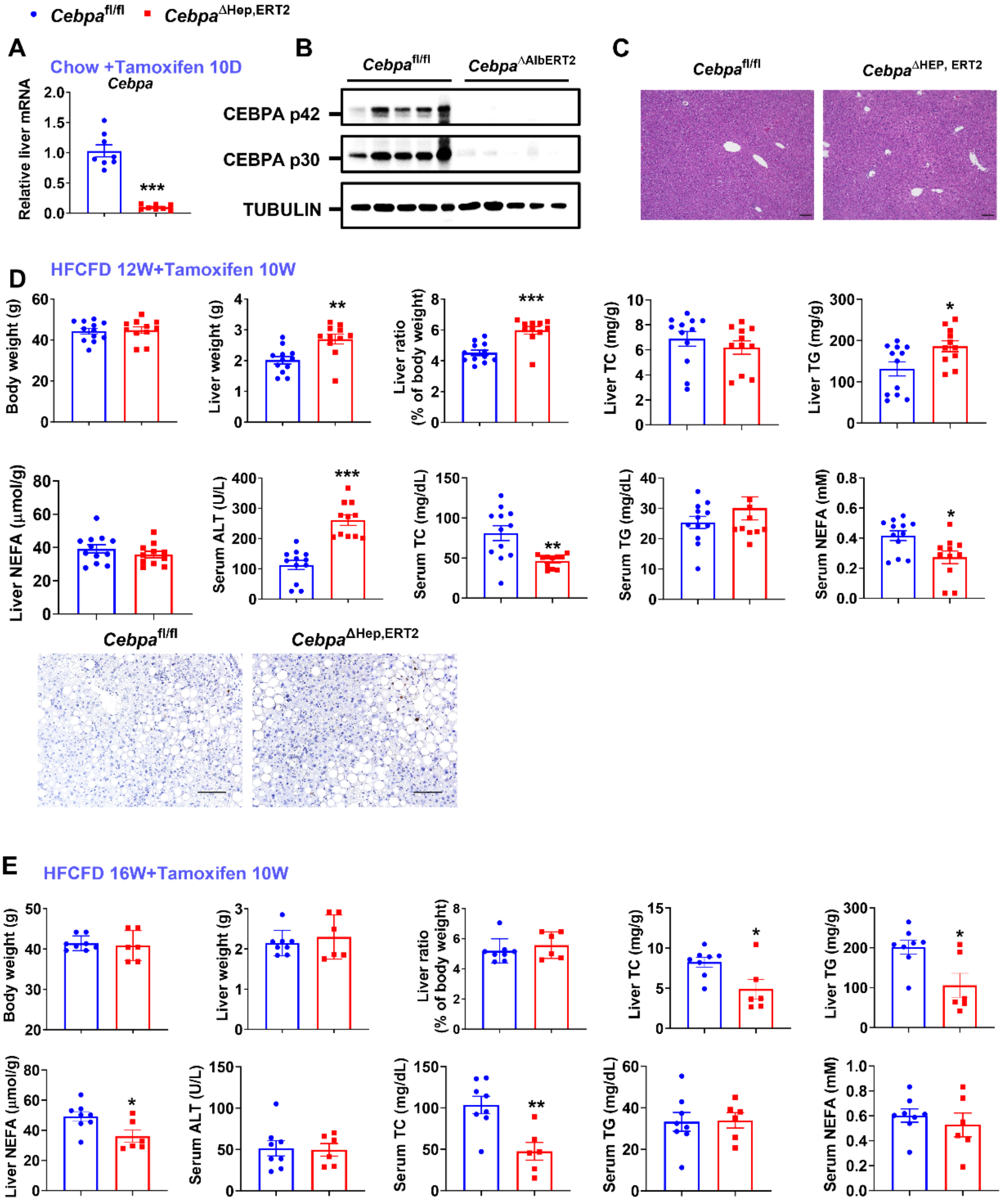

Figure S4. Biochemical parameters of chow or HFCFD-fed ***Cebpa*$^{\Delta Hep,ERT2}$ mice.** (A-C) Hepatic *Cebpa* mRNA (A, *n* = 8), CEBPA protein (B, *n* = 5) and liver H&E staining (C, scale bar 100 μm) in tamoxifen-dosed chow diet-fed *Cebpa*$^{fl/fl}$ or *Cebpa*$^{\Delta Hep,ERT2}$ mice. The mice were

treated with tamoxifen at 50 mg/kg (dissolved in corn oil) via intraperitoneal injection once daily at day 0, day 1, day 2 and day 9, then euthanized at day 10. Scale bar, 100 μm. (D) Body weights, liver weights, liver weight ratios, liver TC, liver TG, liver NEFA, serum ALT, serum TC, serum TG, serum NEFA and liver TUNEL staining for 22-week HFCFD-fed *Cebpa*$^{fl/fl}$ or *Cebpa*$^{\Delta Hep,ERT2}$ mice that were treated with tamoxifen for the last 10 weeks (n = 12 for *Cebpa*$^{fl/fl}$ mice and n = 11 for *Cebpa*$^{\Delta Hep,ERT2}$ mice), scale bar 100 μm. (E) Body weights, liver weights, liver weight ratios, liver TC, liver TG, liver NEFA, serum ALT, serum TC, serum TG and serum NEFA for 26-week HFCFD-fed *Cebpa*$^{fl/fl}$ or *Cebpa*$^{\Delta Hep,ERT2}$ mice treated with tamoxifen for the last 10 weeks; n = 8 for *Cebpa*$^{fl/fl}$ mice or n = 6 for *Cebpa*$^{\Delta Hep,ERT2}$ mice. Data represent mean ± SEM. *$P < 0.05$, **$P < 0.01$, ***$P < 0.001$, by two-tailed unpaired student's t test.

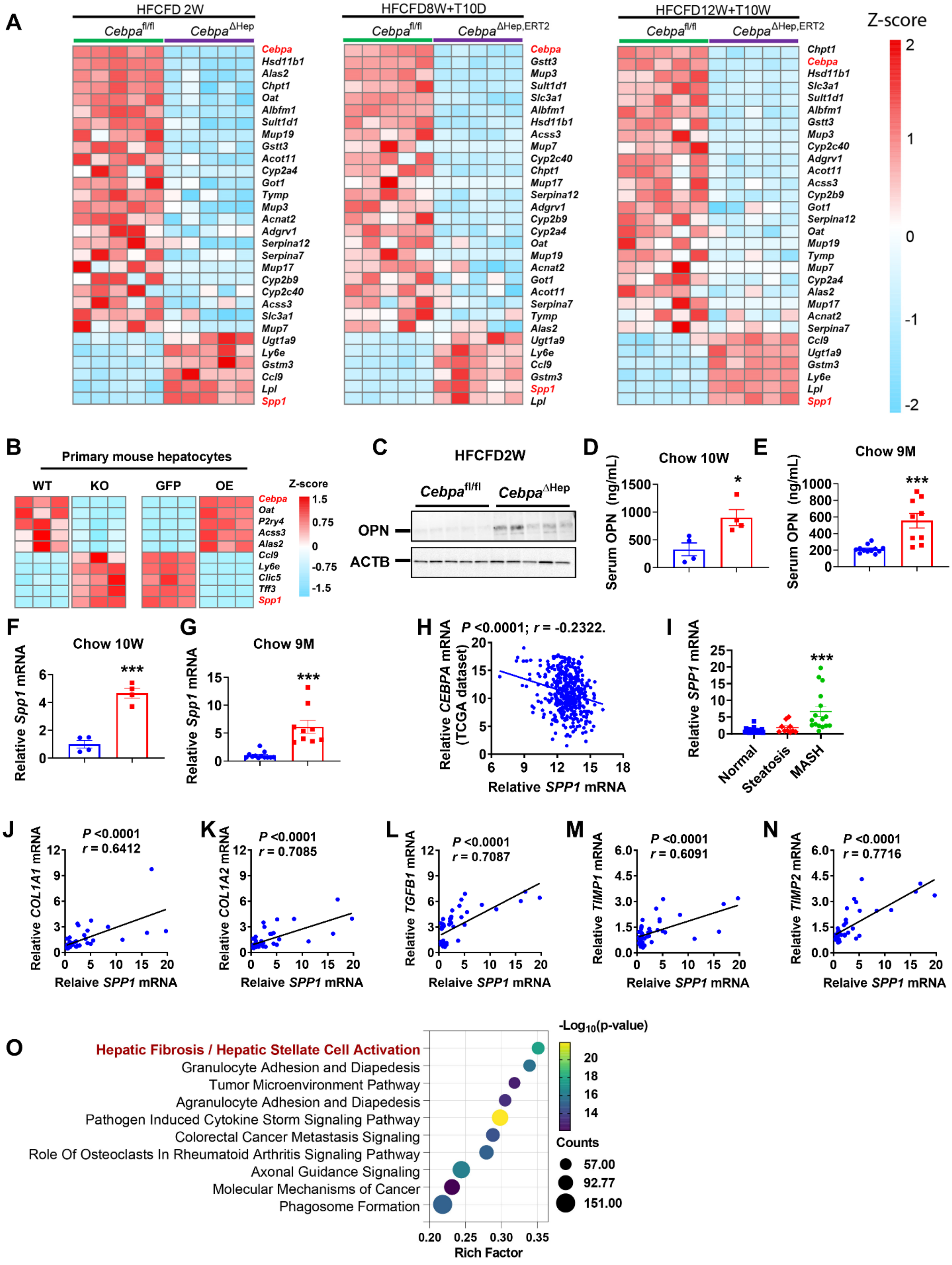
A
HFCFD 2W
HFCFD8W+T10D
HFCFD12W+T10W
Z-score
B
Primary mouse hepatocytes
WT
KO
GFP
OE
C
HFCFD2W
OPN
ACTB
D
Chow 10W
Serum OPN (ng/mL)
E
Chow 9M
F
Chow 10W
Relative Spp1 mRNA
G
Chow 9M
H
P <0.0001; r = -0.2322.
Relative CEBPA mRNA (TCGA dataset)
Relative SPP1 mRNA
I
Relative SPP1 mRNA
Normal
Steatosis
MASH
J
P <0.0001
r = 0.6412
Relative COL1A1 mRNA
Relaive SPP1 mRNA
K
P <0.0001
r = 0.7085
Relative COL1A2 mRNA
L
P <0.0001
r = 0.7087
Relative TGFB1 mRNA
M
P <0.0001
r = 0.6091
Relative TIMP1 mRNA
N
P <0.0001
r = 0.7716
Relative TIMP2 mRNA
O
Hepatic Fibrosis / Hepatic Stellate Cell Activation
Granulocyte Adhesion and Diapedesis
Tumor Microenvironment Pathway
Agranulocyte Adhesion and Diapedesis
Pathogen Induced Cytokine Storm Signaling Pathway
Colorectal Cancer Metastasis Signaling
Role Of Osteoclasts In Rheumatoid Arthritis Signaling Pathway
Axonal Guidance Signaling
Molecular Mechanisms of Cancer
Phagosome Formation
Rich Factor
-Log10(p-value)
Counts
57.00
92.77
151.00

Figure S5. Hepatocyte CEBPA represses *Spp1* expression and OPN release in mice. (A-B) Heatmap to show top-differentiated genes in mouse livers (A) and in primary hepatocytes (B). WT, primary hepatocytes from wild-type *Cebpa*$^{fl/fl}$ mice. KO, primary hepatocytes from hepatocyte *Cebpa* knockout (*Cebpa*$^{\Delta Hep}$) mice. GFP, primary hepatocytes from wild-type C57BL/6N mice treated with Ad-GFP for 48 h. OE, primary hepatocytes from wild-type C57BL/6N mice treated with Ad-CEBPA for 48 h. (C) Hepatic OPN protein in 2-week HFCFD-fed *Cebpa*$^{\Delta Hep}$ mice, n = 5. (D-G) Serum OPN (D-E) and hepatic *Spp1* mRNA (F-G) in 10-week-old chow-fed mice (n = 4) and 9-month chow-fed mice (n = 12 for *Cebpa*$^{fl/fl}$ mice or n = 9 for *Cebpa*$^{\Delta Hep}$ mice). (H) Correlation of hepatic *CEBPA* mRNA with *SPP1* mRNA in human TCGA database. (I-N) *SPP1* mRNA from normal, steatosis, MASH human livers in microarray E-MEXP-3291 (I) and its correlation with hepatic gene mRNAs for *COL1A1* (J), *COL1A2* (K), *TGFB1* (L), *TIMP1* (M), and *TIMP2* (N), respectively, by non-parametric Pearson's test. n = 19 for control normal, n = 10 for steatosis, and n = 16 for MASH human livers. (O) Ingenuity Pathway Analysis (IPA) for top 10 enrichment pathways in 22-week HFCFD-fed mice treated with tamoxifen for the last 10 weeks. *P < 0.05, **P < 0.01, ***P < 0.001 by two-tailed unpaired student's t test for **Figure S5D-G**, while one-way ANOVA with Dunnett's multiple-comparisons test for **Figure S5I** compared to control.

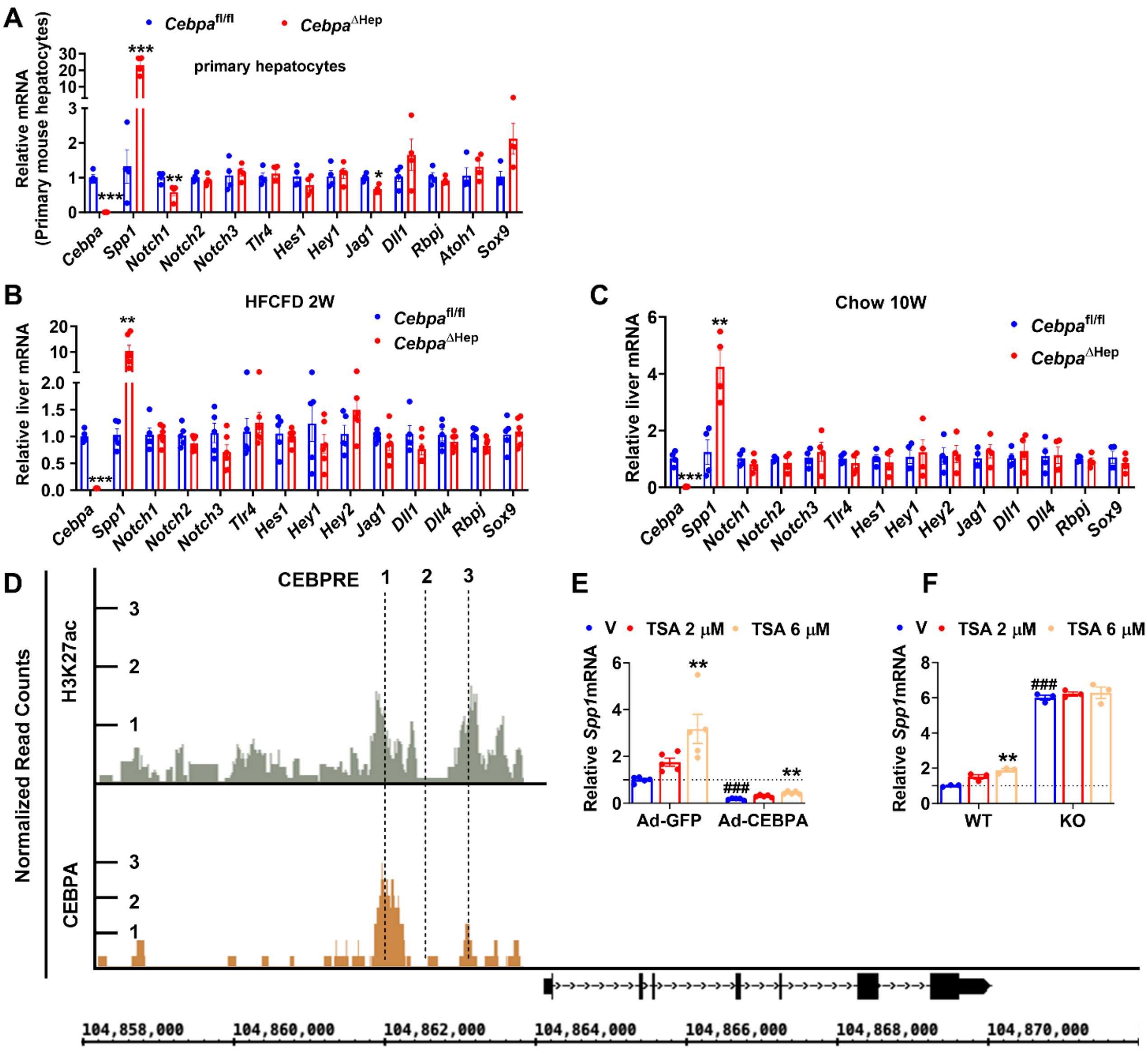

Figure S6. CEBPA represses *Spp1* expression and OPN release in primary hepatocytes in vitro without changing Notch signaling. (A-C) qPCR of genes involved in Notch signaling in primary hepatocytes isolated from chow-fed mice (A, $n = 4$), 2-week HFCFD-fed mice (B, $n = 5$ for *Cebpa*$^{fl/fl}$ mice and $n = 6$ for *Cebpa*$^{\Delta Hep}$ mice) and 10-week-old chow-fed mice (C, $n = 4$). (D) Bioinformatic analyses of ChIP-seq data for H3K27ac antibody (GSE60430) or CEBPA antibody (GSE65167) engagement. (E) *Spp1* mRNA in primary hepatocytes from 10-week-old chow-fed C57BL/6N mice treated with Ad-GFP or Ad-CEBPA in the presence of Trichostatin A (TSA) at 0, 2, 6 μM for 48 h ($n = 5$). (F) *Spp1* mRNA in primary hepatocytes from 10-week-old chow-fed *Cebpa*$^{fl/fl}$ (WT) or *Cebpa*$^{\Delta Hep}$ (KO) mice treated with Trichostatin A (TSA) at 0, 2 μM, 6 μM for 48 h ($n = 3$). Data represent mean ± SEM. *$P < 0.05$, **$P < 0.01$, ***$P < 0.001$ compared to each Vehicle (V) group. ###P<0.001 compared to Ad-GFP+V group in **Figure S6E** and compared to WT+V group in **Figure S6F**. Two-way ANOVA analyses with Tukey's multiple comparisons test were used for **Figure S6E-F**.

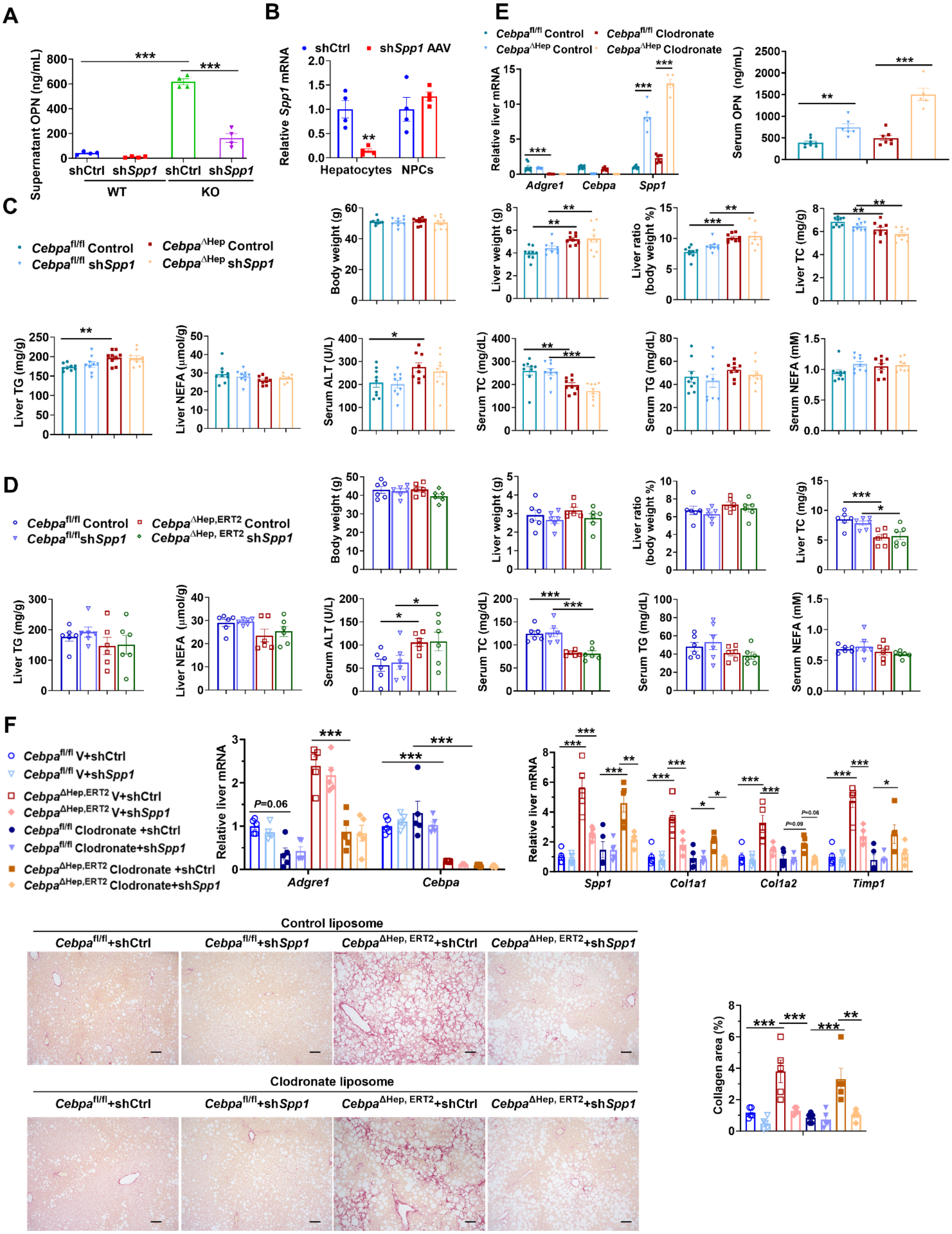

A
B
C
D
E
F
Control liposome
Clodronate liposome
Collagen area (%)

Figure S7. Biochemical parameters of AAV8-Sh*Spp1*-treated HFCFD-fed *Cebpa*$^{\Delta Hep}$ mice or *Cebpa*$^{\Delta Hep,ERT2}$ mice and examination of macrophage contribution to the present phenotype. (A) supernatant OPN levels (*n* = 4) of primary hepatocytes from wild-type *Cebpa*$^{fl/fl}$ or *Cebpa*$^{\Delta Hep}$ mice treated with scrambled or *Spp1* shRNA for 48 h in the presence of 0.4 mM palmitic acid. WT, wild-type. KO, knockout. shCtrl, scrambled shRNA. sh*Spp1* (*Spp1* shRNA). (B) *Spp1* mRNA in primary hepatocytes or nonparenchymal cells (NPCs) from AAV8-control scrambled shRNA (shCtrl) or AAV8-*Spp1* shRNA (sh*Spp1*)-treated mice (*n* = 4). (C) Body weights, liver weights, liver weight ratios, liver TC, liver TG, and liver NEFA, serum ALT, serum TC, serum TG, and serum NEFA in 16-week HFCFD-fed mice that were treated with AAV8-control scrambled shRNA (control) or AAV8-*Spp1* shRNA (sh*Spp1*) (*n* = 9). (D) Body weights, liver weights, liver weight ratios, liver TC, liver TG and liver NEFA, serum ALT, serum TC, serum TG and serum NEFA in 24-week HFCFD-fed mice that were treated with tamoxifen for the last 12 weeks, with AAV8-control scrambled shRNA (control) or AAV8-*Spp1* shRNA (sh*Spp1*) treated once (i.v.) at 1 week prior to tamoxifen dosing (*n* = 6). (E) Relative mRNA for macrophage marker *Adgre1* and *Spp1* mRNA (E left) and serum OPN levels (E right) in 2-week clodronate-treated mice (*n* = 5-8). (F) Relative liver mRNA for genes *Adgre1*, *Spp1*, *Col1a1*, *Col1a2* and *Timp1* (F upper) as well as Sirius red staining and quantitation of livers from 4-week clodronate-treated mice (F bottom), *n* = 5; scale bar 100 µm. Data represent mean ± SEM. **P* < 0.05, ***P* < 0.01, ****P* < 0.001 by two-tailed unpaired student's *t* test for **Figure S7B**, two-way ANOVA with Šidák's post hoc test for **Figure S7A, C, D, E,** and three-way ANOVA with Šídák's multiple comparisons test for **Figure S7F**.

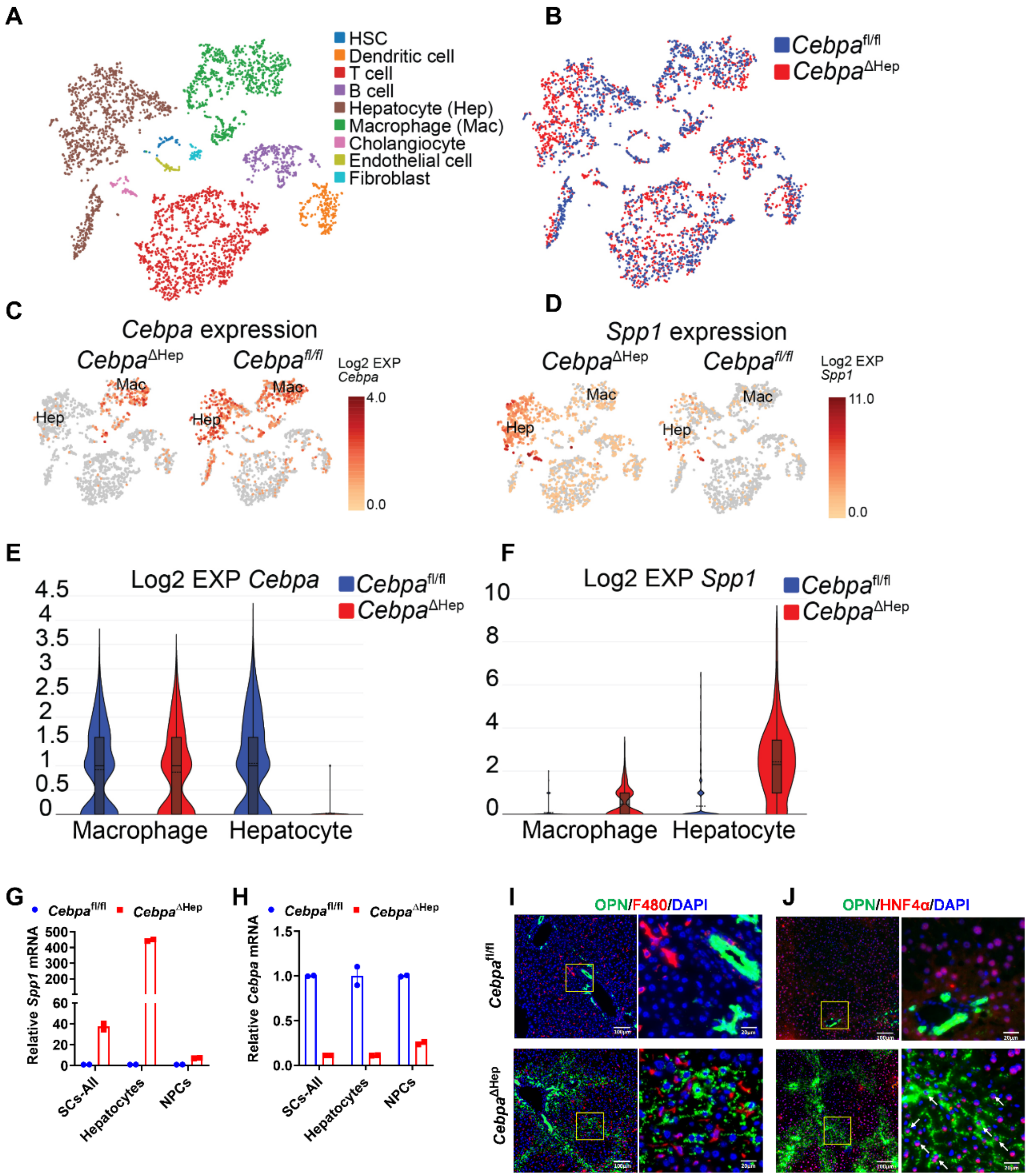

Figure S8. Hepatocyte CEBPA loss induced *Spp1* expression mainly in hepatocytes by scRNA-seq analyses and immunofluorescence staining. (A-B) The tSNE plots showing the identified cell cluster based on different cell types (A) and single cell cluster from liver of *Cebpa*$^{fl/fl}$ and *Cebpa*$^{\Delta Hep}$ mice (B), *n* = 1. (C-D) The tSNE plots showing the expression levels of *Cebpa* (C) and *Spp1* (D) among different cell populations (Hep, hepatocytes; Mac, macrophage), *n* = 1.

(E-F) The violin plots showing the Log2 expression of *Cebpa* (E) and *Spp1* (F) in hepatocyte and macrophage of *Cebpa*$^{fl/fl}$ and *Cebpa*$^{\Delta Hep}$ mice, *n* = 1. (G-H) The *Spp1* (G) and *Cebpa* (H) mRNA expression in all mixed single cell populations (SCs-All), enriched hepatocytes, and enriched nonparenchymal cells (NPCs), *n* = 2. Data represent mean ± SEM. (I-J) Representative OPN expression in macrophage (I, F4/80 as macrophage marker) and hepatocytes (J, HNF4α as a hepatocyte marker) of livers from 2-week HFCFD-fed *Cebpa*$^{fl/fl}$ and *Cebpa*$^{\Delta Hep}$ mice. Scale bar 100 μm for I left and J left, and scale bar 20 μm for I right and J right.

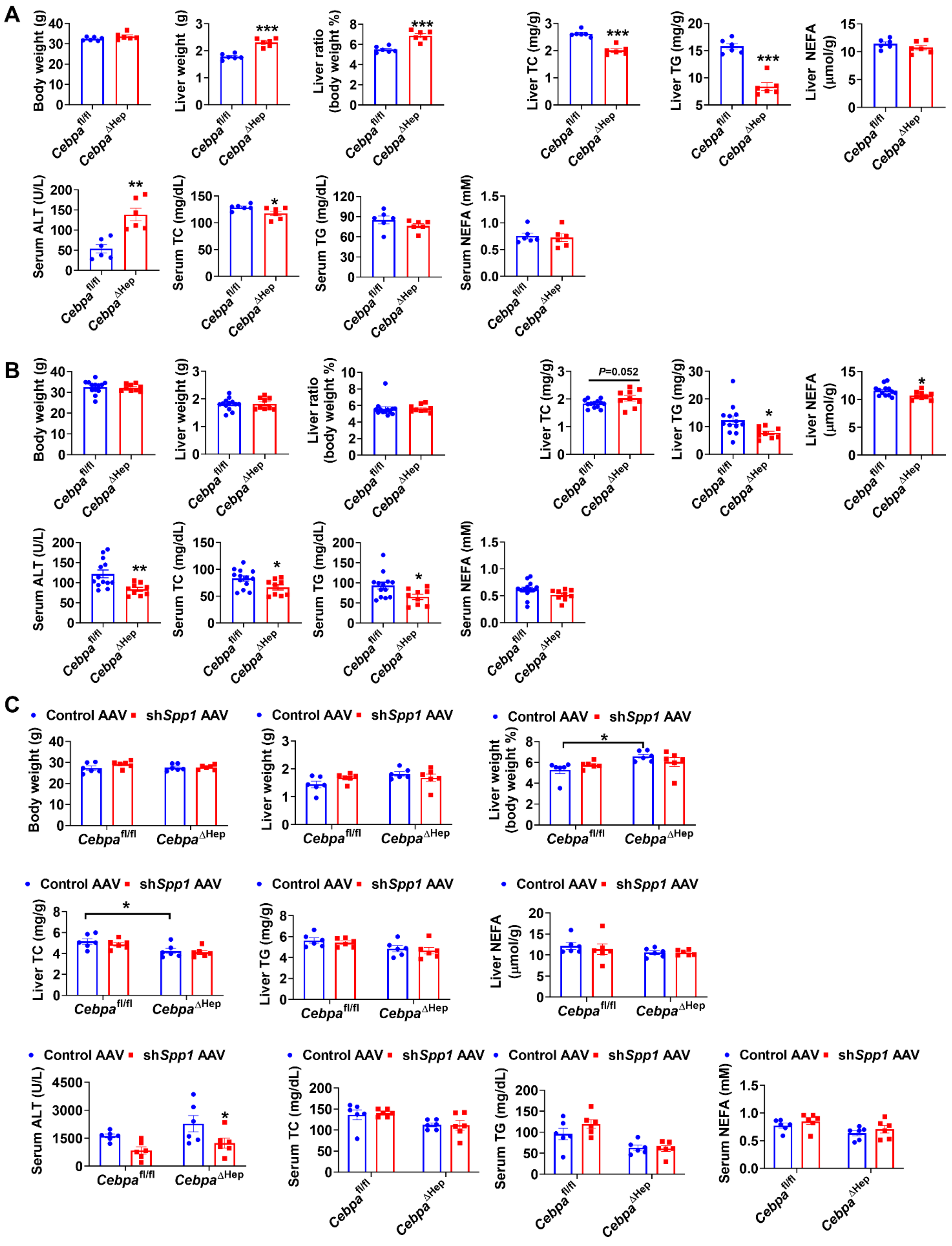
A
Body weight (g)
Liver weight (g)
Liver ratio (body weight %)
Liver TC (mg/g)
Liver TG (mg/g)
Liver NEFA (μmol/g)
Serum ALT (U/L)
Serum TC (mg/dL)
Serum TG (mg/dL)
Serum NEFA (mM)
Cebpa fl/fl
Cebpa ΔHep
B
P=0.052
C
Control AAV
shSpp1 AAV

Figure S9. Body weights, liver weights and biochemical parameters of CCl_4-treated mice. (A) Body weights, liver weights, liver weight ratios, liver TC, liver TG, liver NEFA, serum ALT, serum TC, serum TG and serum NEFA in 4-week CCl_4-treated mice, *n* = 6. (B) Body weights, liver weights, liver weight ratios, liver TC, liver TG, liver NEFA, serum ALT, serum TC, serum TG and serum NEFA in 8-week CCl_4-treated mice, *n* = 13 for *Cebpa*$^{fl/fl}$ mice or *n* = 9 for *Cebpa*$^{\Delta Hep}$ mice. (C) Body weights, liver weights, liver weight ratios, liver TC, liver TG, liver NEFA, serum ALT, serum TC, serum TG and serum NEFA, *n* = 6. The 4-week CCl_4-treated mice were dosed with AAV8-control scrambled shRNA (control) or AAV8-*Spp1* shRNA (sh*Spp1*) once (i.v.) at 1 week prior to CCl_4 dosing. Data represent mean ± SEM. **P*<0.05, ***P* < 0.01, ****P* < 0.001 by two-tailed unpaired student's test for A-B or by two-way ANOVA with Šídák's multiple comparisons test for C.

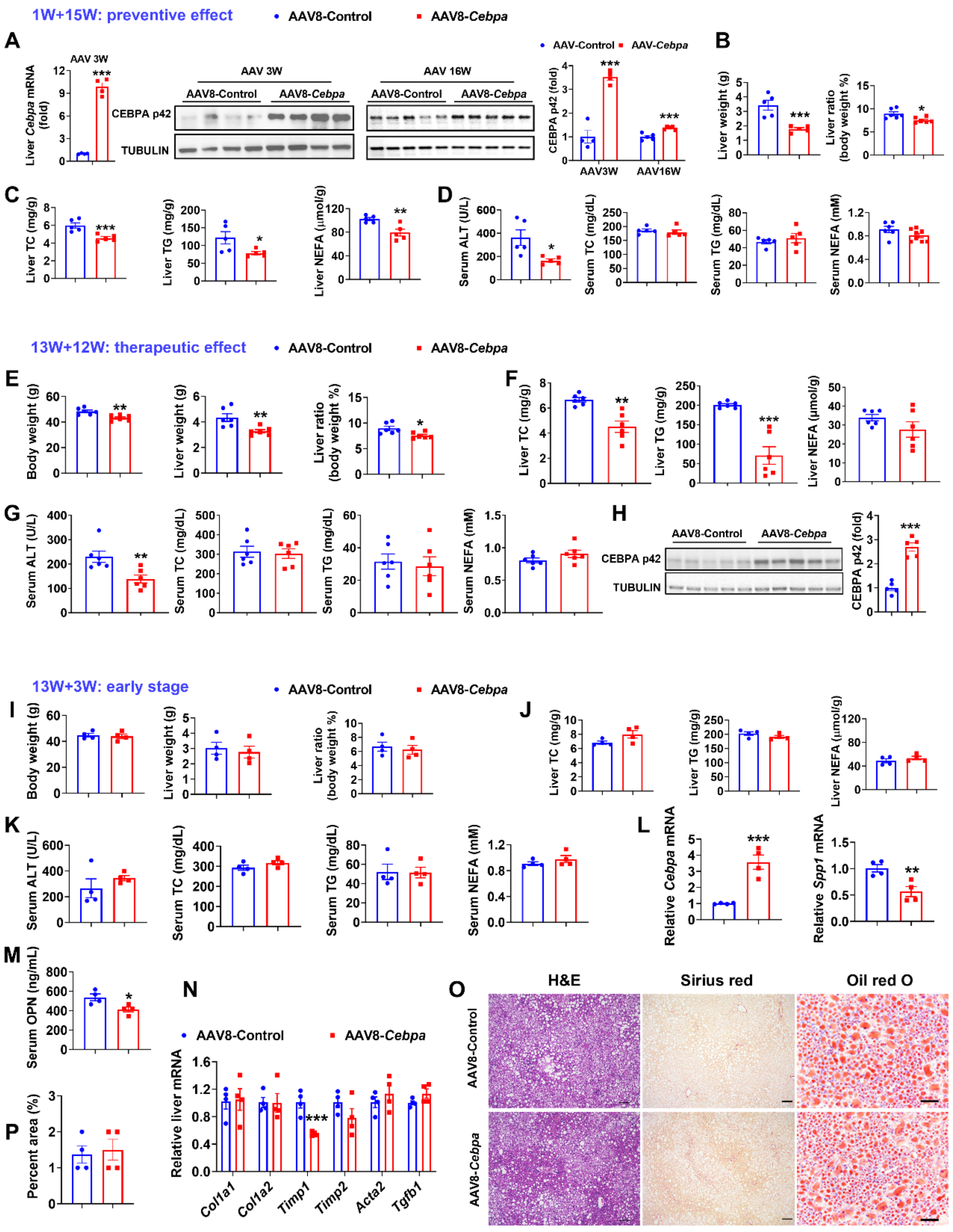
1W+15W: preventive effect
AAV8-Control
AAV8-Cebpa
A
B
C
D
13W+12W: therapeutic effect
E
F
G
H
13W+3W: early stage
I
J
K
L
M
N
O
P
H&E
Sirius red
Oil red O

Figure S10. Effects of AAV8-TBG-*Cebpa* on CEBPA expression, biochemical parameters, *Spp1* expression and serum OPN after both preventive and therapeutic treatment as well as at 3 weeks after AAV8 treatment in HFCFD-fed mice. (A) Liver *Cebpa* mRNA, CEBPA p42 protein and quantitation (*n* = 4 for 3-week AAV-treated mice and *n* = 5 for 16-week AAV-treated mice). (B-D) Liver weights, liver weight ratios, and serum ALT (B), liver TC, TG, NEFA (C), and serum TC, TG, NEFA (D) of the HFCFD-fed C57BL/6N mice treated with AAV8-Control and AAV8-*Cebpa* in a preventive manner, *n* = 5. (E-H) Body weights, liver weights, liver weight ratios (E), liver TC, TG, NEFA (F), serum ALT, TC, TG, NEFA (G) and CEBPA p42 protein levels (H, *n* = 5) of HFCFD-fed mice treated with AAV8-Control and AAV8-*Cebpa* in a therapeutic manner, *n* = 6 unless otherwise stated. (I-K) Body weights, liver weights, liver weight ratios (I), liver TC, TG, NEFA (J), and serum ALT, TC, TG, NEFA (K) of 16-week HFCFD-fed C57BL/6N mice treated with AAV8-Control and AAV8-*Cebpa* for the final 3 weeks, *n* = 4. (L-N) Hepatic *Cebpa* and *Spp1* mRNA (L), serum OPN (M), and liver gene mRNA in liver fibrosis (N) in 16-week HFCFD-fed C57BL/6N mice treated with AAV8-Control and AAV8-*Cebpa* for the final 3 weeks, *n* = 4. (O-P) Representative histological staining (O) and quantitation of Sirius red staining (P, *n* = 4) in the livers of 16-week HFCFD-fed C57BL/6N mice treated with AAV8-Control and AAV8-*Cebpa* for the final 3 weeks. Data represent mean ± SEM. **P* < 0.05, ***P* < 0.01, ****P* < 0.001, by two-tailed unpaired student's *t* test. Scale bar 100 μm for H&E staining and Sirius red staining, while scale bar 50 μm for Oil red O staining.

Supplemental Tables

Table S1. Predicted *Cebpa* promoter-binding transcriptional factors.

Ahr	*Dlx4*	*Foxo3*	*Id2*	*Mycn*	*Pdx1*	*Sp1*	*Twist2*
Arid3a	*Dux*	*Gabpa*	*Irf4*	*Myod1*	*Phox2a*	*Sp4*	*Zbtb7b*
Arid3b	*E2f3*	*Gata1*	*Irf6*	*Myog*	*Pitx1*	*Spi1*	*Zfp105*
Arid5a	*Ebf1*	*Gata4*	*Irx5*	*Neurog1*	*Plagl1*	*Srf*	*Zfp128*
Arnt	*Egr1*	*Gata5*	*Isgf3g*	*Nfatc2*	*Pou2f1*	*Sry*	*Zfp187*
Arntl	*Egr2*	*Gfi1b*	*Jun*	*Nfkb1*	*Prrx2*	*Stat3*	*Zfp281*
Ascl2	*Elf3*	*Gmeb1*	*Klf1*	*Nkx2-5*	*Rfx4*	*Stat4*	*Zfp410*
Atf3	*Elf5*	*Hand1*	*Klf12*	*Nkx3-1*	*Rfxdc2*	*Stat5a*	*Zfp740*
Atoh1	*En1*	***Hes1***	*Klf4*	*Nkx3-2*	*Runx1*	*Stat6*	*Zfx*
Barhl1	*Erg*	*Hes2*	*Klf7*	*Nobox*	*Rxra*	*T*	*Zic1*
Barx2	*Esrra*	*Hic1*	*Lhx4*	*Npas2*	*Sfpi1*	*Tbp*	*Zic2*
Bcl6b	*Esrrb*	*Hnf4a*	*Lhx8*	*Nr2e1*	*Shox2*	*Tcf1*	*Zic3*
Bhlha15	*Esrrg*	*Hoxa3*	*Max*	*Nr2e3*	*Six6*	*Tcf12*	*Zscan4*
Bhlhe40	*Esx1*	*Hoxa5*	*Meis1*	*NR3C1*	*Sox2*	*Tcf3*	
Cebpa	*Ets1*	*Hoxa7*	*Mitf*	*NR4A2*	*Sox3*	*Tcf7*	
Creb1	*Fos*	*Hoxa9*	*Mlxip*	*Nr5a2*	*Sox4*	*Tcf7l2*	
Creb3l2	*Foxa2*	*Hoxb7*	*Mnx1*	*Obox6*	*Sox5*	*Tcfap2a*	
Crx	*Foxj1*	*Hoxb8*	*Msx1*	*Osr1*	*Sox6*	*Tcfap2c*	
Dbx2	*Foxj2*	*Hoxc9*	*Msx3*	*Osr2*	*Sox10*	*Tcfap2e*	
Dlx1	*Foxj3*	*Hoxd9*	*Mtf1*	*Pax2*	*Sox11*	*Tcfcp2l1*	
Dlx2	*Foxl1*	*Hoxd10*	*Myb*	*Pax6*	*Sox14*	*Tcfe2a*	
Dlx3	*Foxo1*	*Hoxd13*	*Myc*	*Pax7*	*Sox17*	*Tcfl5*	

Table S2. Human patient information

Characteristics	Normal	F0	F1	F2	F3	F4
**Gender, *n* (%)**						
**Male**	2(66.7）	5 (55.6)	4 (44.4)	2 (28.6)	1 (16.7)	2 (28.6)
**Female**	1(33.3)	4 (44.4)	5 (55.6)	5 (71.4)	5 (83.3)	5 (71.4)
**Age, *y***						
**Mean (SEM)**	58.3 (5.9)	30.9 (5.6)	45.2 (5.9)	45.0 (7.0)	55.0 (4.7)	54.7 (3.4)
**Range**	51.0-70.0	17.0-55.0	15.0-66.0	28.0-71.0	33.0-66.0	36.0-62.0

Table S3. Gene markers of each cell type used for cluster identification

Cell types	Markers
Macrophage	*Adgre1 (F4/80)*
Hepatocyte	*Hnf4a*
B cells	*Cd79a, Cd79b*
T cells	*Cd3g, Cd3d*
Cholangiocytes	*Tm4sf4, Epcam*
Dendritic cells	*Siglech*
Endothelial cells	*Aqp1*
HSCs	*Col3a1*
Fibroblast	*Bmp2, Kdr*

Table S4. Primer list

Mouse qPCR	Forward sequence	Reverse sequence
Cebpa	CAAGAACAGCAACGAGTACCG	GTCACTGGTCAACTCCAGCAC
Atf3	GAGGATTTTGCTAACCTGACACC	TTGACGGTAACTGACTCCAGC
Spp1	CCTGGCTGAATTCTGAGGGAC	CAGTCACTTTCACCGGGAGG
Col1a1	TAGGCCATTGTGTATGCAGC	ACATGTTCAGCTTTGTGGACC
Col1a2	AGCAGGTCCTTGGAAACCTT	AAGGAGTTTCATCTGGCCCT
Timp1	CATGGAAAGCCTCTGTGGATATG	GATGTGCAAATTTCCGTTCCTT
Timp2	ATGGTTCTTGCGCGTGGTA	GCTTTTCAATTGGCCAC GG
Acta2	CCAGCCATCTTTCATTGGGATG	TACCCCCTGACAGGACGTTG
Tgfb1	GTCACTGGAGTTGTACGGCA	GGGCTGATCCCGTTGATTTC
Mmp2	TTTGCTCGGGCCTTAAAAGTAT	CCATCAAACGGGTATCCATCT C
Mmp13	GGTCCTTGGAGTGATCCAGA	TGATGAAACCTGGACAAGCA
Tnfa	CCACCACGCTCTTCTGTCTAC	AGGGTCTGGGCCATAGAACT
Il6	TAGTCCTTCCTACCCCAATTTCC	TTGGTCCTTAGCCACTCCTTC
Il1b	GCCACCTTTTGACAGTGATGAG	GACAGCCCAGGTCAAAGGTT
Ccl2	TTAAAAACCTGGATCGGAACCAA	GCATTAGCTTCAGATTTACGGGT
Srebp1c	GGAGCCATGGATTGCACATT	GCTTCCAGAGAGGAGGCCAG
Scd1	TTCTTGCGATACACTCTGGTGC	CGGGATTGAATGTTCTTGTCGT
Fasn	AAGTTGCCCGAGTCAGAGAACC	ATCCATAGAGCCCAGCCTTCCAT
Dgat1	GACGGCTACTGGGATCTGA	TCACCACACACCAATTCAGG
Dgat2	CGCAGCGAAAACAAGAATAA	GAAGATGTCTTGGAGGGCTG
Cd36	AGATGACGTGGCAAAGAACAG	CCTTGGCTAGATAACGAACTCTG
Fabp1	ATGAACTTCTCCGGCAAGTACC	CTGACACCCCCTTGATGTCC
Fabp2	GTGGAAAGTAGACCGGAACGA	CCATCCTGTGTGATTGTCAGTT
Fabp3	GTGGAAAGTAGACCGGAACGA	CCATCCTGTGTGATTGTCAGTT
Fabp4	AAGGTGAAGAGCATCATAACCCT	TCACGCCTTTCATAACACATTCC
Fabp5	GGCAACAACATCACGGTCAA	GGTGCAGACCGTCTCAGTTT
Fatp1	TGAAGGGACCCACCAGCTA	AGCCCGCATCAGATCCCT
Fatp2	TCCTCCAAGATGTGCGGTACT	TAGGTGAGCGTCTCGTCTCG
Fatp3	CTGGCCACAGCTGGGTTT	ATTAGTTTCAGGGCCCGTCG
Fatp4	TCCCATCAGCAACTGTGACC	GGACGCTAGGGCTCTGAATC
Fatp5	CTACGCTGGCTGCATATAGATG	CCACAAAGGTCTCTGGAGGAT
Smpdl3b	CACACCGTGGAAGACCACAT	AGTAAGTCACCAAGTCCTTCAAGT
Plin2	AGCTCTCCTGTTAGGCGTCTC	AGGTTGGCCACTCTCATCAC
Plin4	TGACAACTGAGGAACAAGCTCA	TGTAGTAGCTTCCCCGGTCA
Plin5	CGCTCCATGAGTCAAGCCA	GCTGCCAGGACTGCTAGCTC
Acox1	GGGCACGGCTATTCTCACAG	CATCAAGAACCTGGCCGTCT

Acot1	CGATGACCTCCCCAAGAACAT	CTTTTACCTCGGGGTGGCT
Cpt1a	TGAGCCTGGCCTCGCC	CCATCTTGAGTGGTGACCGAG
Cpt1b	GAACACAAATGTGCAAGCAGC	GCCATGACCGGCTTGATCTC
Cpt2	CGTACCCACCATGCACTACC	TTCTGTCTTCCTGAACTGGCTG
Ehhadh	CGGTCAATGCCATCAGTCCAA	TGCTCCACAGATCACTATGGC
Acox2	ACGGTCCTGAACGCATTTATG	TTGGCCCCATTTAGCAATCTG
Acadl	TCTTTTCCTCGGAGCATGACA	GACCTCTCTACTCACTTCTCCAG
Acsl1	CGATGGCTGTTGGACTTTGC	CACCCAGGCTCGACTGTATC
Acaa1a	AGGCTTCAAGAACACCACCC	GGCTCCTGGCTCAAGAACAT
Fxr	TGGGCTCCGAATCCTCTTAGA	TGGTCCTCAAATAAGATCCTTGG
Cyp7a1	TTCTGCGAAGGCATTTGGAC	AGCATCTCCCTGGAGGGTTT
Cyp8b1	TTGCAAATGCTGCCTCAACC	TAACAGTCGCACACATGGCT
Shp	TCTGCAGGTCGTCCGACTATTC	AGGCAGTGGCTGTGAGATGC
Bsep	GCCAGTTCTGTTCTCCACCA	GTCAAATTGCTGTGGCAGGG
Notch1	CAGACCAACACGCAGTACCA	GACGTCAATGCCTCGCTTCT
Notch2	GCTATGGCCAACAGTAACCCT	GCGTAGCCCTTCAGACACTC
Notch3	TGTGCTACAGCCGTGTGTTT	CACAAGAGGCCTGTCTTCCC-
Tlr4	TGACACCAGGAAGCTTGAATCCCT	GGAATGTCATCAGGGACTTTGCTG
Hes1	AGCTTGGCTGTGGTAGAAGC	CGGTATTTCCCCAACACGCT
Hey1	CGTGGGAAAGGGATGGTTGA	CAAGTTTCCATTCTCGTCCGC
Hey2	GCTACAGGGGGTAAAGGCTAC	CAAGGCCTTCCACTGAGCTT
Jag1	ATACACGTGGCCATCTCTGC	CCGCTTCCTTACACACCAGT
Dll1	AGATAACCCTGACGGAGGCT	ACACACTTGGCACCGTTAGA
Dll4	TGCCTGGGAAGTATCCTCAC	GTGGCAATCACACACTCGTT
Rbpj	TTTCCACGCCAGTTCACAACA	TCTGCCCGTAATGGATGTAGC
Sox9	TCTGGAGGCTGCTGAACGAG	GCTTGTCCGTTCTTCACCGA
Actb	GGCTGTATTCCCCTCCATCG	CCAGTTGGTAACAATGCCATGT
Gapdh	AGGTCGGTGTGAACGGATTTG	TGTAGACCATGTAGTTGAGGTCA
**Human qPCR**		
CEBPA	TTCACATTGCACAAGGCACT	GAGGGACCGGAGTTATGACA
ATF3	AAGAACGAGAAGCAGCATTTGAT	TTCTGAGCCCGGACAATACAC
SPP1	TTGCAGCCTTCTCAGCCAAA	CAGCCTGTTTAACTGGTATGGCAC
COL1A1	CAGATCACGTCATCGCACAA	TGTGAGGCCACGCATGAG
COL1A2	CCAGAGTGGAGCAGTGGTTACTACT	TTCTTGGCTGGGATGTTTTCA
TIMP1	CTGTGAGGAATGCACAGTGTT	GGGACTGGAAGCCCTTTTCA
TIMP2	GCACATCACCCTCTGTGACTT	GGGCAGCGCGTGATCTTG
MMP2	CCCACTGCGGTTTTCTCGAAT	CAAAGGGGTATCCATCGCCAT
TGFB1	GGAAATTGAGGGCTTTCGCC	CCGGTAGTGAACCCGTTGAT
IL6	ACTCACCTCTTCAGAACGAATTG	CCATCTTTGGAAGGTTCAGGTTG

IL1B	ATGATGGCTTATTACAGTGGCAA	GTCGGAGATTCGTAGCTGGA
CCL2	AGGTGACTGGGGCATTGAT	GCCTCCAGCATGAAAGTCTC
ACTA2	CGGGACTAAGACGGGAATCCT	CAGAGCCCAGAGCCATTGTC
GAPDH	CCGCATCTTCTTTTGCGTCG	GCCCAATACGACCAAATCCGT
HES1	TGTCATCCCCGTCTACACCA	CACATGGAGTCCGCCGTAAG
ACTB	GAGACCGCGTCCGCC	ATCATCATCCATGGTGAGCTGG
**ChIP qPCR**		
P1	CAGTCCTGTCGCAATGGGAT	ATGCATCCTTAGAGGCCGAC
P2	TGCTTAAAGGGCAAAAACGCT	TAGTGCTACTTGCCAAACCCTG
P3	CTCCTTGCCTCTGCTGAAGATT	CTCCAGATAGAGCCCGCCTAA
P4	GGAAGTGTAGGAGCAGGTGG	GGTTTCCTCCGAGAATGCCT
RLP30	Primer purchased from Cell Signaling Technology, Cat #7014	
HOXD10	Primer purchased from Cell Signaling Technology, Cat #7429	
**Genotyping**		
Cebpa-flox	CCACTCACCGCCTTGGAAAGTCACA	Primer 1
Cebpa-flox_	CCGCGGCTCCACCTCGTAGAAGTCG	Primer 2
Cebpa-flox_	AGGGACCTAATAACTTCGTATAGCA	Primer 3
Cre forward	GATTTCGACCAGGTTCGTTC	Primer 1
Cre reverse	GCTAACCAGCGTTTTCGTTC	Primer 2

Table S5. Antibody list

Name	Citation	Supplier	Catalogue #; Antibody Registry
mouse SPP1, dilution 1:900	PMID:30463916	R&D Systems	Cat. # AF808; RRID: AB_2194992
ATF3, dilution 1:1000	PMID:25531328	Cell Signaling Technology	Cat. # 33593; RRID: AB_2799039
ACTB, dilution 1:1000	PMID:14702115	Cell Signaling Technology	Cat. # 4970S; RRID: AB_2223172
TUBLIN, dilution 1:1000	PMID:32191641	Cell Signaling Technology	Cat. # 2148S; RRID: AB_2288042
human SPP1, dilution 1:1000	Human Protein Atlas project	Sigma-Aldrich	Cat. # HPA027541; RRID: AB_10601446
CEBPA antibody for western blot, dilution 1:1000	PMID:30423293	Cell Signaling Technology	Cat. # 8178; RRID: AB_11178517
Goat anti-rabbit IgG-HRP, dilution 1:2000	PMID:35769880	Cell Signaling Technology	Cat. # 7074S; RRID: AB_2099233
Rabbit anti-goat IgG-HRP, dilution 1:2000	PMID:34907153	Abcam	Cat. # ab6741; RRID: AB_955424
IgG (ChIP)	PMID:32681035	Cell Signaling Technology	Cat. # 2729S; RRID: AB_1031062
H3K4me3 (ChIP)	PMID:29184203	Cell Signaling Technology	Cat. # 9751S; RRID: AB_2616028
H3K27ac (ChIP)	PMID:33534835	Abcam	Cat. # ab32369; RRID: AB_732780
F4/80 antibody	PMID:35304461	Cell Signaling Technology	Cat. # 70076; RRID: AB_2799771
OPN antibody	PMID: 36161100	Cell Signaling Technology	Cat. # 88742
HNF4α antibody	PMID: 30762896	Abcam	Cat. # 41898; RRID: AB_732976
Desmin antibody	PMID: 34294714	Abcam	Cat. # 15200; RRID: AB_301744
HES1 antibody	PMID: 35280902	Abclonal	Cat. # 11718; RRID: AB_2758714
Anti-FLAG antibody	PMID: 31320636	Abclonal	Cat. # AE005; RRID: AB_2770401
CEBPA antibody for IF and IHC	PMID: 32034145	Cell Signaling Technology	Cat. # 2295; RRID: AB_10692506
CD45 antibody for IHC	PMID:34890068	Abcam	Cat. # ab10558; RRID: AB_442810
HepPar1 antibody	PMID: 34105295	Novus Biologicals	Cat. # NBP2-45272
