# Supplementary material for: Hepatocyte-specific CCAAT/enhancer binding protein α restricts liver fibrosis progression
Source: J Clin Invest. 2024 Apr 1;134(7):e166731. doi: 10.1172/JCI166731 (PMC10977981; doi:10.1172/JCI166731)

Original images for Blots

Full western blot gel panels

(A) CEBPA p42 and p30 as well as TUBULIN from which the data in Figure 1E were derived.
(B) CEBPA p42 and p30 as well as TUBULIN from which the data in Figure 1L were derived.
(C) HES1 and TUBULIN from which the data in Figure S1M were derived.
(D) HES1 and ACTB from which the data in Figure S1N were derived.
(E) OPN and TUBULIN from which the data in Figure 4H were derived.
(F) CEBPA p42 and p30, OPN and TUBULIN from which the data in Figure 5B were derived.
(G) CEBPA p42 and p30, OPN and ACTB from which the data in Figure 5F were derived.
(H) CEBPA p42 and p30 as well as TUBULIN from which the data in Figure S2B were derived.
(I) CEBPA p42 and p30 as well as TUBULIN from which the data in Figure S4B were derived.
(J) OPN and ACTB from which the data in Figure S5C were derived.
(K) CEBPA p42 and TUBULIN from which the data in Figure S10A (left) were derived.
(L) CEBPA p42 and TUBULIN from which the data in Figure S10A (right) were derived.
(M) CEBPA p42 and TUBULIN from which the data in Figure S10F were derived.

Red encircled full blots, Purple encircled representative blots.

A

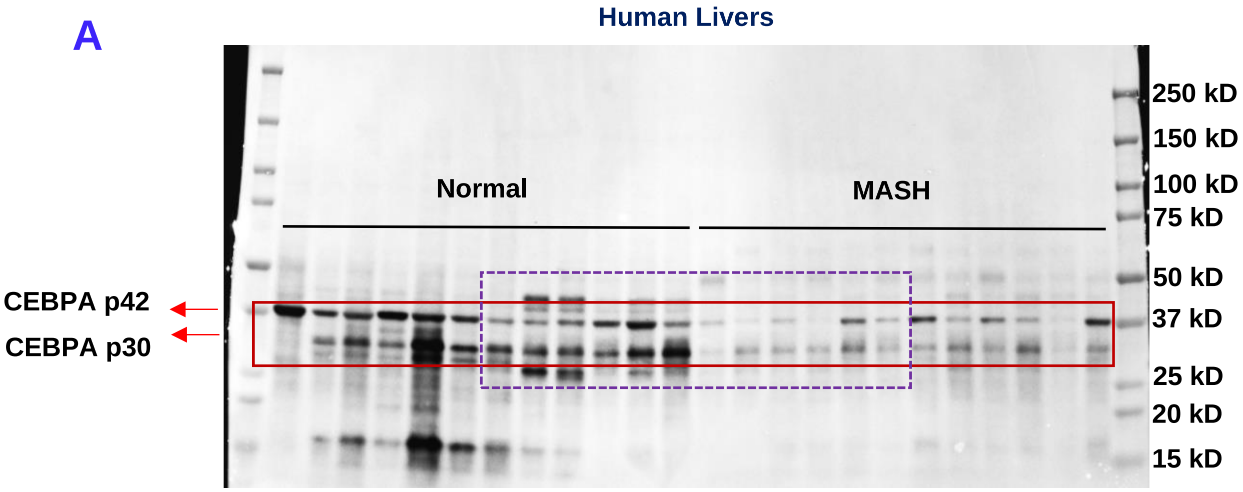

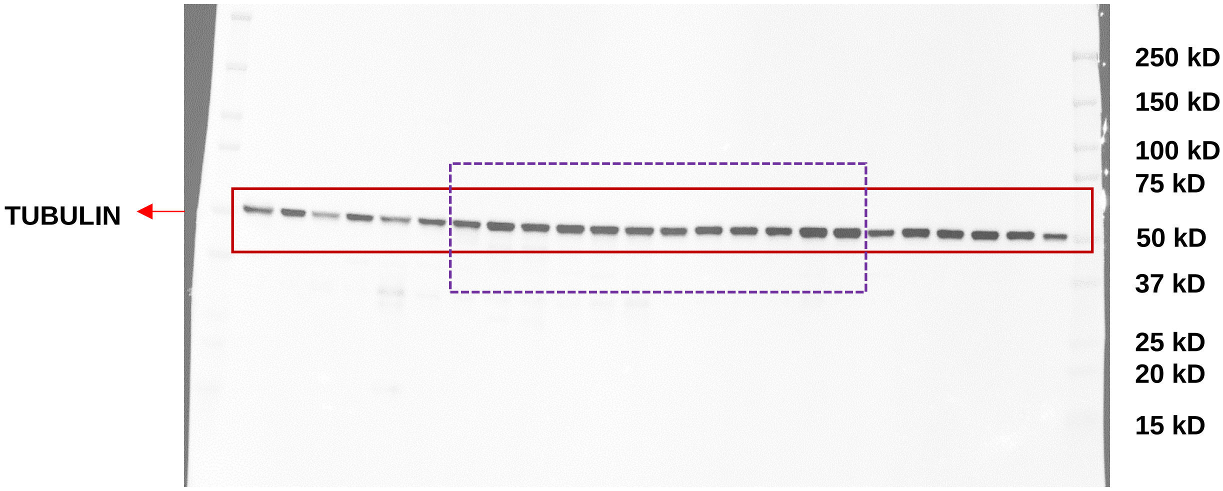

B

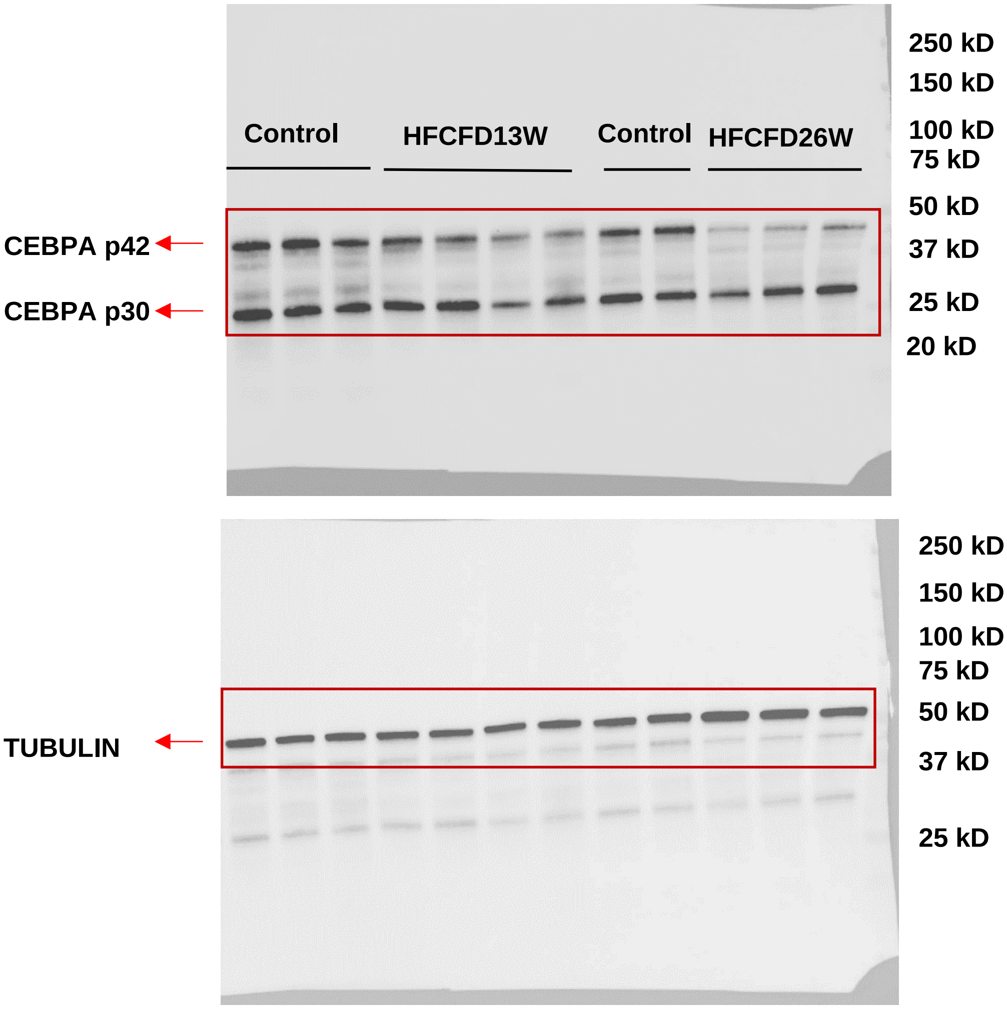
Control
HFCFD13W
Control
HFCFD26W
250 kD
150 kD
100 kD
75 kD
50 kD
37 kD
25 kD
20 kD
CEBPA p42
CEBPA p30
TUBULIN
250 kD
150 kD
100 kD
75 kD
50 kD
37 kD
25 kD

C

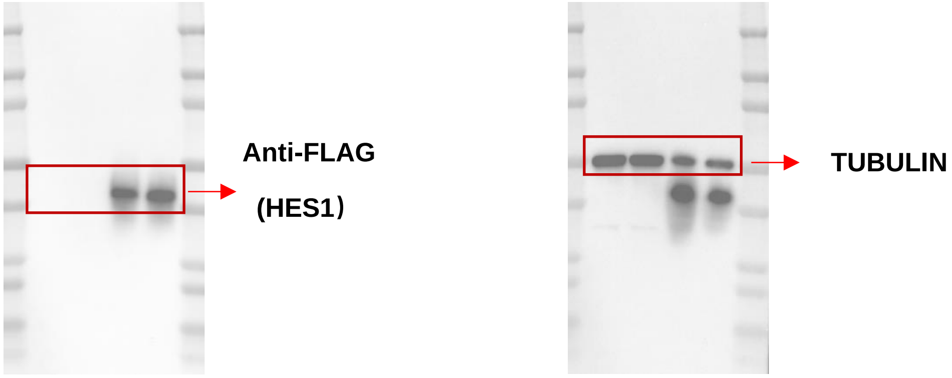

D

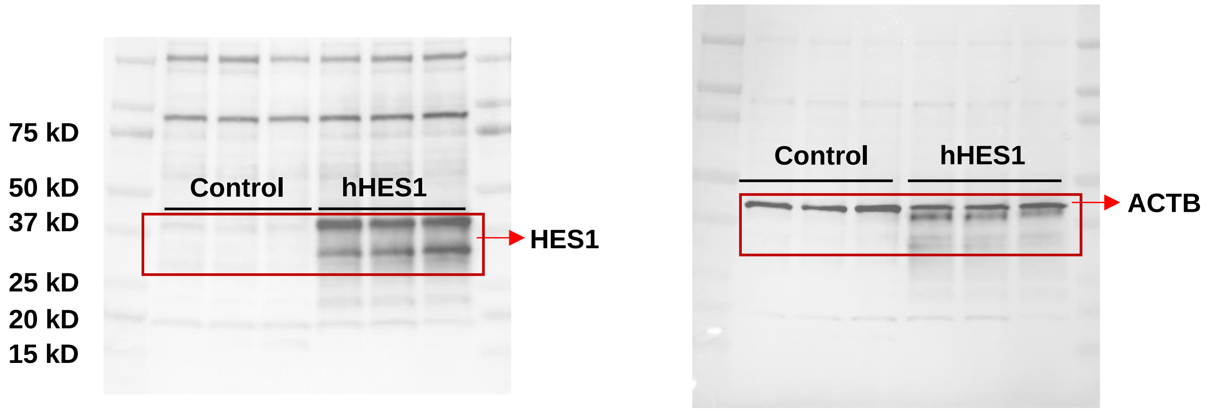
75 kD
50 kD
37 kD
25 kD
20 kD
15 kD
Control
hHES1
HES1
Control
hHES1
ACTB

Red encircled full blots, Purple encircled representative blots.

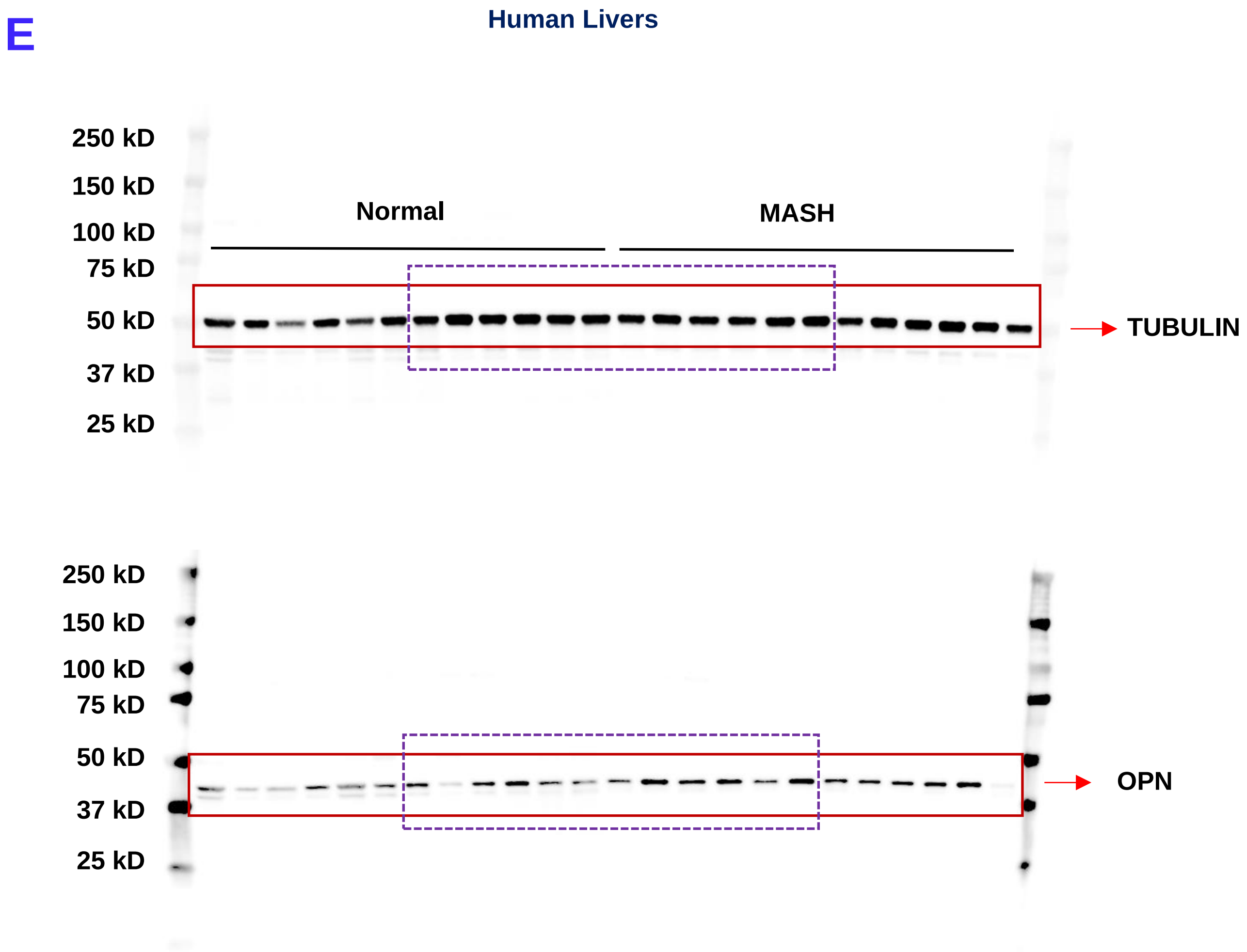

F

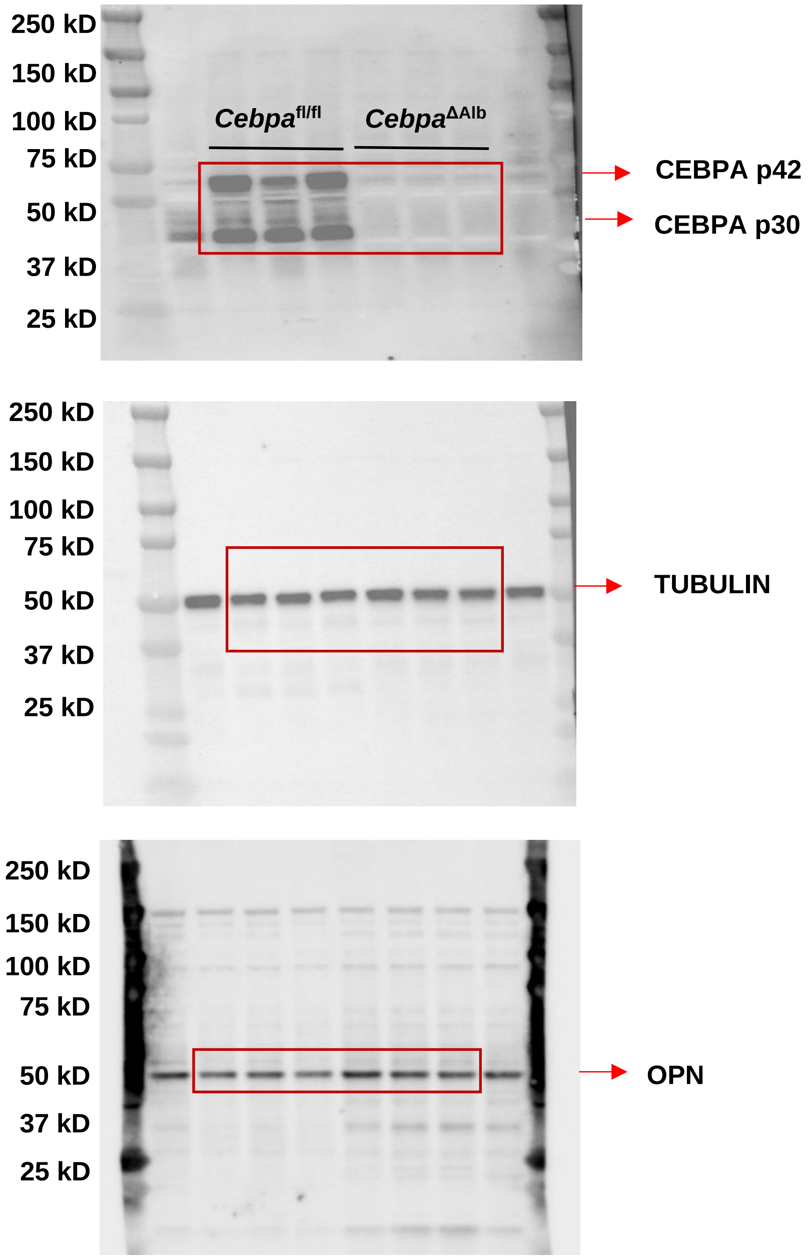
250 kD
150 kD
100 kD
75 kD
50 kD
37 kD
25 kD
$Cebpa^{fl/fl}$
$Cebpa^{\Delta Alb}$
CEBPA p42
CEBPA p30
250 kD
150 kD
100 kD
75 kD
50 kD
37 kD
25 kD
TUBULIN
250 kD
150 kD
100 kD
75 kD
50 kD
37 kD
25 kD
OPN

G

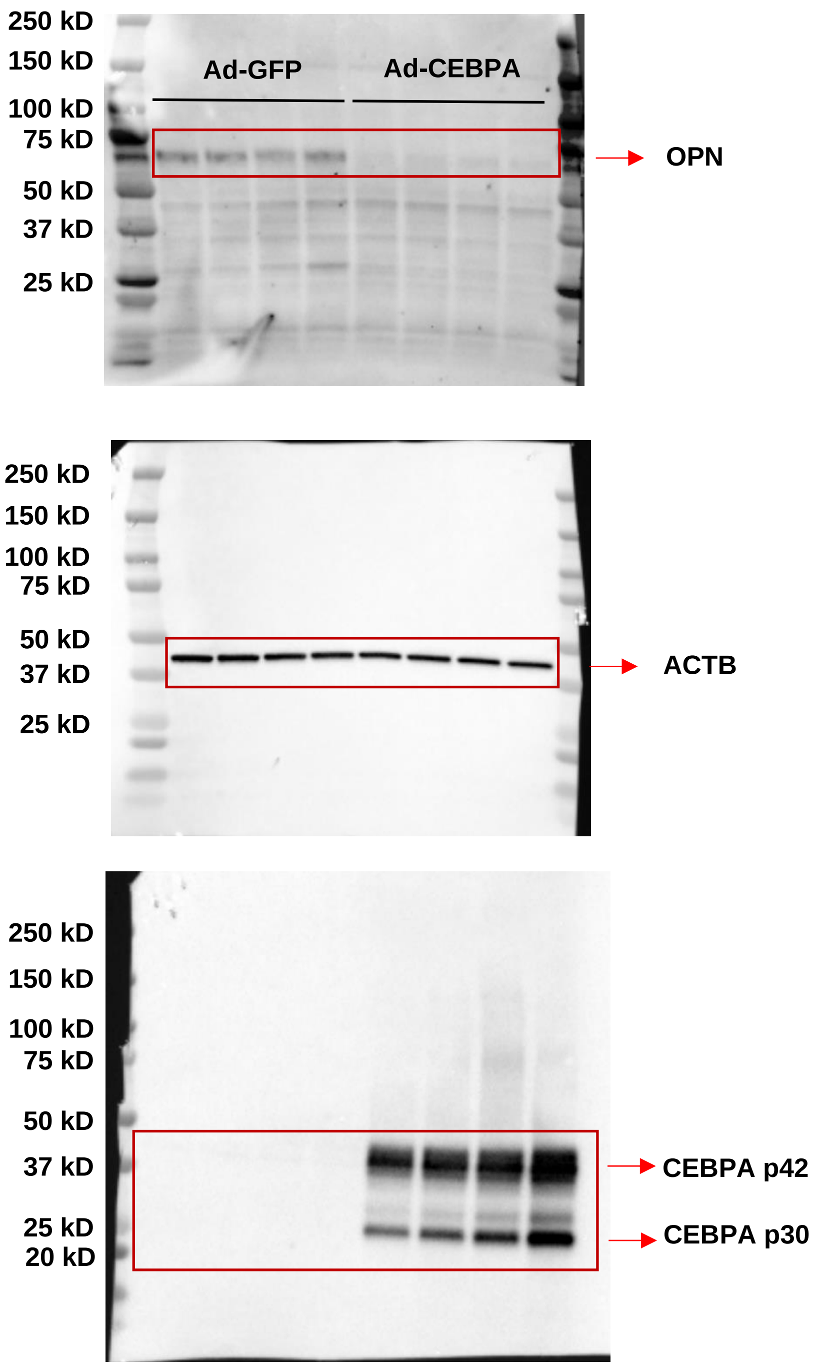
250 kD
150 kD
100 kD
75 kD
50 kD
37 kD
25 kD
Ad-GFP
Ad-CEBPA
OPN
250 kD
150 kD
100 kD
75 kD
50 kD
37 kD
25 kD
ACTB
250 kD
150 kD
100 kD
75 kD
50 kD
37 kD
25 kD
20 kD
CEBPA p42
CEBPA p30

H

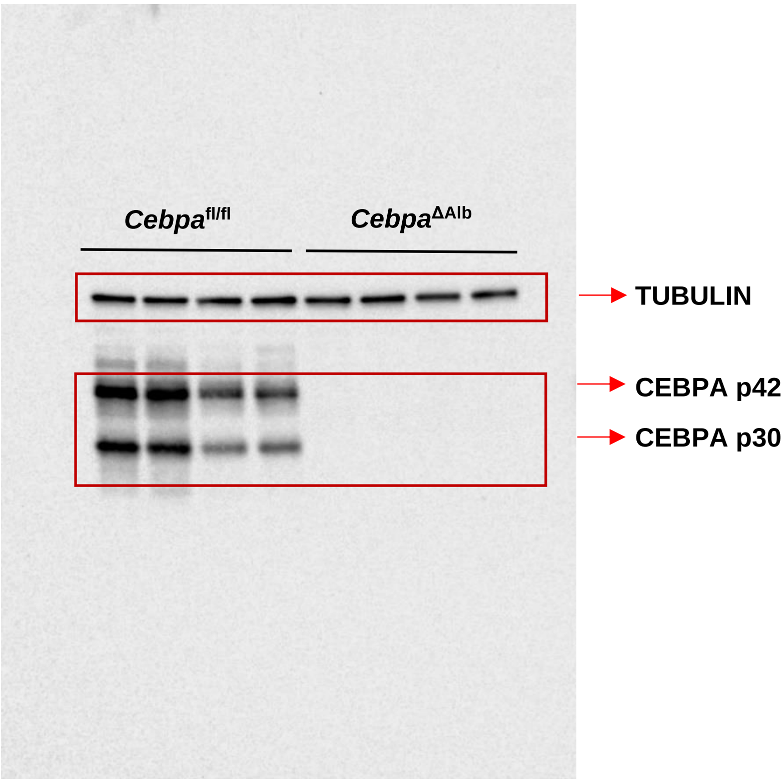

Cebpa fl/fl
Cebpa ΔAlb
TUBULIN
CEBPA p42
CEBPA p30

I

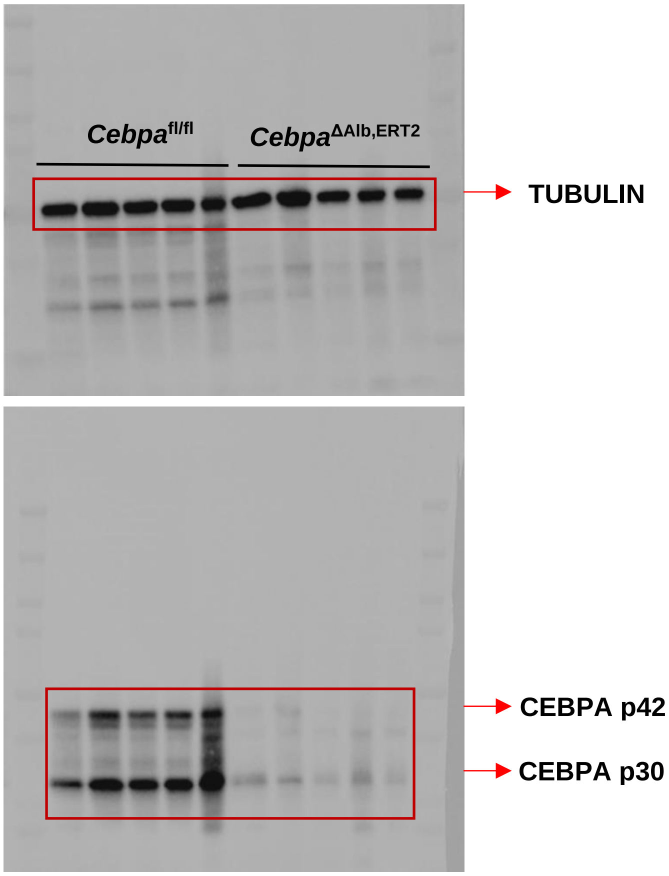

Cebpa fl/fl
Cebpa ΔAlb,ERT2
TUBULIN
CEBPA p42
CEBPA p30

J

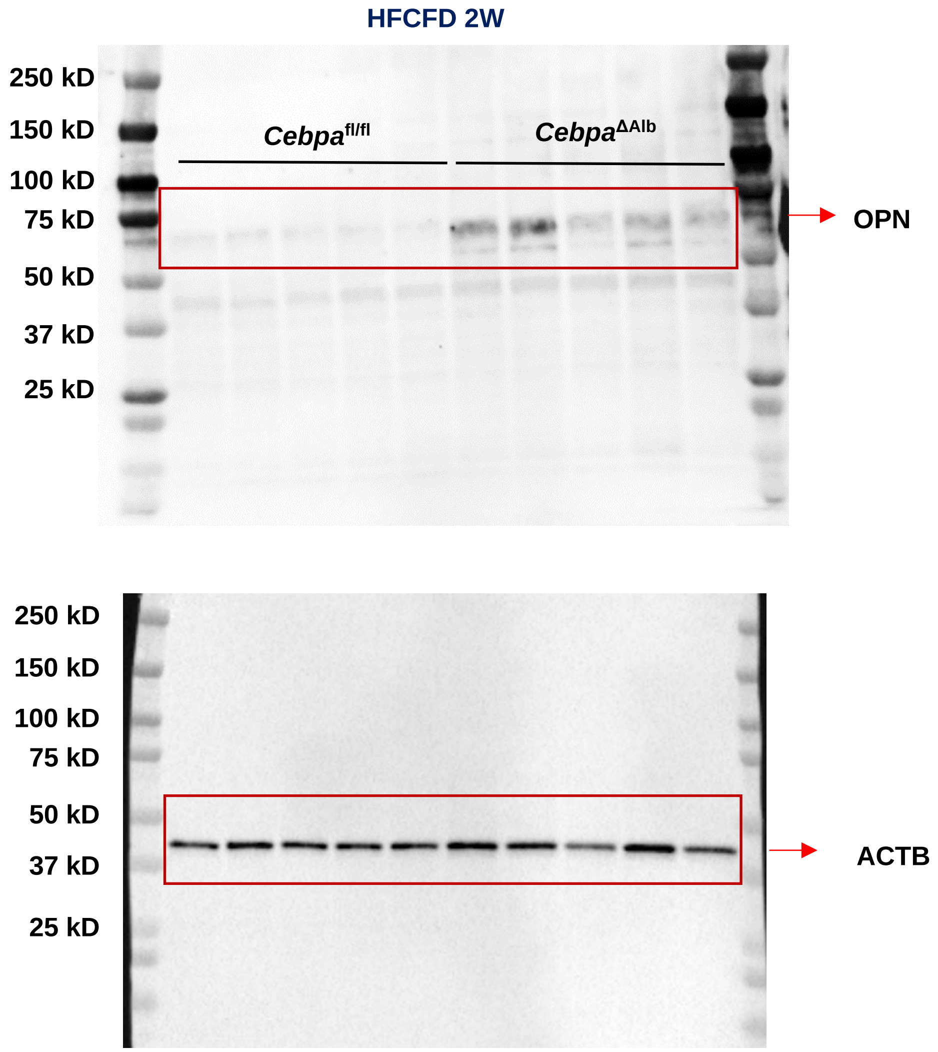

HFCFD 2W
250 kD
150 kD
100 kD
75 kD
50 kD
37 kD
25 kD
Cebpa fl/fl
Cebpa ΔAlb
OPN
250 kD
150 kD
100 kD
75 kD
50 kD
37 kD
25 kD
ACTB

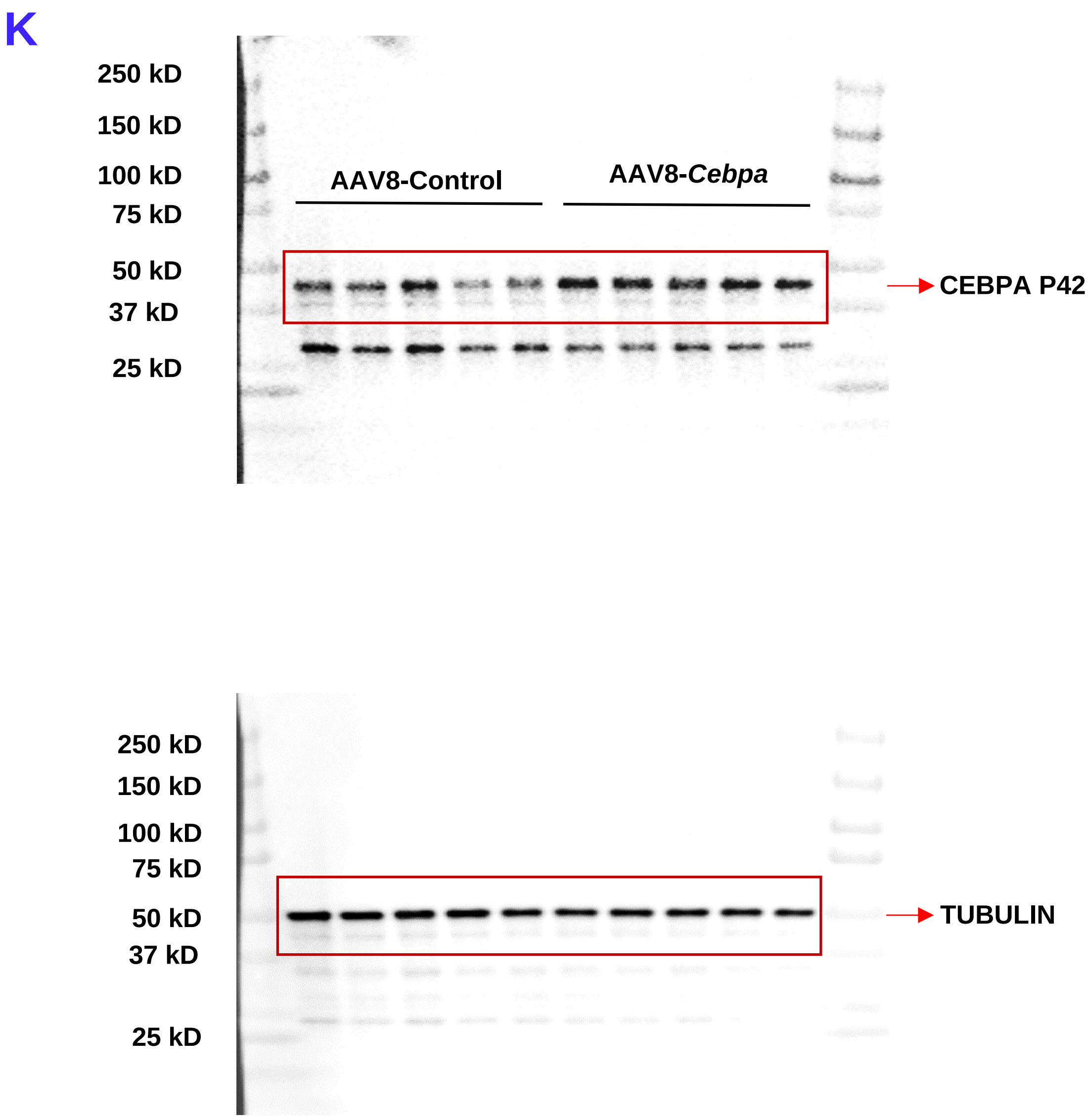
K
250 kD
150 kD
100 kD
75 kD
50 kD
37 kD
25 kD
AAV8-Control
AAV8-*Cebpa*
CEBPA P42
250 kD
150 kD
100 kD
75 kD
50 kD
37 kD
25 kD
TUBULIN

L

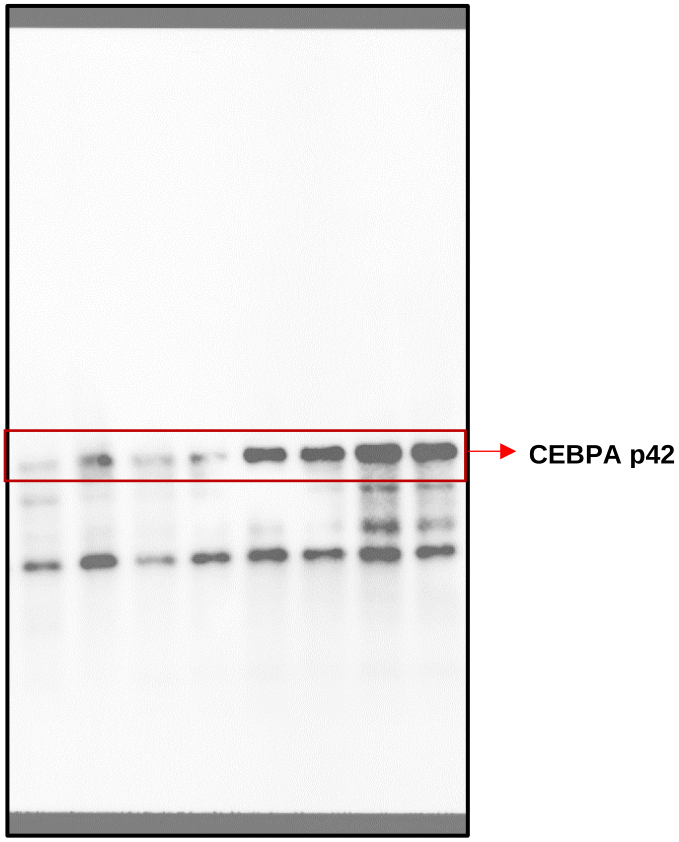

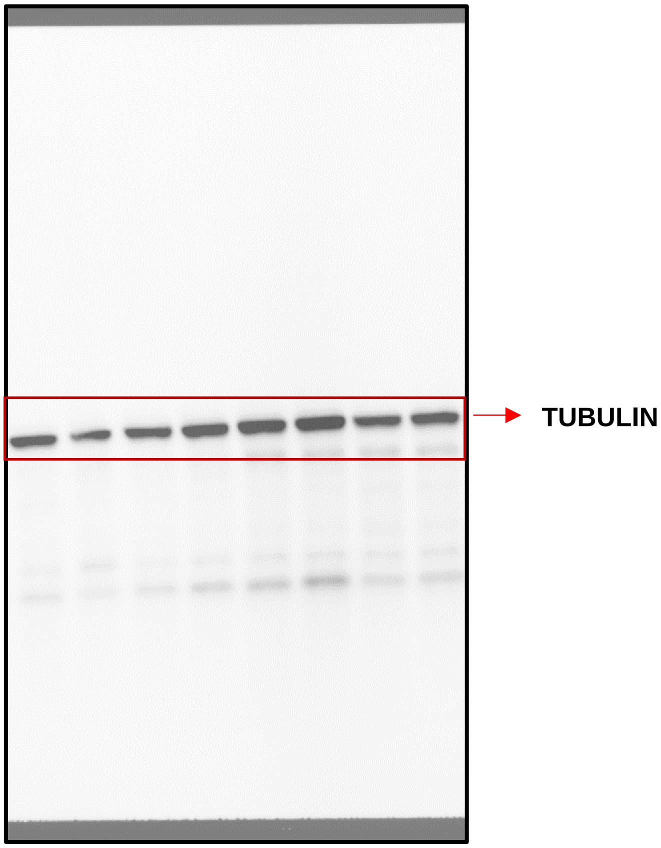

M

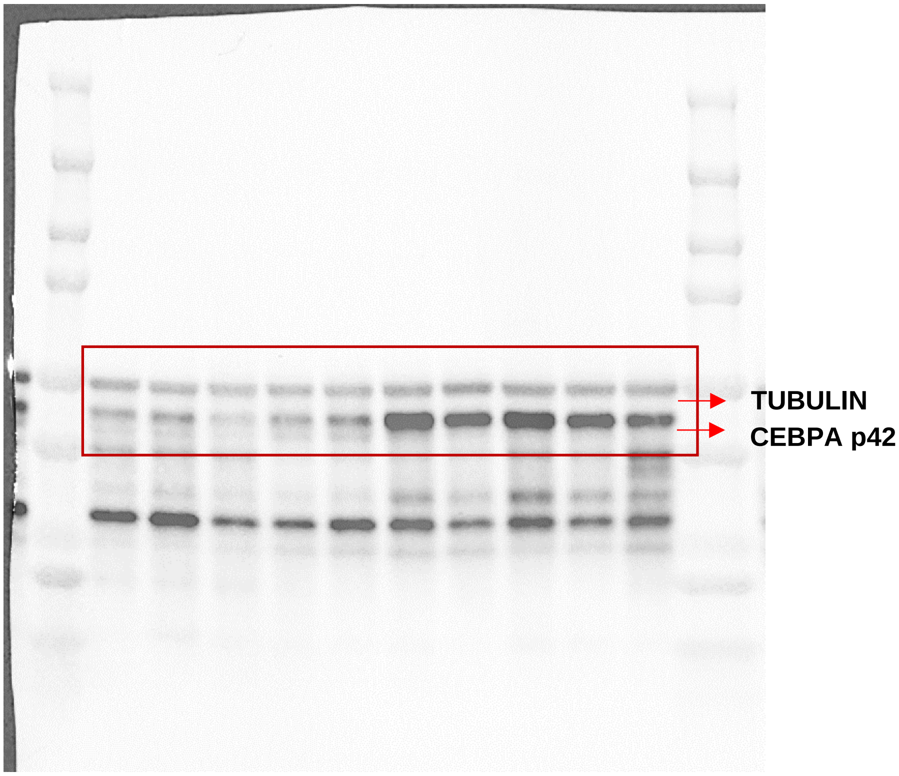

Supplement: Unedited blot and gel images [file jci-134-166731-s135.pdf]
